# Pharmacokinetic model-based assessment of factor IX prophylaxis treatment regimens in severe hemophilia B

**DOI:** 10.1038/s41598-024-70784-x

**Published:** 2024-09-04

**Authors:** Björn Vandewalle, Giancarlo Castaman, Maria Teresa Álvarez-Román, Carmen Escuriola Ettingshausen, László Nemes, Radovan Tomic, Paulo Martins, Joana F. Rodrigues, Karen Pinachyan

**Affiliations:** 1 Exigo Consultores, Lisbon, Portugal; 2 Center for Bleeding Disorders, Careggi University Hospital, Florence, Italy; 3 Department of Hematology, La Paz University Hospital-IdiPaz, Madrid, Spain; 4 Hämophilie Zentrum Rhein Main (HZRM), Mörfelden-Walldorf, Germany; 5 National Haemophilia Centre and Haemostasis Department, Central Hospital of Northern Pest—Military Hospital, Budapest, Hungary; 6 CSL Behring, Milan, Italy; 7 CSL Behring, Marburg, Germany; 8 CSL Behring, Brussels, Belgium

**Keywords:** Haematological diseases, Drug therapy

## Abstract

An important aspect of improving care for people with hemophilia B (HB) is developing optimal treatment strategies. Here we aimed to provide in-silico evidence, comparing the estimated optimal posology of factor IX (FIX) products to support the patient-physician decision-making process. A population pharmacokinetic (popPK) model-based assessment comparing the performance of FIX products (rFIX, rIX-FP, rFIXFc, N9-GP) was developed. PopPK analyses were used to determine a product’s optimal posology to target predefined steady-state FIX activity trough levels in a hypothetical population of 10,000 people with severe HB. Model-derived optimal posologies were compared across several parameters including trough levels, proportion of patients per regimen and consumption, considering 64 hypothetical patient scenarios of different FIX trough level targets and ages. Results indicated a marked difference between FIX products estimated to achieve target trough levels, consumption and dosing frequencies. rIX-FP was associated with higher trough levels than rFIX and rFIXFc, at a lower weekly dose and administration frequency, across all age groups. N9-GP use in adolescents and adults was associated with lower consumption compared with rIX-FP. Insights from this study may be utilized by clinicians to inform decision-making, by considering the model-generated estimated optimal posologies alongside multiple clinical factors and patient preferences.

## Introduction

In people with hemophilia B (HB), prophylaxis with coagulation factor IX (FIX) replacement therapy is recommended to prevent bleeding and musculoskeletal damage due to FIX deficiency^1^. Non-factor replacement treatment therapies, such as anti-tissue factor pathway inhibitor therapies or small interfering RNA targeting antithrombin, are in development for hemophilia B patients and these treatments may be approved in the future^1^. Factor replacement therapy can be administered as standard half-life (SHL) or extended half-life (EHL) recombinant FIX (rFIX). EHL therapies are increasingly recommended in guidelines, as they require fewer infusions to achieve high factor levels with longer dosing intervals, which reduces the treatment burden compared with SHL products^1–4^. Furthermore, EHLs are associated with improved bleeding prevention, quality of life and patient adherence, compared with SHL therapies^3–5^.

Half-life is not representative of the overall pharmacokinetic (PK) profile of a FIX concentrate and may lead to unreliable conclusions when comparing products^6–8^. A multifactorial approach to product comparison should be used as half-life can be affected by drug distribution fluctuations and clearance rate^6^. Typically, trough FIX levels measured just before each administration are used to direct prophylaxis treatment decisions^9^. Traditionally, in severe HB (FIX level < 1 IU/dL) prophylaxis aims to maintain factor levels > 1%. However, since the exact correlation of steady-state trough levels with bleeding phenotype is not fully understood, optimal target trough levels for effective bleeding prevention in individual patients are still under debate^1,9,10^. The 2020 World Federation of Hemophilia guidelines for the management of hemophilia recommend target trough levels of > 3–5% as increasing evidence indicates 1–3% trough levels are insufficient^1^. Lack of target trough level understanding may hinder personalized treatment and optimal resource utilization and increase costs for healthcare systems^11^.

A comparison of potential target trough level attainability is clinically useful when evaluating available FIX therapies. This information used alongside clinical factors (e.g. bleeding phenotype, prior prophylactic consumption, joint status, individual PK characteristics) and patient preferences (e.g. treatment burden, activity levels, lifestyle) may help inform and determine individual dosing schedules. There is a need for resources which can adequately compare the optimal posology (i.e., the dosing schedule to reach a predefined target steady state FIX activity trough level using the lowest possible dose and administration frequency) of FIX products in patients with severe HB.

Population PK analyses are a suitable resource for PK-guided dosing as they allow integration of relevant PK data simultaneously across a range of doses, to determine factors affecting drug exposure (sources of PK variability) so that differences among individuals can be identified^12,13^. Identifying PK variability can inform dosing strategies in patient subpopulations, minimizing the risk of over- or under-dosing^12,13^. Population PK analyses are also useful for establishing the potential adequacy and therapeutic range of individual products^8^. Previous work has been limited; the patient tailOred PharmacokineTIc-guided dosing of CLOTting factor concentrates in clotting disorders (OPTI-CLOT) research program focused on the feasibility of PK-guided dosing of factor replacement therapies and improving PK models. The study highlighted that both half-life and exposure should be considered when selecting optimal treatments; however, the dosing schedules used were neither based on respective European Medicines Agency (EMA) Summary of Product Characteristics (SmPC) nor clinically suitable^11,14^.

This study aimed to create a model-based assessment to compare the performance of four FIX products widely used in the treatment of severe HB, using population PK analyses to determine their estimated optimal posology, within the clinically relevant boundaries of the EMA-approved SmPC dosing and administration frequency, to target predefined steady-state FIX activity trough levels in a hypothetical patient population with severe HB. The population PK model-based assessment compared one SHL-FIX therapy, recombinant FIX (rFIX, BeneFIX®, Pfizer) and three EHL therapies: a recombinant FIX fusion protein with albumin (rIX-FP, Idelvion®, CSL Behring), a recombinant FIX fusion protein with immunoglobulin G1 (rFIXFc, Alprolix®, Swedish Orphan Biovitrum AB) and a glycoPEGylated recombinant FIX (N9-GP, Refixia®, Novo Nordisk A/S). We also present an example case to demonstrate how the model may help inform clinical decisions.

## Results

Estimated optimal posology outcomes for a target trough level of 5% with a control trough level of 10% in patients aged ≥ 12 years (i.e. adults and adolescents) and for target trough levels of 5% and 10% in patients < 12 years (children and infants) are reported below (see Supplementary Results for all other specified target and control trough level outcomes). The scenarios reported in detail were selected by the authoring clinicians, based on clinical relevance and current guidelines.

### Adults

In adults with a target trough level of 5% and a control trough level of 10%, a higher median trough level with lower consumption is achieved for rIX-FP (7.9 IU/dL, 35.0 IU/kg/week) compared with rFIXFc (WP: 4.6 IU/dL, 88.0 IU/kg/week; IP: 4.0 IU/dL, 87.5 IU/kg/week) and rFIX (2.8 IU/dL, 182.0 IU/kg/week) (Table 1). A higher median trough level was also reached with rIX-FP compared with N9-GP although at the expense of higher consumption (7.9 vs. 5.1 IU/dL and 35.0 vs. 12.5 IU/kg/week). Only 0.4% of rIX-FP prophylaxis patients failed to reach the 5% trough level target, compared with 59.3% of rFIXFc WP patients, 71.9% of rFIXFc IP patients and 79.9% of rFIX patients. No N9-GP prophylaxis patients fell below the trough target level. Longer dose intervals were seen with rIX-FP than other products (14 days for 38.3% of patients vs. 3 days for 91.7% of rFIX patients, 7 days for 100.0% of rFIXFc WP patients, 8 days for 87.4% of rFIXFc IP patients and 7 days for 100.0% of N9-GP patients). The median ratio between weekly rIX-FP dose (IU/kg) (1) and other products was also calculated; N9-GP (0.4), rFIXFc WP (2.5), rFIXFc IP (2.5) and rFIX (5.2).

**Table 1:** Optimal posology results – Adults group (≥ 18 years); 5% target trough level with 10% control trough level.

Parameter	rIX-FP	rFIX	rFIXFc WP	rFIXFc IP	rFIXFc* WP and IP	N9-GP
Steady-state FIX trough levels, IU/dL Median (IQR)	7.9 (6.2–9.2)	2.8 (1.6–4.5)	4.6 (3.6–5.0)	4.0 (3.0–5.1)	4.4 (3.4–5.0)	5.1 (5.1–5.2)
Patients below target, %	0.4	79.9	59.3	71.9	63.4	0.0
Dose (IU/kg per week) Median (IQR)	35.0 (25.0–35.0)	182.0 (182.0–182.0)	88.0 (75.5–88.0)	87.5 (87.5–87.5)	87.5 (80.5–88.0)	12.5 (10.0–15.0)
Patients among dose-intervals, %						
≤ 7 days	35.9	100.0	100.0	0.0	68.6	100.0
8–10 days	17.1	0.0	0.0	98.2	30.8	0.0
11–14 days	38.3	0.0	0.0	1.8	0.6	0.0
15–21 days	8.7	0.0	0.0	0.0	0.0	0.0
Ratio between weekly dose (IU/kg) FIX product and rIX-FP Median (IQR)	-	5.2 (5.2–7.3)	2.5 (2.3–3.3)	2.5 (2.5–3.5)	2.5 (2.5–3.5)	0.4 (0.3–0.5)

*Patients were randomly assigned to individualized interval prophylaxis from a Bernoulli distribution with a probability 0.315, based on data from clinical trial 998HB102 (NCT01027364). In the simulations underlying the results of this table, 68.6% of the simulated patients were assigned to receive weekly prophylaxis and 31.4% to individualized interval prophylaxis.

FIX, factor IX; IP, individualized interval prophylaxis; IQR, interquartile range; IU, international units; WP, weekly prophylaxis.

Similar estimated optimal posology results were obtained for adults with a target trough level of 15% and a control trough level of 20% (see Supplementary Results for this age group).

### Adolescents

Similar to adult patients, in adolescents with a target trough level of 5% and a control trough level of 10% rIX-FP achieves a higher median trough level with lower consumption (7.7 IU/dL, 35.0 IU/kg/week) compared with rFIXFc (WP: 4.4 IU/dL, 88.0 IU/kg/week; IP: 3.9 IU/dL, 87.5 IU/kg/week) and rFIX (2.6 IU/dL, 182.0 IU/kg/week) (Table 2). A higher median trough level was reached with rIX-FP compared with N9-GP, with greater consumption (7.7 vs. 5.1 IU/dL and 35.0 vs. 12.5 IU/kg/week). A small proportion of patients (1.2%) on rIX-FP prophylaxis failed to reach the 5% trough level target, compared with 63.8% of rFIXFc WP patients, 75.3% of rFIXFc IP patients and 83.0% of rFIX patients. No N9-GP prophylaxis patients fell below the trough target level. Longer dose intervals were seen with rIX-FP than other products (14 days for 35.9% of patients vs. 3 days for 93.2% of rFIX patients, 7 days for 100.0% of rFIXFc WP patients, 8 days for 89.9% rFIXFc IP patients and 7 days for 100.0% of N9-GP patients). The median ratio between weekly rIX-FP dose (IU/kg) (1) and other products was calculated; N9-GP (0.4), rFIXFc WP (2.5), rFIXFc IP (2.5) and rFIX (5.2).

**Table 2:** Optimal posology results—Adolescents group (12–17 years); 5% target trough level with 10% control trough level.

**Parameter**	**rIX-FP**	**rFIX**	**rFIXFc** WP	**rFIXFc** IP	rFIXFc* WP and IP	**N9-GP**
Steady-state FIX trough levels, IU/dL Median (IQR)	7.7 (6.0–9.0)	2.6 (1.5–4.2)	4.4 (3.4–5.0)	3.9 (2.9–5.0)	4.2 (3.3–5.0)	5.1 (5.1–5.2)
Patients below target, %	1.2	83.0	63.8	75.3	67.3	0.0
Dose (IU/kg per week) Median (IQR)	35.0 (25.0–35.0)	182.0 (182.0–182.0)	88.0 (79.4–88.0)	87.5 (87.5–87.5)	87.5 (84.5–88.0)	12.5 (10.0–15.5)
Patients among dose-intervals, %^†^						
≤ 7 days	47.4	100	100.0	0.0	68.8	100.0
8–10 days	16.7	0.0	0.0	98.6	30.9	0.0
11–14 days	35.9	0.0	0.0	1.4	0.4	0.0
15–21 days	0.0	0.0	0.0	0.0	0.0	0.0
Ratio between weekly dose (IU/kg) FIX product and rIX-FP Median (IQR)	-	5.2 (5.2–7.3)	2.5 (2.4–3.3)	2.5 (2.5–3.5)	2.5 (2.5–3.5)	0.4 (0.3–0.5)

*Patients were randomly assigned to individualized interval prophylaxis from a Bernoulli distribution with a probability 0.315, based on data from clinical trial 998HB102 (NCT01027364). In the simulations underlying the results of this table, 68.8% of the simulated patients were assigned to receive weekly prophylaxis and 31.3% to individualized interval prophylaxis. †Percentages may not sum to 100 due to rounding.

FIX, factor IX; IP, individualized interval prophylaxis; IQR, interquartile range; IU, international units; WP, weekly prophylaxis.

### Children

In children with a target trough level of 5%, rIX-FP again achieves a higher median trough level with reduced consumption (7.1 IU/dL, 35.0 IU/kg/week) compared with rFIXFc (4.3 IU/dL, 100.0 IU/kg/week) and rFIX (2.0 IU/dL, 182.0 IU/kg/week) (Table 3). Overall, 6.5% of children on rIX-FP did not reach the 5% target trough level, compared with 67.5% of children on rFIXFc and 89.6% of children on rFIX. Both rIX-FP and rFIXFc were dosed at longer intervals compared with rFIX (7 days for 100.0% of patients vs. 3 days for 96.4% of patients) although this was expected based on the SmPC. The median ratio between weekly rIX-FP dose (IU/kg) (1) and other products was calculated; rFIXFc (2.9) and rFIX (5.2).

**Table 3:** Optimal posology results—Children and Infant group results at 5% target trough level.

Parameter	rIX-FP	rFIX	rFIXFc
Children group (6–11 years)*			
** Steady-state FIX trough levels, IU/dL** ** Median (IQR)**	7.1 (5.1–9.8)	2.0 (1.1–3.4)	4.3 (3.4–5.0)
** Patients below target, %**	6.5	89.6	67.5
** Dose, IU/kg/ week** ** Median (IQR)**	35.0 (35.0–35.0)	182.0 (182.0–182.0)	100.0 (93.0–100.0)
** Patients among dose-intervals, %**	7d: 100.0	3d: 96.4	7d: 100.0
		3.5d: 2.5	
		4d: 1.1	
** Ratio between FIX product weekly dose (IU/kg) and rIX-FP** ** Median (IQR)**	–	5.2 (4.7–5.2)	2.9 (2.3–2.9)
Infant group (0–5 years)*			
** Steady-state FIX trough levels, IU/dL** ** Median (IQR)**	5.1 (4.6–6.1)	1.9 (1.0–3.0)	3.5 (2.7–4.3)
** Patients below target, %**	28.6	89.2	87.3
** Dose, IU/kg/week** ** Median (IQR)**	40.0 (35.0–50.0)	238.0 (238.0–238.0)	100.0 (100.0–100.0)
** Patients among dose-intervals, %**	7d: 100.0	3d: 96.2	7d: 100.0
		3.5d: 2.7	
		4d: 1.0	
		5d: 0.1	
		6d: 0.0	
		7d 0.0	
** Ratio between FIX product weekly dose (IU/kg) and rIX-FP** ** Median (IQR)**	-	5.7 (4.8–6.8)	2.4 (2.0–2.9)

*At the time of the study, N9-GP was not approved for use in patients less than 12 years old, therefore N9-GP model simulations were not generated for children (6–11 years) or infant (0–5 years) groups.

d, days; FIX, factor IX; IQR, interquartile range; IU, international unit.

Similarly, for children with a target trough level of 10%, rIX-FP achieves a higher median trough level with reduced consumption (10.0 IU/dL, 46.0 IU/kg/week) than rFIXFc (4.3 IU/dL, 100.0 IU/kg/week) and rFIX (2.0 IU/dL, 182.0 IU/kg/week) (Table 4). In total, 40.6% of children on rIX-FP did not reach the 10% target level, compared with 99.2% of children on rFIX and 99.5% of children on rFIXFc. At this target trough level, rIX-FP and rFIXFc were dosed at 7-day intervals and rFIX had shorter intervals of 3 days for 99.9% of patients) in line with the SmPCs. The median ratio between weekly rIX-FP dose (IU/kg) (1) and other products was calculated; rFIXFc (2.2) and rFIX (4.0).

**Table 4:** Optimal posology results—Children and Infant group results at 10% target trough level.

Parameter	rIX-FP	rFIX	rFIXFc
Children group (6–11 years)*			
Steady-state FIX trough levels, IU/dL Median (IQR)	10.0 (8.1–10.1)	2.0 (1.1–3.4)	4.3 (3.4–5.4)
Patients below target, %	40.6	99.2	99.5
Dose, IU/kg/ week Median (IQR)	46.0 (35.5–50.0)	182.0 (182.0–182.0)	100.0 (100.0–100.0)
Patients among dose-intervals, %	7d: 100.0	3d: 99.9	7d: 100.0
		3.5d: 0.1	
		4d: 0.0	
Ratio between FIX product weekly dose (IU/kg) and rIX-FP Median (IQR)	-	4.0 (3.6–5.1)	2.2 (2.0–2.8)
Infant group (0–5 years)*			
Steady-state FIX trough levels, IU/dL Median (IQR)	6.8 (4.6–9.6)	1.9 (1.0–3.3)	3.5 (2.7–4.3)
Patients below target, %	77.3	99.2	100.0
Dose, IU/kg/week Median (IQR)	50.0 (50.0–50.0)	238.0 (238.0–238.0)	100.0 (100.0–100.0)
Patients among dose-intervals, %	7d: 100.0	3d: 99.8	7d: 100.0
		3.5d: 0.2	
		4d: 0.0	
		5d: 0.0	
		6d: 0.0	
		7d: 0.0	
Ratio between FIX product weekly dose (IU/kg) and rIX-FP Median (IQR)	-	4.8 (4.8–4.8)	2.0 (2.0–2.0)

*At the time of the study, N9-GP was not approved for use in patients less than 12 years old, therefore N9-GP model simulations were not generated for children (6–11 years) or infant (0–5 years) groups.

d, days; FIX, factor IX; IQR, interquartile range; IU, international unit.

### Infants

In infants, at a target trough level of 5%, rIX-FP also achieves a higher median trough level with reduced consumption (5.1 IU/dL, 40.0 IU/kg/week) compared with rFIXFc (3.5 IU/dL, 100.0 IU/kg/week) and rFIX (1.9 IU/dL, 238.0 IU/kg/week) (Table 3). Overall, 28.6% of infants on rIX-FP did not reach the 5% target trough level, compared with 87.3% of infants on rFIXFc and 89.2% of infants on rFIX. Like in children, both rIX-FP and rFIXFc were dosed at longer intervals compared with rFIX (7 days for 100.0% of patients vs. 3 days for 96.2% of patients) in line with the SmPCs. The median ratio between weekly rIX-FP dose (IU/kg) (1) and other products was calculated; rFIXFc (2.4) and rFIX (5.7).

A similar result was seen in infants at a target trough level of 10%; rIX-FP achieves a higher median trough level with reduced consumption (6.8 IU/dL, 50.0 IU/kg/week) compared with rFIXFc (3.5 IU/dL, 100.0 IU/kg/week) and rFIX (1.9 IU/dL, 238.0 IU/kg/week) (Table 4). In total 77.3% of infants on rIX-FP did not reach the 10% target trough level, compared with 100.0% of infants on rFIXFc and 99.2% of infants on rFIX. Again, both rIX-FP and rFIXFc were dosed at longer intervals compared with rFIX (7 days for 100.0% of patients vs. 3 days for 99.8% of patients) in line with the SmPCs. The median ratio between weekly rIX-FP dose (IU/kg) (1) and other products was calculated; rFIXFc (2.0) and rFIX (4.8).

## Discussion

The population PK model-based assessment reported in this study will provide clinicians with in-silico insights to better understand the different FIX therapy capabilities and clinically relevant estimated optimal dosing strategies, thus, enabling informed treatment decisions contributing to personalized therapy for people with severe HB. The model estimates the ability of different FIX therapies to achieve target trough levels and the dosing schedule necessary to achieve this. Specifically, this model suggests that some FIX products can consistently reach adequate target trough levels together with a reduced treatment burden and lower consumption compared with others. This model also shows that some FIX therapies are either unable to reach certain targets or can do so only in a small proportion of patients.

The model indicates that rIX-FP is associated with higher trough levels than rFIX and rFIXFc, at a markedly lower weekly dose and administration frequency, across all age groups, which was to be expected. However, N9-GP use in adolescents and adults is associated with lower consumption compared with rIX-FP, although more frequent infusions are often needed. Moreover, this model indicates that among the 10,000 variable patient simulations, it is highly unlikely to reach a 10% trough level with infants. This knowledge should inform dosing strategies for this patient subpopulation to minimize the risk of overdosing by not attempting to achieve unrealistic target trough levels.

Although bleeding phenotype and trough levels do not always correlate, this PK model-based assessment may help reduce treatment burden, resource utilization and costs, while maintaining or lowering bleeding risk in people with severe HB by providing estimated optimal posologies for FIX therapies. In contrast to the study by Preijers et al*.*, this model considered posologies aligned with each therapy’s SmPC, making them appropriate for application in clinical practice^14^.

While half-life influences the capabilities of a FIX therapy, other parameters must be considered when determining an appropriate treatment and dosing schedule, such as area under the curve, time above target and peak level. These parameters allow identification of differences in the ability to achieve high trough levels for optimal bleeding control and minimal treatment burden, in terms of administration frequency and FIX consumption^6,15^. Alongside PK parameters, a patient’s preference and lifestyle choices should be taken into consideration. This model will provide clinicians with new in-silico evidence to estimate the optimal posology for a range of FIX therapies that can be considered alongside individual patient factors (e.g. personal preferences, lifestyle, age, arthropathy) to help inform treatment decisions and regimens. Real-life trough levels generated from a single-dose PK assessment can then validate the model outputs and the dosing schedule may be adjusted based on a patient’s PK values.

This study is not without limitations. The population PK models used are derived from four independent articles^12,13,16,17^, without endogenous baseline FIX activity simulation. However, this allowed us to evaluate trough levels achievable exclusively because of the factor therapies considered. This limitation is mitigated by PK profile simulation of the four FIX therapies in the same 10,000 hypothetical patients, with respect to age and weight. Additionally, age-dependent body weight was used to establish dosing due to there being no published literature utilizing lean body weight. The population PK for rIX-FP, rFIX and N9-GP are described by a two-compartment model, whereas rFIXFc is described by a three-compartment model, which may have some indirect effect on the results of this study; a three-compartment model has the potential to more accurately model the tail of the FIX activity levels over time, which underlies all outcomes measured in this study. When considering applications in clinical practice, in some cases the results from this study may not reflect real-life scenarios. For example, in this model, the calculated weekly dose of N9-GP was 12.5 IU/kg (dosing range 10–169 IU/kg), although the EU SmPC and Canadian real-world usage suggest 40 and 33–39 IU/kg/week (based on 1716–2045 IU/kg median annualized consumption) respectively^18–20^. At the time of this study, N9-GP was not approved for use in patients less than 12 years old, therefore N9-GP model simulations were only generated in adults (≥ 18 years) and adolescents (12–17 years). However, we acknowledge that in August 2023, following completion of this study, the European Commission granted a label extension for N9-GP to include treatment of patients of any age. Therefore, future N9-GP model simulations among children less than 12 years old may be of value. Moreover, as the objective of this study was to assess optimal posologies in the European context, the analysis was based on the existing EU SmPCs. As such, the results of this study may not be generalizable to non-EU countries if there are significant differences in the approved labels. Additionally, it was not possible to incorporate the extravascular distribution of FIX in this model, however, although the concept of measuring extravascular distribution is scientifically interesting, the clinical relevance is still relatively unknown^21^. Similarly, another current topic of interest in the hemophilia field is that of discrepancies between one-stage clotting assays and chromogenic substrate assays to measure plasma FIX activity, however, considering the base studies used to develop this model all used one-stage clotting assays this was not considered to be a notable limitation. Finally, some common limitations related to the nature of the in-silico study are relevant, such as the small sample size of the independent studies, differences in study methods such as reagents used for clotting assays, and comparisons between such studies, used to produce the population PK model.

This study has several strengths including that we assessed a wide range of target and control trough levels, reflecting the current and evolving future standard of care. Additionally, this model estimates cumulative dimensions of interest in treatment decision-making, including dosing, administration frequency, trough level achieved, patients remaining below target, and patients moving to longer administration intervals. The posology considered in this analysis is based on existing SmPCs, providing clinically relevant scenarios for supporting treatment decisions when used alongside clinical patient evaluation, including individual bleeding phenotype and both peak and trough FIX activity levels. The model-generated dosing posologies can be used to estimate consumption of FIX therapies and the associated cost implications. Lastly, this model has the potential to be adapted to utilize individual patient body weights and may be explored in future studies to further optimize individual PK-guided dosing strategies. We present an example case below to demonstrate how the model may help inform clinical decision-making.

### Utilizing the model to inform decisions in a clinical scenario – example case study

A clinician wants to determine an appropriate FIX therapy and dosing schedule for a 22-year-old male patient with severe HB who enjoys participating in sports regularly and has an active social life. He prefers less frequent infusions to reduce his treatment burden.

Before selecting a FIX therapy, the clinician may refer to the PK model to obtain predictions of the patient achieving a target trough level, along with the dosing schedule necessary to achieve this.

Using the model, the clinician can assess which FIX therapy is most likely to reach a target trough level and control level in adult patients (≥ 18 years) out of 25 hypothetical adult scenarios **(**Table 5**)**. If the clinician refers to Table 1, this will provide the model-generated optimal posology results for adults (≥ 18 years) achieving a 5% target trough level with a 10% control trough level. The clinician will also be able to assess the necessary dose and administration frequency required to reach 5% trough levels for each FIX therapy. As the clinician is aware that the patient has high activity levels and prefers fewer infusions, the clinician can consider this and select an appropriate FIX therapy that has a high probability of achieving a 5% target with lower administration frequencies.

**Table 5:** Target and control trough level scenarios for adolescents and adult populations subject to prophylactic treatment with rIX-FP and rFIXFc (empty cells).

		Control trough level				
		5%	7.5%	10%	15%	20%
Target trough level	1%					
	2%					
	3%					
	5%	N/A				
	7.5%	N/A	N/A			
	10%	N/A	N/A	N/A		
	15%	N/A	N/A	N/A	N/A	

N/A, not applicable.

The clinician can make an informed decision to select a FIX therapy and dosing schedule, based on the model’s outputs estimating optimal posology alongside clinical factors and patient preferences. An individual single-dose PK assessment can then be carried out to validate whether the real-life trough levels are aligned with the model’s estimated optimal posology. The dosing schedule may then be adjusted based on the patient’s PK assessment outcomes.

## Conclusions

This population PK model-based multi-dimensional assessment can provide additional in-silico insights into optimal dosing posologies (i.e., the dosing schedule to reach a target predefined steady state FIX activity trough level using the lowest possible dose and administration frequency) with FIX therapies for prophylaxis in people with severe HB to inform joint patient-physician decision-making. Clinical utilization of this model can aid treatment decisions by providing insights into the probability of achieving selected FIX activity trough levels, when patient preferences and needs are taken into consideration alongside their clinical profile. Subsequently, clinical decisions informed by such in-silico perspectives may support improvements in clinical outcomes, treatment burden, patient satisfaction, resource utilization and overall costs.

## Methods

### Population PK model simulations

For hypothetical people with severe HB, individual quasi-continuous steady-state FIX activity levels as a function of time were produced, through Monte Carlo simulation, for the four FIX therapies. Simulations were performed with AnyLogic v7.3.7 based on published population PK models (Table 6 and **Supplementary Table S1**), identified via a systematic literature review (SLR)^12,13,16,17^. Details on the SLR and selection criteria are outlined in the Supplementary Methods. Variability in steady-state FIX activity level profiles for hypothetical patients was obtained by considering the structural model components—typically dependent on body weight—and the inter-individual variability components of the selected population PK models. Inter-occasion or other residual variability was not considered. Since severe HB is characterized by an endogenous baseline FIX level < 1 IU/dL and only the population PK model for rIX-FP included a structural parameter to describe baseline FIX levels, endogenous baseline FIX levels were disregarded during simulations^13^. All models were validated against published results of publicly available models^12,13,16,17^. Figures illustrating the simulations process for finding the optimal posology are included within the Supplementary Information (Supplementary Figures S2–4).

**Table 6:** Published population PK models used to simulate steady-state quasi-continuous individual FIX activity levels as a function of time.

	rIX-FP	rFIX	rFIXFc	N9-GP
Published population and PK model characteristics				
Source	Zhang et al.^13^	Suzuki et al.^16^	Diao et al.^17^	Collins et al.^12^
Number of patients	104	201	135	16*
Age range (years)	1–65	0–69	12–77	21–55*
PK model structure	2-compartment	2-compartment	3-compartment	2-compartment

*Values reported in Negrier et al.^27^.

PK, pharmacokinetic.

Given a hypothetical patient’s body weight, with the above population PK models, steady-state FIX activity levels can be simulated for any prophylactic dosing and administration schedule for all considered FIX products. The PK parameter of interest to this study is the steady-state FIX activity trough level, measured just before the administration of a new dose of FIX.

### Body weight and age groups

Age-dependent body weights for individual hypothetical patients were simulated from a logistic growth curve model with a multiplicative error term (equation detailed in the Supplementary Methods). This model was chosen from a set of different parametric growth curve models, as the one with the best fit to the body weight by age data of the study population at the basis of the rIX-FP population PK model^13^ [data on file]. The age distribution considered in this study followed the distribution of the population underlying the growth curve model [data on file]. In line with age cutoffs in European Medicines Agency (EMA)-approved SmPCs of FIX products for the prophylactic treatment of HB, the following age groups were considered: infants (0–5 years), children (6–11 years), adolescents (12–17 years), and adults (≥ 18 years). For each age group, 10,000 individual hypothetical patients were simulated, with different ages and body weights. The mean and standard deviation (SD) of the resulting considered weight distribution, for each age group, can be found in **Supplementary Table S2**.

### Dosing and administration

Dosing and administration frequencies (i.e. dosing schedule) for prophylactic treatment were, whenever possible, directly aligned to recommendations in the posology and method of administration section of the EMA-approved SmPC (Section 4.2) of all FIX products (Table 7). Whenever, in terms of posology, the SmPC left room for interpretation or did not clearly define minimum and maximum boundaries for dosing and administration frequency, secondary sources were consulted with the following hierarchy: the pharmacodynamic properties section of the EMA approved SmPC (Section 5.1), the EMA European Public Assessment Report (EPAR), and published pivotal clinical trials. Reported boundary values were rounded to the nearest integer value, whenever considered realistic, to facilitate simulations.

**Table 7:** Dosing and administration schedules considered during simulations for prophylactic treatment with rIX-FP, rFIX, rFIXFc and N9-GP.

	Administration frequency (Days)	Minimum dose (IU/kg per administration)	Maximum dose (IU/kg per administration)
rIX-FP^a^	*Infants and children (< 12 years of age)*		
	7	35	50
	*Adolescents and adults (≥ 12 years of age)*		
	7	35	50
	10 and 14^b^	50^c^	75
	*Adults (≥ 18 years)*		
	21^b,d^	100^e,c^	100^e,c^
rFIX^f^	*Infants (< 6 years of age)*		
	3, 3.5, 4, 5, 6, 7^g^	25^h^	102^h^
	*Children, adolescents and adults (≥ 6 years of age)*		
	3, 3.5, 4^g^	13	78
rFIXFc^i^	*Infants and children (< 12 years of age)*		
	7	50^j^	100
	*Adolescents and adults (≥ 12 years of age) – Weekly prophylaxis*		
	7	17^j^	88^j^
	*Adolescents and adults (≥ 12 years of age) – Individualized interval prophylaxis*		
	8 to 21^j,k^	100	100
N9-GP^l^	*Adolescents and adults (≥ 12 years of age)*^*m*^		
	7^n^	10^n^	169^n^

^a^Section 4.2 of EMA-approved SmPC^22^; ^b^10, 14 and 21-day interval allowed only for well-controlled patients; ^c^Mancuso 2020^23^; ^d^21-day interval allowed for patients ≥ 18 years; ^e^Section 5.1 of EMA-approved SmPC^22^; ^f^Section 4.2 of EMA-approved SmPC^24^; ^g^Increments between minimum and maximum administration schedule are an assumption; ^h^Mean ± 2SD with mean 63.7 and SD 19.1^24^; ^i^Section 4.2 of EMA-approved SmPC^25^; ^j^EPAR^26^; ^k^One-day increments (assumption); ^l^Section 4.2 of EMA-approved SmPC^19^; ^m^N9-GP was not approved for use in patients less than 12 years old during the time of the study; ^n^EPAR^18^.

For rIX-FP, the SmPC-recommended dosing schedule is 35–50 IU/kg once weekly. Adolescents and adults who are well-controlled on a once-weekly dosing schedule can be treated with up to 75 IU/kg at 10 or 14-day intervals. For adults, this is extendable to a 21-day dosing interval at 100 IU/kg fixed dose^22^. The SmPC of rIX-FP is unclear on the minimum dose for 10 and 14-day dosing intervals. For this study, it was fixed at 50 IU/kg following the protocol of a global Phase 3 extension study on the efficacy and safety of rIX-FP in prophylaxis in previously treated adult and adolescent patients^23^.

The dosing and administration schedule for rFIX in pediatric patients < 6 years old is based on the SmPC-reported mean dose (SD) of 63.7 (19.1) IU/kg at intervals of 3–7 days^24^, assuming a range equal to the mean ± 2SD. For the remaining age groups, the SmPC recommended range of 13–78 IU/kg at intervals of 3–4 days was considered^24^. Besides 1-day increments in administration schedules, a dosing interval of 3.5 days was also considered for all age groups, to allow for a twice-weekly administration.

For rFIXFc, the SmPC recommends a weekly administration schedule for infants and children, with a starting dose of at least 50 IU/kg, allowing higher doses when required, up to 100 IU/kg maximum^25^*.* The 50 IU/kg minimum dose was confirmed by the last prescribed dose in a Phase 3 study in children < 12 years of age, reported in the EPAR^26^. For adolescents and adults, two dosing and administration schedules are recommended in the SmPC: weekly prophylaxis (WP) with variable dosing based on individual response; and individualized interval prophylaxis (IP) (interval adjustable based on individual response) with a fixed dose of 100 IU/kg. For WP, minimum and maximum doses are aligned with the EPAR, reporting a maximum range of 16.7–87.6 IU/kg in a Phase 3 study of rFIXFc in patients ≥ 12 years^26^*.* For IP the same Phase 3 study reported a maximum range in dosing intervals of 7.7–20.8 days^26^*.* The SmPC states that some IP patients who are well controlled on a 10-day regimen might be treated on an interval of 14 days or longer^25^*.*

At the time of the study, the therapeutic indication of N9-GP was limited to the treatment and prophylaxis of bleeding in patients ≥ 12 years, with SmPC recommended posology for prophylaxis of 40 IU/kg once weekly^19^. The final dosing and administration schedule of N9-GP was aligned with the EPAR available at the time of the study, reporting a maximum range of 10–169 IU/kg weekly for most patients on long-term prophylaxis in the extension study of its pivotal clinical trial^18^.

### Target steady-state FIX activity trough levels

For each hypothetical patient and each FIX product, from within the boundaries presented in Table 7, an estimated optimal posology was determined, in terms of dose and dosing interval, to target predefined steady-state FIX activity trough levels (“target trough level”). Target trough levels considered were: 1%, 2%, 3%, 5%, 7.5%, 10% and 15%. The mathematical search algorithm (designed for this study and programmed in AnyLogic) ensures that the optimal posology is such that the hypothetical patient’s steady-state trough level is never lower than the specified target trough level, except for when the target trough level is unreachable even at maximum dose and minimum dosing interval.

For rIX-FP and rFIXFc, certain extended dosing intervals are only recommended for well-controlled patients. In clinical practice, the decision to switch a patient from standard to extended dosing intervals should be at the discretion of the treating physician. This is typically based on the lack of spontaneous bleeds and the impact of regular, sufficient trough levels on a patient’s lifestyle (e.g. social and physical) for a prolonged period before switching. Although the exact relationship between steady-state FIX activity levels and bleeding phenotype is not fully understood, they are generally considered highly correlated, with the spontaneous bleed rate decreasing with increasing FIX activity level. As such, for rIX-FP and rFIXFc, the concept of a “control trough level” was introduced during simulations, as a proxy for the steady-state FIX activity trough level above which a hypothetical patient during simulations is considered well-controlled.

If a hypothetical patient on a minimal standard dose within a certain administration interval, maintains steady-state FIX activity trough level above the control trough level, this patient is allowed to switch to an extended dosing interval and corresponding dose range. Control trough levels considered during simulation were 5%, 7.5%, 10%, 15% and 20%, with the condition of being strictly higher than the prespecified target trough level. For rIX-FP, control trough levels apply to adolescents and adults switching from a 7-day dosing interval at 35 IU/kg to a 10-day dosing interval, and from a 10-day dosing interval at 50 IU/kg to a 14-day dosing interval; and adults to further switch from a 14-day interval at 50 IU/kg to a 21-day interval. For rFIXFc, control trough levels apply to adolescents and adults on IP to switch from a 10-day to a 14-day or longer dosing interval (at a fixed dose of 100 IU/kg).

For infants and children, seven scenarios each were simulated corresponding to specified target trough levels (1%, 2%, 3%, 5%, 7.5%, 10% and 15%). For adolescent and adult populations subject to prophylactic treatment with rIX-FP and rFIXFc a further 25 scenarios each were simulated, considering the different target and control trough level combinations (Table 5); 64 scenarios in total. The target and control trough level options were selected to ensure guideline-recommended levels are met as a minimum, and so targets could be increased during patient-physician discussions, based on patient preference, lifestyle choices and clinical suitability.

### Outcome measures

Within each scenario and hypothetical patient, the estimated optimal posology in terms of dose and dosing interval to reach the corresponding target trough level with each FIX product was collected. For those hypothetical patients for whom the exact target trough level was not attainable (i.e., steady-state FIX activity trough level below target at maximum dose and minimum dosing interval or steady-state FIX activity trough level above target at minimum dose and maximum dosing interval), the actual attained steady-state FIX activity trough level was also collected.

From these individual hypothetical patient data, the following outcome measures at estimated optimal posology were calculated for each FIX product: attained steady-state FIX activity level (median and interquartile range [IQR], mean and SD); percentage of patients below target trough level; average dose (IU/kg) as a weekly equivalent (median and IQR, mean and SD); percentage of patients among different dosing intervals; and the ratio of annualized consumption of rFIX, rFIXFc and N9-GP as compared with the annualized consumption of rIX-FP (median and IQR, mean and SD of the ratio of the consumption of individual hypothetical patients). An EHL product was selected as the comparator as these are more frequently used for the management of severe hemophilia B than SHL products; rIX-FP was selected as the appropriate comparator as this was the first EHL product to be approved by the EMA.

## Supplementary Information

Supplementary Information.

## Data Availability

CSL Behring will only consider requests to share data that are received from systematic review groups or bona-fide researchers. CSL Behring will not process or act on data requests until 12 months after article publication on a public website. A data request will not be considered by CSL Behring unless the proposed research question seeks to answer a significant and unknown medical science or patient care question. Applicable country-specific privacy and other laws and regulations will be considered and may prevent sharing of data. Requests for use of the data will be reviewed by an internal CSL Behring review committee. If the request is approved, and the researcher agrees to the applicable terms and conditions in a data sharing agreement, data that have been appropriately anonymized will be made available. Supporting documents including the study protocol and Statistical Analysis Plan will also be provided. For information on the process and requirements for submitting a voluntary data-sharing request for data, please contact CSL Behring at clinicaltrials@cslbehring.com.
