# Supplementary material for: Pharmacokinetic model-based assessment of factor IX prophylaxis treatment regimens in severe hemophilia B
Source: Sci Rep. 2024 Sep 4;14:20534. doi: 10.1038/s41598-024-70784-x (PMC11372059; doi:10.1038/s41598-024-70784-x)
Supplement: Supplementary file 1 — Supplementary Information. [file 41598_2024_70784_MOESM1_ESM.docx]

**Supplementary Information**

**Supplementary Methods**

***Systematic Literature Review***

Published population PK (popPK) models for each evaluated product (rFIX, rIX-FP, rFIXFc, N9-GP) were identified through a systematic literature review (SLR). An electronic database (MEDLINE^®^, MEDLINE^®^ In-Process Citations, through the Pubmed.com interface, and Cochrane Library) search was conducted on August 19th, 2020. The search algorithms were tailored to each selected database and developed to maximize search sensitivity. Inclusion criteria were people with severe hemophilia B; prophylactic treatment with rFIX, rIX-FP, rFIXFc, or N9-GP; and popPK models of recombinant FIX products; there was no geographic or time restriction and only studies published in English or Portuguese language were selected. Literature reporting on studies of specific patient groups or on-demand and/or surgical and/or perioperative recombinant FIX product use, studies of FIX products other than those considered for the study, animal studies, and SLRs were excluded. Study selection was performed by two reviewers, through duplicate reference removal, and title and abstract, as well as full text, reading of all references that complied with the selection criteria or raised doubt. Lack of consensus on study inclusion/exclusion was resolved by a third reviewer, in line with updated Cochrane Group recommendations [1].

Overall, five publications (Figure S1) reporting on six popPK models were eligible for implementation [2-6]. Among these six models, there was one each for rIX-FP [3], rFIXFc [6], and N9-GP [2]. Three models were identified for rFIX [2, 4, 5], of which the most recent comprising the largest dataset and therefore considered the most robust for the purposes of the current study, was selected. The above selection process resulted in a total of four popPK models [2-4, 6], one for each evaluated recombinant FIX product. These models were used to simulate quasi-continuous individual FIX activity levels over time. The popPK models for rIX-FP, rFIX and N9-GP were described by a two-compartment model [2-4], whereas the popPK model for rFIXFc was described by a three-compartment model [6].

**Growth Curve Model**

Age-dependent body weights for individual hypothetical patients were simulated from a logistic growth curve model with a multiplicative error term using the following equation:

$$\frac{\beta_{1}}{1+\beta_{2} \cdot\exp\left( -\beta_{3} \cdot\min\left( 20, \text{age} \right) \right)}\cdot exp(\varepsilon)$$

where β_1=86.47, β_2=8.03, β_3=0.19 and ε is obtained from a normal distribution, with zero mean and variance 0.04.

**Modelling software**

AnyLogic simulation software details:

The AnyLogic Company (www.anylogic.com)

AnyLogic North America, LLC, Oakbrook Terrace Tower, 1 Tower Lane, Suite 2655 Oakbrook Terrace, IL 60181 USA, (312) 635-3344

**Supplementary Figure S1. SLR results according to PRISMA flowchart**


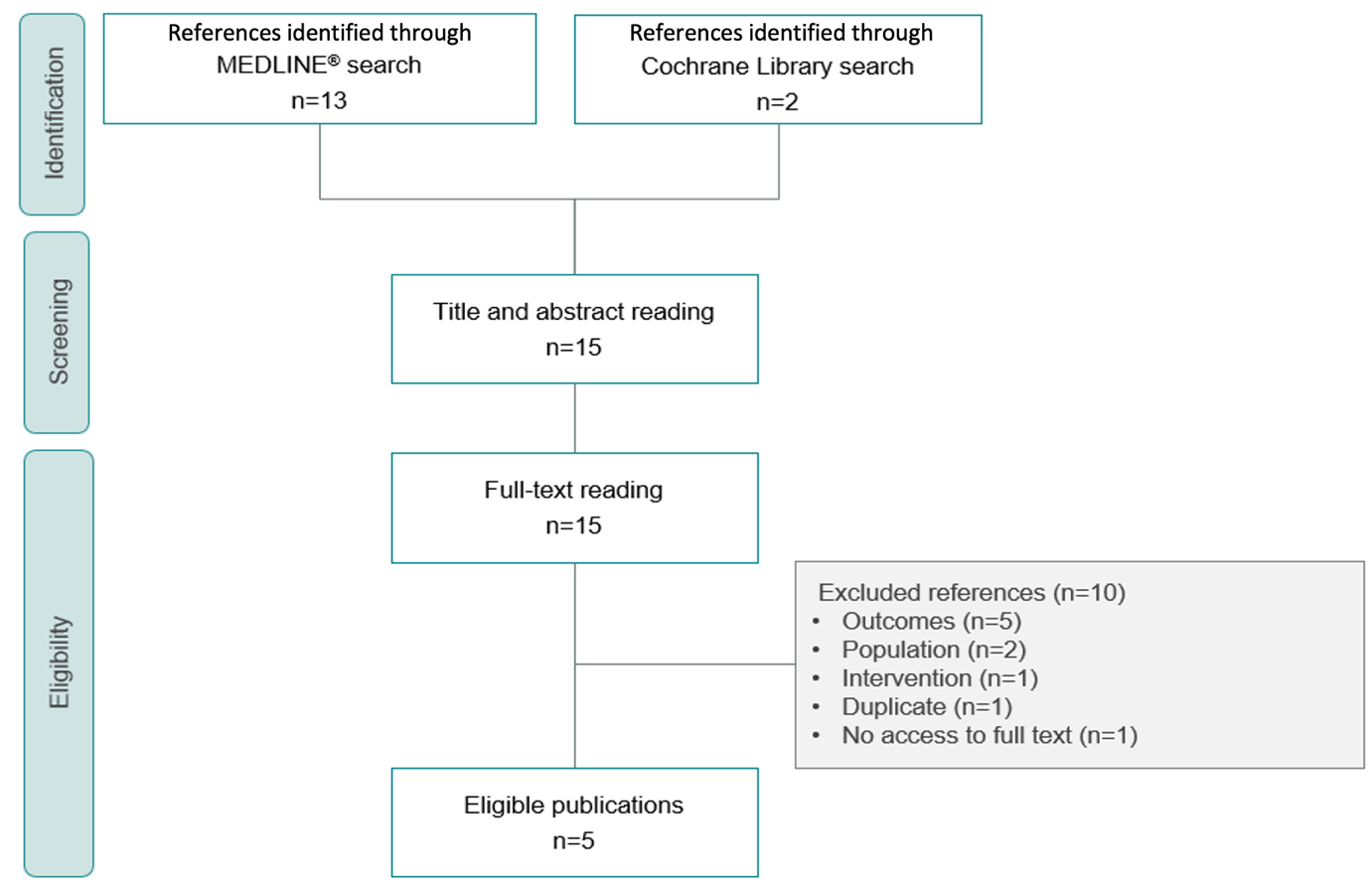


PRISMA, Preferred Reporting Items for Systematic Reviews and Meta-Analyses; SLR, systematic literature review.

**Supplementary Table S1. Published population PK models and parameters used to simulate steady state quasi-continuous individual FIX activity levels as a function of time**

|  | **rIX-FP* [3]** | **rFIX [4]** | **rFIXFc† [6]** | **N9-GP [2]** |
| --- | --- | --- | --- | --- |
| **PK model characteristics** |  |  |  |  |
| PK model structure | 2-compartment | 2-compartment | 3-compartment | 2-compartment |
| **Structural model parameters** |  |  |  |  |
| Clearance central compartment (CL: dL/h) | 0.569 | 5.51 | 2.39 | 0.007§ |
| Volume of distribution central compartment (V1: dL) | 64.8 | 97.7 | 71.4 | 0.739§ |
| Intercompartmental clearance between compartments 1 and 2 (Q2: dL/h) | 0.294 | 5.77 | 1.67 | 0.006§ |
| Volume of distribution peripheral compartment 2 (V2: dL) | 15.8 | 46.2 | 87 | 0.156§ |
| Intercompartmental clearance between compartments 1 and 3 (Q3: dL/h) | - | - | 39.3 | - |
| Volume of distribution peripheral compartment 3 (V3: dL) | - | - | 39.9 | - |
| Weight adjusted dose on V1 | 0.377♯ | - | - | - |
| Body-weight on CL | 0.532 | 0.799 | 0.436 | - |
| Body-weight on V1 | 0.792 | 0.881 | 0.396 | - |
| Body-weight on Q2 | - | 0.741 | - | - |
| Body-weight on V2 | 0.792 | 1.02 | - | - |
| **Inter-individual variability parameters** |  |  |  |  |
| IIV on CL | 0.045 | 0.256^2 | 0.177^2 | 0.168^2 |
| IIV on V1 | 0.067 | 0.232^2 | 0.217^2 | 0.187^2 |
| IIV on Q2 | - | 0.691^2 | 0.358^2 | 1.273^2 |
| IIV on V2 | - | 0.357^2 | 0.462^2 | - |
| IIV on V3 | - | - | 0.377^2 | - |
| Correlation between Q2 and V2 | - | 0.481 | - | - |
| Correlation between CL and V1 | - | - | 0.756 | 0.169 |

CL, clearance central compartment; IIV, inter-individual variability; FIX, factor IX; PK, pharmacokinetics; Q2, intercompartmental clearance between compartments 1 and 2; Q3, intercompartment clearance between compartment 1 and 3; V1, volume of distribution central compartment; V2, volume of distribution peripheral compartment 2; V3, volume of distribution peripheral compartment 3.
^*^ Parameters were normalized for a patient with a body weight of 70 kg; ^†^ Parameters were normalized for a patient with a body weight of 73 kg; ^♯^ The weight-adjusted dose for V1 is 50 IU/kg; ^§^ per kilogram.

**Supplementary Table S2. Summary statistics of the simulated weight distribution of the hypothetical severe hemophilia B population, by age group**

| **Age group** | **Age range** | **Bodyweight (kg)** |
| --- | --- | --- |
|  | (Years) | Mean (SD) |
| Infants | 0–5 | 17.0 (5.7) |
| Children | 6–11 | 34.9 (10.2) |
| Adolescents | 12–17 | 60.2 (14.0) |
| Adults | 18+ | 73.3 (15.4) |

SD, standard deviation.

**Supplementary Figure S2. Flux chart of model simulation for the optimal rIX-FP posology search in patients over 12 years old.**


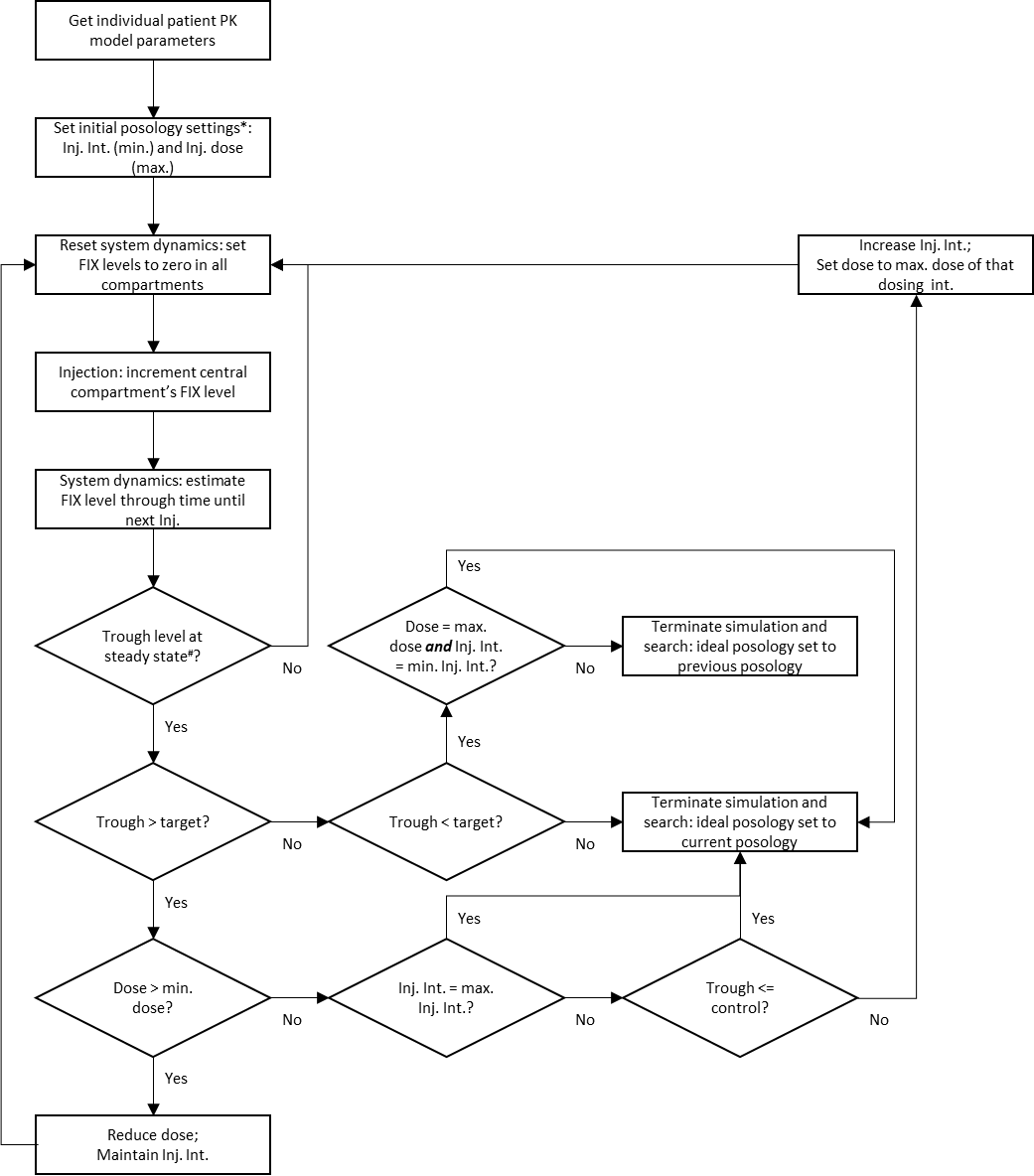


Min: minimum; max: maximum; Inj: injection; Int: interval; PK, pharmacokinetic; FIX, factor IX.

*Initial posology considered the minimum injection interval and the maximum dose for that injection interval; # steady state defined as absolute difference in FIX activity level at the time of two successive injections inferior to 0.01 IU/mL (considering FIX activity levels without/before increase provided by next injection).

**Supplementary Figure S3. Flux chart of model simulation for the optimal rIX-FP posology search in patients under 12 years old.**


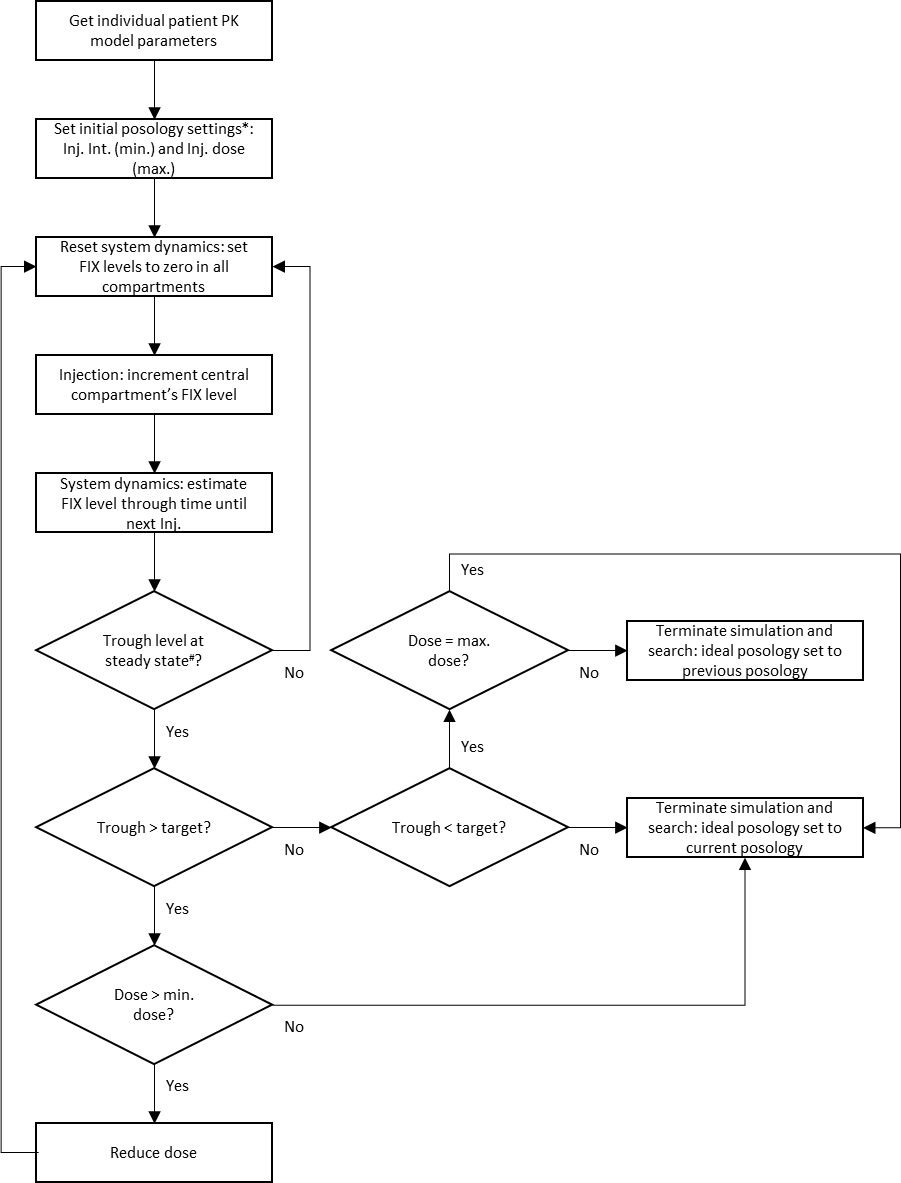


Min: minimum; max: maximum; Inj: injection; Int: interval; PK, pharmacokinetic; FIX, factor IX.

*Injection interval was fixed at 7 days; initial posology considered the maximum dose; # steady state defined as absolute difference in FIX activity level at the time of two successive injections inferior to 0.01 IU/mL (considering FIX activity levels without/before increase provided by next injection).

**Supplementary Figure S4. Example of FIX activity level and weekly dose over time during optimal posology search**


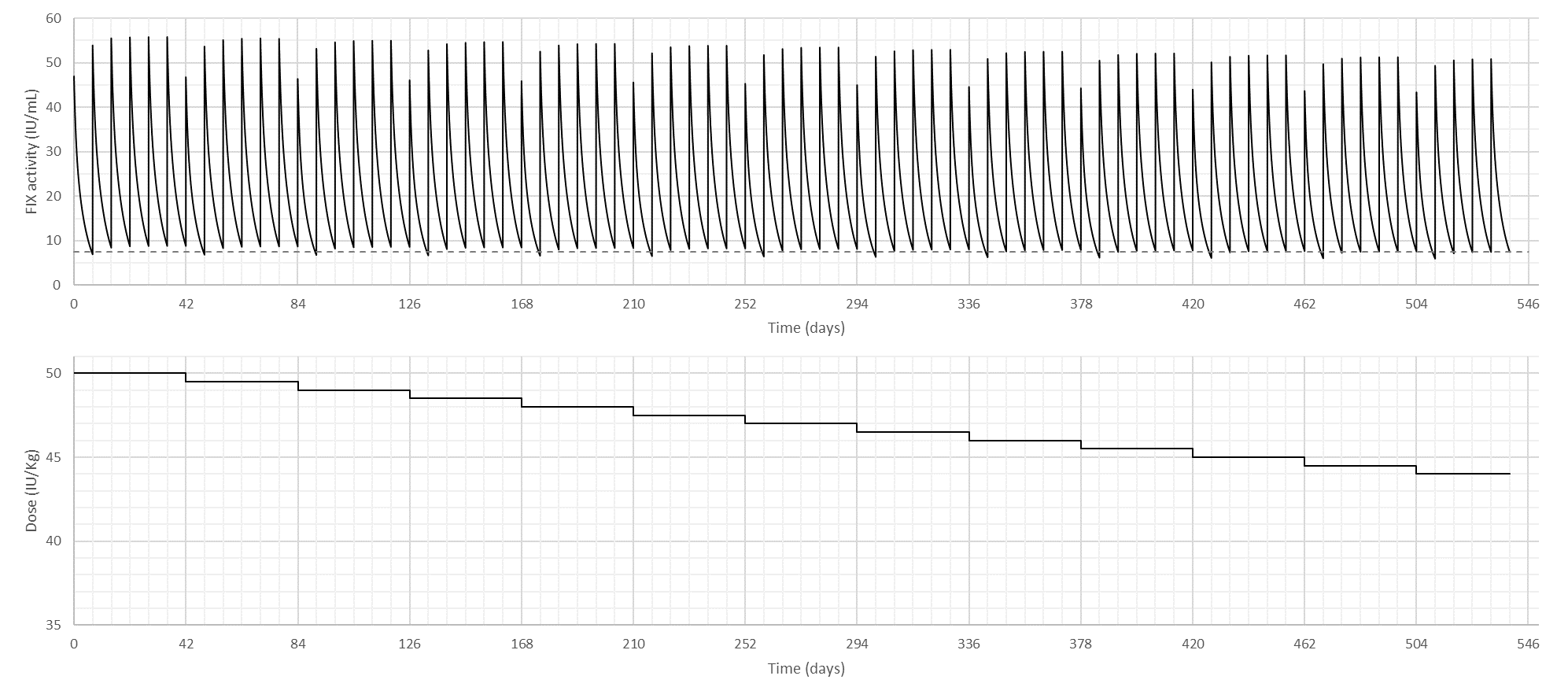


In this figure we show the simulated FIX activity level and weekly dose over time during the optimal rIX-FP posology search for a specific patient in the 6–11 years age range and a target level of 7.5 IU/mL. For these patients’ injection intervals were fixed at 7 days. The weekly dose started at 50.0 IU/Kg and was reduced by 0.5 IU/Kg while steady-state trough level was above 7.5 IU/mL. The last simulated dose was 44.0 IU/Kg (from simulation day 504 onwards), for which the simulated steady-state trough level was 7.479 IU/mL (simulation day 539), below the target level. Therefore, optimal posology was set as the penultimate simulated posology, which considered a weekly dose of 44.5 IU/Kg and for which the steady-state trough level was estimated to be 7.592 IU/mL.

***Supplementary References***

1. Cochrane collaboration. 7. Selecting studies and collection data, in Cochrane Handbook for Systematic Reviews of Interventions, J.P. Higgins and S. Green, Editors. March 2011. 2011.

2. Collins PW, Moss J, Knobe K, Groth A, Colberg T, Watson E. Population pharmacokinetic modeling for dose setting of nonacog beta pegol (N9-GP), a glycoPEGylated recombinant factor IX. J Thromb Haemost. 2012;10(11):2305-12.

3. Zhang Y, Roberts J, Bensen-Kennedy D, et al. Population pharmacokinetics of a new long-acting recombinant coagulation factor IX albumin fusion protein for patients with severe hemophilia B. J Thromb Haemost. 2016;14(11):2132-40.

4. Suzuki A, Tomono Y, Korth-Bradley JM. Population pharmacokinetic modelling of factor IX activity after administration of recombinant factor IX in patients with haemophilia B. Haemophilia. 2016;22(5):e359-66.

5. Bjorkman S. Population pharmacokinetics of recombinant factor IX: implications for dose tailoring. Haemophilia. 2013;19(5):753-7.

6. Diao L, Li S, Ludden T, Gobburu J, Nestorov I, Jiang H. Population pharmacokinetic modelling of recombinant factor IX Fc fusion protein (rFIXFc) in patients with haemophilia B. Clin Pharmacokinet. 2014;53(5):467-77.

**Supplementary Results**

***Infants – Ideal posology***

**Weight**

Mean (SD) 17.0 (5.7)

| **Patients below 6 \| Target 1%** |  |  |  |  |  |  |
| --- | --- | --- | --- | --- | --- | --- |
|  | **rIX-FP** | | **rFIX** | | **rFIXFc** | |
| **Steady-state FIX trough levels** Median (IQR) | 4.2 | | 1.0 | | 1.7 | |
|  | (2.8–6.1) | | (1.0–1.0) | | (1.4–2.1) | |
| Mean (SD) | 4.7 | | 0.9 | | 1.8 | |
|  | (2.6) | | (0.2) | | (0.6) | |
| **Patients below target, %** | 0.3% | | 25.0% | | 0.0% | |
| **Dose (IU/kg per week)** Median (IQR) | 35.0 | | 165.0 | | 50.0 | |
|  | (35.0–35.0) | | (132.1–238.0) | | (50.0–50.0) | |
| Mean (SD) | 35.2 | | 170.3 | | 50.6 | |
|  | (1.3) | | (51.2) | | (3.2) | |
| **Patient among dose-intervals, %** | 7d | 100.0% | 3d | 45.0% | 7d | 100.0% |
|  |  |  | 3.5d | 18.3% |  |  |
|  |  |  | 4d | 23.5% |  |  |
|  |  |  | 5d | 9.0% |  |  |
|  |  |  | 6d | 3.0% |  |  |
|  |  |  | 7d | 1.2% |  |  |
| **Ratio between weekly dose (IU/kg) FIX product and rIX-FP** Median (IQR) |  |  | 4.7 | | 1.4 | |
|  |  |  | (3.8–6.7) | | (1.4–1.4) | |
| Mean (SD) |  | | 4.8 | | 1.4 | |
|  |  | | (1.5) | | (0.1) | |

| **Patients below 6 \| Target 2%** |  |  |  |  |  |  |
| --- | --- | --- | --- | --- | --- | --- |
|  | **rIX-FP** | | **rFIX** | | **rFIXFc** | |
| **Steady-state FIX trough levels** Median (IQR) | 4.2 | | 1.9 | | 2.0 | |
|  | (2.8–6.1) | | (1.0–2.0) | | (2.0–2.1) | |
| Mean (SD) | 4.8 | | 1.5 | | 2.2 | |
|  | (2.6) | | (0.6) | | (0.4) | |
| **Patients below target, %** | 3.2% | | 52.3% | | 59.0% | |
| **Dose (IU/kg per week)** Median (IQR) | 35.0 | | 238.0 | | 58.0 | |
|  | (35.0–35.0) | | (171.5–238.0) | | (50.0–73.5) | |
| Mean (SD) | 36.1 | | 205.1 | | 63.9 | |
|  | (3.3) | | (41.8) | | (15.7) | |
| **Patient among dose-intervals, %** | 7d | 100.0% | 3d | 72.8% | 7d | 100.0% |
|  |  |  | 3.5d | 13.0% |  |  |
|  |  |  | 4d | 10.8% |  |  |
|  |  |  | 5d | 2.9% |  |  |
|  |  |  | 6d | 0.5% |  |  |
|  |  |  | 7d | 0.2% |  |  |
| **Ratio between weekly dose (IU/kg) FIX product and rIX-FP** Median (IQR) |  |  | 6.3 | | 1.6 | |
|  |  |  | (4.8–6.8) | | (1.4–2.0) | |
| Mean (SD) |  | | 5.7 | | 1.8 | |
|  |  | | (1.2) | | (0.4) | |

| **Patients below 6 \| Target 3%** |  |  |  |  |  |  |
| --- | --- | --- | --- | --- | --- | --- |
|  | **rIX-FP** | | **rFIX** | | **rFIXFc** | |
| **Steady-state FIX trough levels** Median (IQR) | 4.2 | | 1.9 | | 3.0 | |
|  | (3.0–6.1) | | (1.0–3.0) | | (2.7–3.0) | |
| Mean (SD) | 4.9 | | 1.9 | | 2.8 | |
|  | (2.4) | | (1.0) | | (0.4) | |
| **Patients below target, %** | 9.7% | | 71.0% | | 33.6% | |
| **Dose (IU/kg per week)** Median (IQR) | 35.0 | | 238.0 | | 87.0 | |
|  | (35.0–37.0) | | (218.2–238.0) | | (70.0–100.0) | |
| Mean (SD) | 37.6 | | 221.2 | | 83.4 | |
|  | (5.1) | | (31.2) | | (16.6) | |
| **Patient among dose-intervals, %** | 7d | 100.0% | 3d | 86.3% | 7d | 100.0% |
|  |  |  | 3.5d | 7.4% |  |  |
|  |  |  | 4d | 5.3% |  |  |
|  |  |  | 5d | 0.8% |  |  |
|  |  |  | 6d | 0.2% |  |  |
|  |  |  | 7d | 0.0% |  |  |
| **Ratio between weekly dose (IU/kg) FIX product and rIX-FP** Median (IQR) |  |  | 6.8 | | 2.2 | |
|  |  |  | (5.0–6.8) | | (1.9–2.8) | |
| Mean (SD) |  | | 6.0 | | 2.2 | |
|  |  | | (1.1) | | (0.5) | |

| **Patients below 6 \| Target 5%** |  |  |  |  |  |  |
| --- | --- | --- | --- | --- | --- | --- |
|  | **rIX-FP** | | **rFIX** | | **rFIXFc** | |
| **Steady-state FIX trough levels** Median (IQR) | 5.1 | | 1.9 | | 3.5 | |
|  | (4.6–6.1) | | (1.0–3.3) | | (2.7–4.3) | |
| Mean (SD) | 5.5 | | 2.2 | | 3.5 | |
|  | (2.2) | | (1.5) | | (1.0) | |
| **Patients below target, %** | 28.6% | | 89.2% | | 87.3% | |
| **Dose (IU/kg per week)** Median (IQR) | 40.0 | | 238.0 | | 100.0 | |
|  | (35.0–50.0) | | (238.0–238.0) | | (100.0–100.0) | |
| Mean (SD) | 41.6 | | 233.2 | | 98.3 | |
|  | (6.6) | | (16.4) | | (5.7) | |
| **Patient among dose-intervals, %** | 7d | 100.0% | 3d | 96.2% | 7d | 100.0% |
|  |  |  | 3.5d | 2.7% |  |  |
|  |  |  | 4d | 1.0% |  |  |
|  |  |  | 5d | 0.1% |  |  |
|  |  |  | 6d | 0.0% |  |  |
|  |  |  | 7d | 0.0% |  |  |
| **Ratio between weekly dose (IU/kg) FIX product and rIX-FP** Median (IQR) |  |  | 5.7 | | 2.4 | |
|  |  |  | (4.8–6.8) | | (2.0–2.9) | |
| Mean (SD) |  | | 5.7 | | 2.4 | |
|  |  | | (1.0) | | (0.4) | |

| **Patients below 6 \| Target 7.5%** |  |  |  |  |  |  |
| --- | --- | --- | --- | --- | --- | --- |
|  | **rIX-FP** | | **rFIX** | | **rFIXFc** | |
| **Steady-state FIX trough levels** Median (IQR) | 6.8 | | 1.9 | | 3.5 | |
|  | (4.6–7.6) | | (1.0–3.3) | | (2.7–4.3) | |
| Mean (SD) | 6.3 | | 2.4 | | 3.6 | |
|  | (2.3) | | (1.8) | | (1.2) | |
| **Patients below target, %** | 56.9% | | 96.9% | | 99.4% | |
| **Dose (IU/kg per week)** Median (IQR) | 50.0 | | 238.0 | | 100.0 | |
|  | (41.5–50.0) | | (238.0–238.0) | | (100.0–100.0) | |
| Mean (SD) | 45.9 | | 236.9 | | 99.9 | |
|  | (5.8) | | (7.2) | | (0.9) | |
| **Patient among dose-intervals, %** | 7d | 100.0% | 3d | 99.3% | 7d | 100.0% |
|  |  |  | 3.5d | 0.4% |  |  |
|  |  |  | 4d | 0.2% |  |  |
|  |  |  | 5d | 0.0% |  |  |
|  |  |  | 6d | 0.0% |  |  |
|  |  |  | 7d | 0.0% |  |  |
| **Ratio between weekly dose (IU/kg) FIX product and rIX-FP** Median (IQR) |  |  | 4.8 | | 2.0 | |
|  |  |  | (4.8–5.7) | | (2.0–2.4) | |
| Mean (SD) |  | | 5.3 | | 2.2 | |
|  |  | | (0.8) | | (0.3) | |

| **Patients below 6 \| Target 10%** |  |  |  |  |  |  |
| --- | --- | --- | --- | --- | --- | --- |
|  | **rIX-FP** | | **rFIX** | | **rFIXFc** | |
| **Steady-state FIX trough levels** Median (IQR) | 6.8 | | 1.9 | | 3.5 | |
|  | (4.6–9.6) | | (1.0–3.3) | | (2.7–4.3) | |
| Mean (SD) | 6.9 | | 2.4 | | 3.6 | |
|  | (2.8) | | (2.0) | | (1.2) | |
| **Patients below target, %** | 77.3% | | 99.2% | | 100.0% | |
| **Dose (IU/kg per week)** Median (IQR) | 50.0 | | 238.0 | | 100.0 | |
|  | (50.0–50.0) | | (238.0–238.0) | | (100.0–100.0) | |
| Mean (SD) | 48.1 | | 237.8 | | 100 | |
|  | (4.2) | | (3.1) | | (0.3) | |
| **Patient among dose-intervals, %** | 7d | 100.0% | 3d | 99.8% | 7d | 100.0% |
|  |  |  | 3.5d | 0.2% |  |  |
|  |  |  | 4d | 0.0% |  |  |
|  |  |  | 5d | 0.0% |  |  |
|  |  |  | 6d | 0.0% |  |  |
|  |  |  | 7d | 0.0% |  |  |
| **Ratio between weekly dose (IU/kg) FIX product and rIX-FP** Median (IQR) |  |  | 4.8 | | 2.0 | |
|  |  |  | (4.8–4.8) | | (2.0–2.0) | |
| Mean (SD) |  | | 5.0 | | 2.1 | |
|  |  | | (0.5) | | (0.2) | |

| **Patients below 6 \| Target 15%** |  |  |  |  |  |  |
| --- | --- | --- | --- | --- | --- | --- |
|  | **rIX-FP** | | **rFIX** | | **rFIXFc** | |
| **Steady-state FIX trough levels** Median (IQR) | 6.8 | | 1.9 | | 3.5 | |
|  | (4.6–9.6) | | (1.0–3.3) | | (2.7–4.3) | |
| Mean (SD) | 7.4 | | 2.4 | | 3.6 | |
|  | (3.6) | | (2.0) | | (1.2) | |
| **Patients below target, %** | 94.8% | | 99.9% | | 100.0% | |
| **Dose (IU/kg per week)** Median (IQR) | 50.0 | | 238.0 | | 100.0 | |
|  | (50.0–50.0) | | (238.0–238.0) | | (100.0–100.0) | |
| Mean (SD) | 49.7 | | 238.0 | | 100.0 | |
|  | (1.6) | | (0.6) | | (0.0) | |
| **Patient among dose-intervals, %** | 7d | 100.0% | 3d | 100.0% | 7d | 100.0% |
|  |  |  | 3.5d | 0.0% |  |  |
|  |  |  | 4d | 0.0% |  |  |
|  |  |  | 5d | 0.0% |  |  |
|  |  |  | 6d | 0.0% |  |  |
|  |  |  | 7d | 0.0% |  |  |
| **Ratio between weekly dose (IU/kg) FIX product and rIX-FP** Median (IQR) |  |  | 4.8 | | 2.0 | |
|  |  |  | (4.8–4.8) | | (2.0–2.0) | |
| Mean (SD) |  | | 4.8 | | 2.0 | |
|  |  | | (0.2) | | (0.1) | |

**Children – Ideal posology**

**Weight**

Mean (SD) 34.9 (10.2)

| **Patients 6 to 11 \| Target 1%** |  |  |  |  |  |  |
| --- | --- | --- | --- | --- | --- | --- |
|  | **rIX-FP** | | **rFIX** | | **rFIXFc** | |
| **Steady-state FIX trough levels** Median (IQR) | 7.1 | | 1.0 | | 2.1 | |
|  | (5.0–9.8) | | (1.0–1.0) | | (1.7–2.7) | |
| Mean (SD) | 7.7 | | 0.9 | | 2.3 | |
|  | (3.7) | | (0.2) | | (0.8) | |
| **Patients below target, %** | 0.0% | | 21.7% | | 0.0% | |
| **Dose (IU/kg per week)** Median (IQR) | 35.0 | | 123.4 | | 50.0 | |
|  | (35.0–35.0) | | (89.2–164.5) | | (50.0–50.0) | |
| Mean (SD) | 35 | | 122.1 | | 50.1 | |
|  | (0.3) | | (45.8) | | (1.3) | |
| **Patient among dose-intervals, %** | 7d | 100.0% | 3d | 40.2% | 7d | 100.0% |
|  |  |  | 3.5d | 18.6% |  |  |
|  |  |  | 4d | 41.1% |  |  |
| **Ratio between weekly dose (IU/kg) FIX product and rIX-FP** Median (IQR) |  |  | 3.5 | | 1.4 | |
|  |  |  | (2.5–4.7) | | (1.4–1.4) | |
| Mean (SD) |  | | 3.5 | | 1.4 | |
|  |  | | (1.3) | | (0.0) | |
| **Patients 6 to 11 \| Target 2%** |  |  |  |  |  |  |
|  | **rIX-FP** | | **rFIX** | | **rFIXFc** | |
| **Steady-state FIX trough levels** Median (IQR) | 7.1 | | 2.0 | | 2.1 | |
|  | (5.0–9.8) | | (1.1–2.0) | | (2.0–2.7) | |
| Mean (SD) | 7.8 | | 1.6 | | 2.4 | |
|  | (3.7) | | (0.6) | | (0.6) | |
| **Patients below target, %** | 0.3% | | 49.8% | | 1.8% | |
| **Dose (IU/kg per week)** Median (IQR) | 35.0 | | 182.0 | | 50.0 | |
|  | (35.0–35.0) | | (130.7–182.0) | | (50.0–59.5) | |
| Mean (SD) | 35.1 | | 154.7 | | 56.9 | |
|  | (1.1) | | (34.6) | | (12.0) | |
| **Patient among dose-intervals, %** | 7d | 100.0% | 3d | 69.8% | 7d | 100.0% |
|  |  |  | 3.5d | 14.1% |  |  |
|  |  |  | 4d | 16.2% |  |  |
| **Ratio between weekly dose (IU/kg) FIX product and rIX-FP** Median (IQR) |  |  | 5.1 | | 1.4 | |
|  |  |  | (3.7–5.2) | | (1.4–1.7) | |
| Mean (SD) |  | | 4.4 | | 1.6 | |
|  |  | | (1.0) | | (0.3) | |

| **Patients 6 to 11 \| Target 3%** |  |  |  | |  | |  | |  | |
| --- | --- | --- | --- | --- | --- | --- | --- | --- | --- | --- |
|  | **rIX-FP** | | **rFIX** | | | | **rFIXFc** | | | |
| **Steady-state FIX trough levels** Median (IQR) | 7.1 | | 2.0 | | | | 3.0 | | | |
|  | (5.0–9.8) | | (1.1–3.0) | | | | (3.0–3.0) | | | |
| Mean (SD) | 7.8 | | 2.0 | | | | 3.0 | | | |
|  | (3.7) | | (1.0) | | | | (0.4) | | | |
| **Patients below target, %** | 1.2% | | 69.5% | | | | 15.7% | | | |
| **Dose (IU/kg per week)** Median (IQR) | 35.0 | | 182.0 | | | | 70.5 | | | |
|  | (35.0–35.0) | | (162.2–182.0) | | | | (56.0–89.0) | | | |
| Mean (SD) | 35.5 | | 168.6 | | | | 72.8 | | | |
|  | (2.2) | | (24.7) | | | | (17.9) | | | |
| **Patient among dose-intervals, %** | 7d | 100.0% | 3d | | 85.3% | | 7d | | 100.0% | |
|  |  |  | 3.5d | | 8.1% | |  | |  | |
|  |  |  | 4d | | 6.6% | |  | |  | |
| **Ratio between weekly dose (IU/kg) FIX product and rIX-FP** Median (IQR) |  |  | 5.2 | | | | 2.0 | | | |
|  |  |  | (4.4–5.2) | | | | (1.6–2.5) | | | |
| Mean (SD) |  | | 4.8 | | | | 2.1 | | | |
|  |  | | (0.7) | | | | (0.5) | | | |
| **Patients 6 to 11 \| Target 5%** |  |  |  |  | |  | |  | |  |
|  | **rIX-FP** | | **rFIX** | | | **rFIXFc** | | | |  |
| **Steady-state FIX trough levels** Median (IQR) | 7.1 | | 2.0 | | | 4.3 | | | |  |
|  | (5.1–9.8) | | (1.1–3.4) | | | (3.4–5.0) | | | |  |
| Mean (SD) | 8.0 | | 2.3 | | | 4.1 | | | |  |
|  | (3.4) | | (1.5) | | | (0.9) | | | |  |
| **Patients below target, %** | 6.5% | | 89.6% | | | 67.5% | | | |  |
| **Dose (IU/kg per week)** Median (IQR) | 35.0 | | 182.0 | | | 100.0 | | | |  |
|  | (35.0–35.0) | | (182.0–182.0) | | | (93.0–100.0) | | | |  |
| Mean (SD) | 37.1 | | 178.5 | | | 94.1 | | | |  |
|  | (4.5) | | (12.2) | | | (11.2) | | | |  |
| **Patient among dose-intervals, %** | 7d | 100.0% | 3d | 96.4% | | 7d | | 100.0% | |  |
|  |  |  | 3.5d | 2.5% | |  | |  | |  |
|  |  |  | 4d | 1.1% | |  | |  | |  |
| **Ratio between weekly dose (IU/kg) FIX product and rIX-FP** Median (IQR) |  |  | 5.2 | | | 2.9 | | | |  |
|  |  |  | (4.7–5.2) | | | (2.3–2.9) | | | |  |
| Mean (SD) |  | | 4.9 | | | 2.6 | | | |  |
|  |  | | (0.6) | | | (0.4) | | | |  |

| **Patients 6 to 11 \| Target 7.5%** |  |  |  |  |  |  |
| --- | --- | --- | --- | --- | --- | --- |
|  | **rIX-FP** | | **rFIX** | | **rFIXFc** | |
| **Steady-state FIX trough levels** Median (IQR) | 7.6 | | 2.0 | | 4.3 | |
|  | (7.5–9.8) | | (1.1–3.4) | | (3.4–5.4) | |
| Mean (SD) | 8.7 | | 2.5 | | 4.5 | |
|  | (3.0) | | (1.8) | | (1.4) | |
| **Patients below target, %** | 21.2% | | 97.4% | | 95.1% | |
| **Dose (IU/kg per week)** Median (IQR) | 36.5 | | 182.0 | | 100.0 | |
|  | (35.0–47.5) | | (182.0–182.0) | | (100.0–100.0) | |
| Mean (SD) | 40.4 | | 181.3 | | 99.4 | |
|  | (6.3) | | (5.0) | | (3.2) | |
| **Patient among dose-intervals, %** | 7d | 100.0% | 3d | 99.2% | 7d | 100.0% |
|  |  |  | 3.5d | 0.6% |  |  |
|  |  |  | 4d | 0.2% |  |  |
| **Ratio between weekly dose (IU/kg) FIX product and rIX-FP** Median (IQR) |  |  | 4.9 | | 2.7 | |
|  |  |  | (3.8–5.2) | | (2.1–2.9) | |
| Mean (SD) |  | | 4.6 | | 2.5 | |
|  |  | | (0.7) | | (0.4) | |

| **Patients 6 to 11 \| Target 10%** |  |  |  |  |  |  |
| --- | --- | --- | --- | --- | --- | --- |
|  | **rIX-FP** | | **rFIX** | | **rFIXFc** | |
| **Steady-state FIX trough levels** Median (IQR) | 10.0 | | 2.0 | | 4.3 | |
|  | (8.1–10.1) | | (1.1–3.4) | | (3.4–5.4) | |
| Mean (SD) | 9.6 | | 2.5 | | 4.5 | |
|  | (3.0) | | (1.9) | | (1.6) | |
| **Patients below target, %** | 40.6% | | 99.2% | | 99.5% | |
| **Dose (IU/kg per week)** Median (IQR) | 46.0 | | 182.0 | | 100.0 | |
|  | (35.5–50.0) | | (182.0–182.0) | | (100.0–100.0) | |
| Mean (SD) | 43.7 | | 181.8 | | 100 | |
|  | (6.4) | | (2.2) | | (0.7) | |
| **Patient among dose-intervals, %** | 7d | 100.0% | 3d | 99.9% | 7d | 100.0% |
|  |  |  | 3.5d | 0.1% |  |  |
|  |  |  | 4d | 0.0% |  |  |
| **Ratio between weekly dose (IU/kg) FIX product and rIX-FP** Median (IQR) |  |  | 4.0 | | 2.2 | |
|  |  |  | (3.6–5.1) | | (2.0–2.8) | |
| Mean (SD) |  | | 4.3 | | 2.3 | |
|  |  | | (0.7) | | (0.4) | |

| **Patients 6 to 11 \| Target 15%** |  |  |  |  |  |  |
| --- | --- | --- | --- | --- | --- | --- |
|  | **rIX-FP** | | **rFIX** | | **rFIXFc** | |
| **Steady-state FIX trough levels** Median (IQR) | 11.3 | | 2.0 | | 4.3 | |
|  | (8.1–15.0) | | (1.1–3.4) | | (3.4–5.4) | |
| Mean (SD) | 11.1 | | 2.5 | | 4.5 | |
|  | (3.9) | | (2.0) | | (1.6) | |
| **Patients below target, %** | 73.6% | | 99.9% | | 100.0% | |
| **Dose (IU/kg per week)** Median (IQR) | 50.0 | | 182.0 | | 100.0 | |
|  | (49.5–50.0) | | (182.0–182.0) | | (100.0–100.0) | |
| Mean (SD) | 47.9 | | 182 | | 100 | |
|  | (4.3) | | (0.6) | | (0.0) | |
| **Patient among dose-intervals, %** | 7d | 100.0% | 3d | 100.0% | 7d | 100.0% |
|  |  |  | 3.5d | 0.0% |  |  |
|  |  |  | 4d | 0.0% |  |  |
| **Ratio between weekly dose (IU/kg) FIX product and rIX-FP** Median (IQR) |  |  | 3.6 | | 2.0 | |
|  |  |  | (3.6–3.7) | | (2.0–2.0) | |
| Mean (SD) |  | | 3.8 | | 2.1 | |
|  |  | | (0.4) | | (0.2) | |

***Adolescents – Ideal posology***

| **Weight** |  |  | |  |  |  |  |  |  |  |  |  |  |  |
| --- | --- | --- | --- | --- | --- | --- | --- | --- | --- | --- | --- | --- | --- | --- |
| Mean (SD) | 60.2 (14.0) | | |  |  |  |  |  |  |  |  |  |  |  |
| **Patients 12 to 17 \| Target 1%; Control 5%** |  | |  | |  |  |  |  |  |  |  |  |  |  |
|  | **rIX-FP** | | | | **rFIX** | | **rFIXFc** weekly | | **rFIXFc** ind. Interval | | **rFIXFc^Ⱡ^** weekly and ind.interval | | **N9-GP** | |
| **Steady-state FIX trough levels** Median (IQR) | 4.4 | | | | 1.0 | | 1.0 | | 1.3 | | 1.0 | | 4.1 | |
|  | (3.4–5.9) | | | | (1.0–1.0) | | (1.0–1.1) | | (1.2–1.8) | | (1.0–1.3) | | (3.3–5.1) | |
| Mean (SD) | 5.0 | | | | 1.0 | | 1.1 | | 1.6 | | 1.2 | | 4.3 | |
|  | (2.4) | | | | (0.2) | | (0.2) | | (0.6) | | (0.4) | | (1.3) | |
| **Patients below target, %** | 0.0% | | | | 13.6% | | 0.1% | | 0.3% | | 0.1% | | 0.0% | |
| **Dose (IU/kg per week)** Median (IQR) | 25.0 | | | | 112.0 | | 20.0 | | 53.8 | | 25.0 | | 10.0 | |
|  | (25.0–25.0) | | | | (68.2–140.0) | | (17.0–26.0) | | (53.8–58.3) | | (18.0–53.8) | | (10.0–10.0) | |
| Mean (SD) | 26.8 | | | | 108.4 | | 23 | | 57.9 | | 33.9 | | 10 | |
|  | (3.9) | | | | (46.6) | | (8.1) | | (7.4) | | (18.0) | | (0.0) | |
| **Patients among dose-intervals, %** | 7d | | 7.7% | | 3d | 29.1% | 7d | 100.0% | 8d | 1.7% | 7d | 68.8% | 7d | 100.0% |
|  | 10d | | 10.4% | | 3.5d | 17.6% |  |  | 9d | 3.6% | 8d | 0.5% |  |  |
|  | 14d | | 81.9% | | 4d | 53.3% |  |  | 10d | 7.0% | 9d | 1.2% |  |  |
|  |  | |  | |  |  |  |  | 11d | 10.4% | 10d | 2.3% |  |  |
|  |  | |  | |  |  |  |  | 12d | 12.7% | 11d | 3.3% |  |  |
|  |  | |  | |  |  |  |  | 13d | 60.6% | 12d | 4.0% |  |  |
|  |  | |  | |  |  |  |  | 14d | 3.9% | 13d | 18.7% |  |  |
|  |  | |  | |  |  |  |  | 15d | 0.0% | 14d | 1.2% |  |  |
|  |  | |  | |  |  |  |  | 16d | 0.0% | 15d | 0.0% |  |  |
|  |  | |  | |  |  |  |  | 17d | 0.0% | 16d | 0.0% |  |  |
|  |  | |  | |  |  |  |  | 18d | 0.0% | 17d | 0.0% |  |  |
|  |  | |  | |  |  |  |  | 19d | 0.0% | 18d | 0.0% |  |  |
|  |  | |  | |  |  |  |  | 20d | 0.0% | 19d | 0.0% |  |  |
|  |  | |  | |  |  |  |  | 21d | 0.0% | 20d | 0.0% |  |  |
|  |  | |  | |  |  |  |  |  |  | 21d | 0.0% |  |  |
| **Ratio between weekly dose (IU/kg) FIX product and rIX-FP** Median (IQR) |  | | | | 4.2 | | 0.8 | | 2.2 | | 1.0 | | 0.4 | |
|  |  | | | | (2.6–5.3) | | (0.7–1.0) | | (2.2–2.3) | | (0.7–2.2) | | (0.4–0.4) | |
| Mean (SD) |  | | | | 4.1 | | 0.9 | | 2.2 | | 1.3 | | 0.4 | |
|  |  | | | | (1.8) | | (0.3) | | (0.4) | | (0.7) | | (0.0) | |

^Ⱡ^Patients were randomly assigned to individualized interval prophylaxis from a Bernoulli distribution with a probability 0.315, based on data from clinical trial 998HB102 (NCT01027364). In the simulations underlying the results of this table, 68.8% of the simulated patients were assigned to receive weekly prophylaxis and 31.3% to individualized interval prophylaxis.

| **Patients 12 to 17 \| Target 1%; Control 7.5%** | | |  | |  | | |  | |  | | |  | | |  | | | |  | | |  | | | |  | | |  | | | |  | | |  |  |  |
| --- | --- | --- | --- | --- | --- | --- | --- | --- | --- | --- | --- | --- | --- | --- | --- | --- | --- | --- | --- | --- | --- | --- | --- | --- | --- | --- | --- | --- | --- | --- | --- | --- | --- | --- | --- | --- | --- | --- | --- |
|  | **rIX-FP** | | | | **rFIX** | | | | | **rFIXFc** weekly | | | | | | **rFIXFc** ind. Interval | | | | | | | **rFIXFc^Ⱡ^** weekly and ind.interval | | | | | | | **N9-GP** | | | | | | |  |  |  |
| **Steady-state FIX trough levels** Median (IQR) | 6.0 | | | | 1.0 | | | | | 1.0 | | | | | | 1.3 | | | | | | | 1.0 | | | | | | | 4.1 | | | | | | |  |  |  |
|  | (4.7–7.0) | | | | (1.0–1.0) | | | | | (1.0–1.1) | | | | | | (1.2–1.8) | | | | | | | (1.0–1.3) | | | | | | | (3.3–5.1) | | | | | | |  |  |  |
| Mean (SD) | 6.1 | | | | 1.0 | | | | | 1.1 | | | | | | 1.6 | | | | | | | 1.2 | | | | | | | 4.3 | | | | | | |  |  |  |
|  | (2.1) | | | | (0.2) | | | | | (0.2) | | | | | | (0.6) | | | | | | | (0.4) | | | | | | | (1.3) | | | | | | |  |  |  |
| **Patients below target, %** | 0.0% | | | | 13.6% | | | | | 0.0% | | | | | | 0.3% | | | | | | | 0.1% | | | | | | | 0.0% | | | | | | |  |  |  |
| **Dose (IU/kg per week)** Median (IQR) | 25.0 | | | | 112.0 | | | | | 20.0 | | | | | | 53.8 | | | | | | | 25.0 | | | | | | | 10.0 | | | | | | |  |  |  |
|  | (25.0–35.0) | | | | (68.2–140.0) | | | | | (17.0–26.0) | | | | | | (53.8–58.3) | | | | | | | (18.0–53.8) | | | | | | | (10.0–10.0) | | | | | | |  |  |  |
| Mean (SD) | 29.2 | | | | 108.4 | | | | | 23 | | | | | | 58 | | | | | | | 34 | | | | | | | 10 | | | | | | |  |  |  |
|  | -4.9 | | | | -46.6 | | | | | -8.1 | | | | | | -7.3 | | | | | | | -18.1 | | | | | | | 0 | | | | | | |  |  |  |
| **Patients among dose-intervals, %** | 7d | | 25.0% | | 3d | | | 29.1% | | 7d | | | 100.0% | | | 8d | | | | 1.7% | | | 7d | | | | 68.8% | | | 7d | | | | 100.0% | | |  |  |  |
|  | 10d | | 17.3% | | 3.5d | | | 17.6% | |  | | |  | | | 9d | | | | 3.6% | | | 8d | | | | 0.5% | | |  | | | |  | | |  |  |  |
|  | 14d | | 57.7% | | 4d | | | 53.3% | |  | | |  | | | 10d | | | | 7.0% | | | 9d | | | | 1.2% | | |  | | | |  | | |  |  |  |
|  |  | |  | |  | | |  | |  | | |  | | | 11d | | | | 10.4% | | | 10d | | | | 2.3% | | |  | | | |  | | |  |  |  |
|  |  | |  | |  | | |  | |  | | |  | | | 12d | | | | 12.7% | | | 11d | | | | 3.3% | | |  | | | |  | | |  |  |  |
|  |  | |  | |  | | |  | |  | | |  | | | 13d | | | | 64.3% | | | 12d | | | | 4.0% | | |  | | | |  | | |  |  |  |
|  |  | |  | |  | | |  | |  | | |  | | | 14d | | | | 0.2% | | | 13d | | | | 19.8% | | |  | | | |  | | |  |  |  |
|  |  | |  | |  | | |  | |  | | |  | | | 15d | | | | 0.0% | | | 14d | | | | 0.1% | | |  | | | |  | | |  |  |  |
|  |  | |  | |  | | |  | |  | | |  | | | 16d | | | | 0.0% | | | 15d | | | | 0.0% | | |  | | | |  | | |  |  |  |
|  |  | |  | |  | | |  | |  | | |  | | | 17d | | | | 0.0% | | | 16d | | | | 0.0% | | |  | | | |  | | |  |  |  |
|  |  | |  | |  | | |  | |  | | |  | | | 18d | | | | 0.0% | | | 17d | | | | 0.0% | | |  | | | |  | | |  |  |  |
|  |  | |  | |  | | |  | |  | | |  | | | 19d | | | | 0.0% | | | 18d | | | | 0.0% | | |  | | | |  | | |  |  |  |
|  |  | |  | |  | | |  | |  | | |  | | | 20d | | | | 0.0% | | | 19d | | | | 0.0% | | |  | | | |  | | |  |  |  |
|  |  | |  | |  | | |  | |  | | |  | | | 21d | | | | 0.0% | | | 20d | | | | 0.0% | | |  | | | |  | | |  |  |  |
|  |  | |  | |  | | |  | |  | | |  | | |  | | | |  | | | 21d | | | | 0.0% | | |  | | | |  | | |  |  |  |
| **Ratio between weekly dose (IU/kg) FIX product and rIX-FP** Median (IQR) |  | | | | 3.7 | | | | | 0.7 | | | | | | 2.2 | | | | | | | 0.9 | | | | | | | 0.4 | | | | | | |  |  |  |
|  |  | | | | (2.4–5.1) | | | | | (0.6–0.9) | | | | | | (1.5–2.2) | | | | | | | (0.7–1.6) | | | | | | | (0.3–0.4) | | | | | | |  |  |  |
| Mean (SD) |  | | | | 3.8 | | | | | 0.8 | | | | | | 2.0 | | | | | | | 1.2 | | | | | | | 0.4 | | | | | | |  |  |  |
|  |  | | | | (1.8) | | | | | (0.3) | | | | | | (0.4) | | | | | | | (0.7) | | | | | | | (0.1) | | | | | | |  |  |  |
| ^Ⱡ^Patients were randomly assigned to individualized interval prophylaxis from a Bernoulli distribution with a probability 0.315, based on data from clinical trial 998HB102 (NCT01027364). In the simulations underlying the results of this table, 68.8%of the simulated patients were assigned to receive weekly prophylaxis and 31.3% to individualized interval prophylaxis. | | | | | | | | | | | | | | | | | | | | | | | | | | | | | | | | | | | | |  |  |  |
| **Patients 12 to 17 \| Target 1%; Control 10%** | |  | |  | | |  | |  | | |  | | |  | | | |  | | |  | | | |  | | |  | | | |  | | |  | | | |
|  | | **rIX-FP** | | | | **rFIX** | | | | | **rFIXFc** weekly | | | | | | **rFIXFc** ind. Interval | | | | | | | **rFIXFc^Ⱡ^** weekly and ind.interval | | | | | | | **N9-GP** | | | | | | |  |  |
| **Steady-state FIX trough levels** Median (IQR) | | 7.7 | | | | 1.0 | | | | | 1.0 | | | | | | 1.3 | | | | | | | 1.0 | | | | | | | 4.1 | | | | | | |  |  |
|  |  | (6.0–9.0) | | | | (1.0–1.0) | | | | | (1.0–1.1) | | | | | | (1.2–1.8) | | | | | | | (1.0–1.3) | | | | | | | (3.3–5.1) | | | | | | |  |  |
| Mean (SD) | | 7.5 | | | | 1.0 | | | | | 1.1 | | | | | | 1.6 | | | | | | | 1.2 | | | | | | | 4.3 | | | | | | |  |  |
|  |  | (2.1) | | | | (0.2) | | | | | (0.2) | | | | | | (0.6) | | | | | | | (0.4) | | | | | | | (1.3) | | | | | | |  |  |
| **Patients below target, %** | | 0.0% | | | | 13.6% | | | | | 0.0% | | | | | | 0.3% | | | | | | | 0.1% | | | | | | | 0.0% | | | | | | |  |  |
| **Dose (IU/kg per week)** Median (IQR) | | 35.0 | | | | 112.0 | | | | | 20.0 | | | | | | 53.8 | | | | | | | 25.0 | | | | | | | 10.0 | | | | | | |  |  |
|  |  | (25.0–35.0) | | | | (68.2–140.0) | | | | | (17.0–26.0) | | | | | | (53.8–58.3) | | | | | | | (18.0–53.8) | | | | | | | (10.0–10.0) | | | | | | |  |  |
| Mean (SD) | | 31.4 | | | | 108.4 | | | | | 23 | | | | | | 58 | | | | | | | 34 | | | | | | | 10 | | | | | | |  |  |
|  |  | (4.8) | | | | (46.6) | | | | | (8.1) | | | | | | (7.3) | | | | | | | (18.1) | | | | | | | (0.0) | | | | | | |  |  |
| **Patients among dose-intervals, %** | | 7d | | 47.4% | | | 3d | | 29.1% | | | 7d | | | 100.0% | | | | 8d | | | 1.7% | | | | 7d | | | 68.8% | | | | 7d | | | 100.0% | | | |
|  |  | 10d | | 16.7% | | | 3.5d | | 17.6% | | |  | | |  | | | | 9d | | | 3.6% | | | | 8d | | | 0.5% | | | |  | | |  | | | |
|  |  | 14d | | 35.9% | | | 4d | | 53.3% | | |  | | |  | | | | 10d | | | 7.0% | | | | 9d | | | 1.2% | | | |  | | |  | | | |
|  |  |  | |  | | |  | |  | | |  | | |  | | | | 11d | | | 10.4% | | | | 10d | | | 2.3% | | | |  | | |  | | | |
|  |  |  | |  | | |  | |  | | |  | | |  | | | | 12d | | | 12.7% | | | | 11d | | | 3.3% | | | |  | | |  | | | |
|  |  |  | |  | | |  | |  | | |  | | |  | | | | 13d | | | 64.5% | | | | 12d | | | 4.0% | | | |  | | |  | | | |
|  |  |  | |  | | |  | |  | | |  | | |  | | | | 14d | | | 0.0% | | | | 13d | | | 19.9% | | | |  | | |  | | | |
|  |  |  | |  | | |  | |  | | |  | | |  | | | | 15d | | | 0.0% | | | | 14d | | | 0.0% | | | |  | | |  | | | |
|  |  |  | |  | | |  | |  | | |  | | |  | | | | 16d | | | 0.0% | | | | 15d | | | 0.0% | | | |  | | |  | | | |
|  |  |  | |  | | |  | |  | | |  | | |  | | | | 17d | | | 0.0% | | | | 16d | | | 0.0% | | | |  | | |  | | | |
|  |  |  | |  | | |  | |  | | |  | | |  | | | | 18d | | | 0.0% | | | | 17d | | | 0.0% | | | |  | | |  | | | |
|  |  |  | |  | | |  | |  | | |  | | |  | | | | 19d | | | 0.0% | | | | 18d | | | 0.0% | | | |  | | |  | | | |
|  |  |  | |  | | |  | |  | | |  | | |  | | | | 20d | | | 0.0% | | | | 19d | | | 0.0% | | | |  | | |  | | | |
|  |  |  | |  | | |  | |  | | |  | | |  | | | | 21d | | | 0.0% | | | | 20d | | | 0.0% | | | |  | | |  | | | |
|  |  |  | |  | | |  | |  | | |  | | |  | | | |  | | |  | | | | 21d | | | 0.0% | | | |  | | |  | | | |
| **Ratio between weekly dose (IU/kg) FIX product and rIX-FP** Median (IQR) | |  | | | | 3.5 | | | | | 0.7 | | | | | | 1.8 | | | | | | | 0.8 | | | | | | | 0.3 | | | | | | |  |  |
|  |  |  | | | | (2.2–4.8) | | | | | (0.5–0.9) | | | | | | (1.5–2.2) | | | | | | | (0.6–1.5) | | | | | | | (0.3–0.4) | | | | | | |  |  |
| Mean (SD) | |  | | | | 3.5 | | | | | 0.8 | | | | | | 1.9 | | | | | | | 1.1 | | | | | | | 0.3 | | | | | | |  |  |
|  |  |  | | | | (1.6) | | | | | (0.3) | | | | | | (0.4) | | | | | | | (0.6) | | | | | | | (0.1) | | | | | | |  |  |
| ^Ⱡ^Patients were randomly assigned to individualized interval prophylaxis from a Bernoulli distribution with a probability 0.315, based on data from clinical trial 998HB102 (NCT01027364). In the simulations underlying the results of this table, 68.8% of the simulated patients were assigned to receive weekly prophylaxis and 31.3% to individualized interval prophylaxis. | | | | | | | | | | | | | | | | | | | | | | | | | | | | | | | | | | | | | |  |  |
| **Patients 12 to 17 \| Target 1%; Control 15%** | |  | |  | | |  | |  | | |  | | |  | | | |  | | |  | | | |  | | |  | | | |  | | |  | | | |
|  | | **rIX-FP** | | | | **rFIX** | | | | | **rFIXFc** weekly | | | | | | **rFIXFc** ind. Interval | | | | | | | **rFIXFc^Ⱡ^** weekly and ind.interval | | | | | | | **N9-GP** | | | | | | |  |  |
| **Steady-state FIX trough levels** Median (IQR) | | 9.7 | | | | 1.0 | | | | | 1.0 | | | | | | 1.3 | | | | | | | 1.0 | | | | | | | 4.1 | | | | | | |  |  |
|  |  | (7.5–12.4) | | | | (1.0–1.0) | | | | | (1.0–1.1) | | | | | | (1.2–1.8) | | | | | | | (1.0–1.3) | | | | | | | (3.3–5.1) | | | | | | |  |  |
| Mean (SD) | | 9.8 | | | | 1.0 | | | | | 1.1 | | | | | | 1.6 | | | | | | | 1.2 | | | | | | | 4.3 | | | | | | |  |  |
|  |  | (3.1) | | | | (0.2) | | | | | (0.2) | | | | | | (0.6) | | | | | | | (0.4) | | | | | | | (1.3) | | | | | | |  |  |
| **Patients below target, %** | | 0.0% | | | | 13.6% | | | | | 0.0% | | | | | | 0.3% | | | | | | | 0.1% | | | | | | | 0.0% | | | | | | |  |  |
| **Dose (IU/kg per week)** Median (IQR) | | 35.0 | | | | 112.0 | | | | | 20.0 | | | | | | 53.8 | | | | | | | 25.0 | | | | | | | 10.0 | | | | | | |  |  |
|  |  | (35.0–35.0) | | | | (68.2–140.0) | | | | | (17.0–26.0) | | | | | | (53.8–58.3) | | | | | | | (18.0–53.8) | | | | | | | (10.0–10.0) | | | | | | |  |  |
| Mean (SD) | | 34 | | | | 108.4 | | | | | 23 | | | | | | 58 | | | | | | | 34 | | | | | | | 10 | | | | | | |  |  |
|  |  | (3.1) | | | | (46.6) | | | | | (8.1) | | | | | | (7.3) | | | | | | | (18.1) | | | | | | | (0.0) | | | | | | |  |  |
| **Patients among dose-intervals, %** | | 7d | | 81.0% | | | 3d | | 29.1% | | | 7d | | | 100.0% | | | | 8d | | | 1.7% | | | | 7d | | | 68.8% | | | | 7d | | | 100.0% | | | |
|  |  | 10d | | 8.6% | | | 3.5d | | 17.6% | | |  | | |  | | | | 9d | | | 3.6% | | | | 8d | | | 0.5% | | | |  | | |  | | | |
|  |  | 14d | | 10.4% | | | 4d | | 53.3% | | |  | | |  | | | | 10d | | | 7.0% | | | | 9d | | | 1.2% | | | |  | | |  | | | |
|  |  |  | |  | | |  | |  | | |  | | |  | | | | 11d | | | 10.4% | | | | 10d | | | 2.3% | | | |  | | |  | | | |
|  |  |  | |  | | |  | |  | | |  | | |  | | | | 12d | | | 12.7% | | | | 11d | | | 3.3% | | | |  | | |  | | | |
|  |  |  | |  | | |  | |  | | |  | | |  | | | | 13d | | | 64.5% | | | | 12d | | | 4.0% | | | |  | | |  | | | |
|  |  |  | |  | | |  | |  | | |  | | |  | | | | 14d | | | 0.0% | | | | 13d | | | 19.9% | | | |  | | |  | | | |
|  |  |  | |  | | |  | |  | | |  | | |  | | | | 15d | | | 0.0% | | | | 14d | | | 0.0% | | | |  | | |  | | | |
|  |  |  | |  | | |  | |  | | |  | | |  | | | | 16d | | | 0.0% | | | | 15d | | | 0.0% | | | |  | | |  | | | |
|  |  |  | |  | | |  | |  | | |  | | |  | | | | 17d | | | 0.0% | | | | 16d | | | 0.0% | | | |  | | |  | | | |
|  |  |  | |  | | |  | |  | | |  | | |  | | | | 18d | | | 0.0% | | | | 17d | | | 0.0% | | | |  | | |  | | | |
|  |  |  | |  | | |  | |  | | |  | | |  | | | | 19d | | | 0.0% | | | | 18d | | | 0.0% | | | |  | | |  | | | |
|  |  |  | |  | | |  | |  | | |  | | |  | | | | 20d | | | 0.0% | | | | 19d | | | 0.0% | | | |  | | |  | | | |
|  |  |  | |  | | |  | |  | | |  | | |  | | | | 21d | | | 0.0% | | | | 20d | | | 0.0% | | | |  | | |  | | | |
|  |  |  | |  | | |  | |  | | |  | | |  | | | |  | | |  | | | | 21d | | | 0.0% | | | |  | | |  | | | |
| **Ratio between weekly dose (IU/kg) FIX product and rIX-FP** Median (IQR) | |  | | | | 3.3 | | | | | 0.6 | | | | | | 1.5 | | | | | | | 0.8 | | | | | | | 0.3 | | | | | | |  |  |
|  |  |  | | | | (2.0–4.2) | | | | | (0.5–0.8) | | | | | | (1.5–1.8) | | | | | | | (0.5–1.5) | | | | | | | (0.3–0.3) | | | | | | |  |  |
| Mean (SD) | |  | | | | 3.2 | | | | | 0.7 | | | | | | 1.7 | | | | | | | 1.0 | | | | | | | 0.3 | | | | | | |  |  |
|  |  |  | | | | (1.4) | | | | | (0.3) | | | | | | (0.3) | | | | | | | (0.6) | | | | | | | (0.0) | | | | | | |  |  |
| ^Ⱡ^Patients were randomly assigned to individualized interval prophylaxis from a Bernoulli distribution with a probability 0.315, based on data from clinical trial 998HB102 (NCT01027364). In the simulations underlying the results of this table, 68.8% of the simulated patients were assigned to receive weekly prophylaxis and 31.3% to individualized interval prophylaxis. | | | | | | | | | | | | | | | | | | | | | | | | | | | | | | | | | | | | | |  |  |
| **Patients 12 to 17 \| Target 1%; Control 20%** | |  | |  | | |  | |  | | |  | |  | | | |  | | |  | | | |  | | |  | | | |  | | |  | | | |  |
|  | | **rIX-FP** | | | | | **rFIX** | | | | | **rFIXFc** weekly | | | | | | **rFIXFc** ind. Interval | | | | | | | **rFIXFc^Ⱡ^** weekly and ind.interval | | | | | | | **N9-GP** | | | | | | |  |
| **Steady-state FIX trough levels** Median (IQR) | | 10.3 | | | | | 1.0 | | | | | 1.0 | | | | | | 1.3 | | | | | | | 1.0 | | | | | | | 4.1 | | | | | | |  |
|  |  | (7.5–13.6) | | | | | (1.0–1.0) | | | | | (1.0–1.1) | | | | | | (1.2–1.8) | | | | | | | (1.0–1.3) | | | | | | | (3.3–5.1) | | | | | | |  |
| Mean (SD) | | 10.7 | | | | | 1.0 | | | | | 1.1 | | | | | | 1.6 | | | | | | | 1.2 | | | | | | | 4.3 | | | | | | |  |
|  |  | (4.1) | | | | | (0.2) | | | | | (0.2) | | | | | | (0.6) | | | | | | | (0.4) | | | | | | | (1.3) | | | | | | |  |
| **Patients below target, %** | | 0.0% | | | | | 13.6% | | | | | 0.0% | | | | | | 0.3% | | | | | | | 0.1% | | | | | | | 0.0% | | | | | | |  |
| **Dose (IU/kg per week)** Median (IQR) | | 35.0 | | | | | 112.0 | | | | | 20.0 | | | | | | 53.8 | | | | | | | 25.0 | | | | | | | 10.0 | | | | | | |  |
|  |  | (35.0–35.0) | | | | | (68.2–140.0) | | | | | (17.0–26.0) | | | | | | (53.8–58.3) | | | | | | | (18.0–53.8) | | | | | | | (10.0–10.0) | | | | | | |  |
| Mean (SD) | | 34.7 | | | | | 108.4 | | | | | 23 | | | | | | 58 | | | | | | | 34 | | | | | | | 10 | | | | | | |  |
|  |  | (1.6) | | | | | (46.6) | | | | | (8.1) | | | | | | (7.3) | | | | | | | (18.1) | | | | | | | (0.0) | | | | | | |  |
| **Patients among dose-intervals, %** | | 7d | | 94.7% | | | 3d | | 29.1% | | | 7d | | 100.0% | | | | 8d | | | 1.7% | | | | 7d | | | 68.8% | | | | 7d | | | 100.0% | | | |  |
|  |  | 10d | | 2.6% | | | 3.5d | | 17.6% | | |  | |  | | | | 9d | | | 3.6% | | | | 8d | | | 0.5% | | | |  | | |  | | | |  |
|  |  | 14d | | 2.7% | | | 4d | | 53.3% | | |  | |  | | | | 10d | | | 7.0% | | | | 9d | | | 1.2% | | | |  | | |  | | | |  |
|  |  |  | |  | | |  | |  | | |  | |  | | | | 11d | | | 10.4% | | | | 10d | | | 2.3% | | | |  | | |  | | | |  |
|  |  |  | |  | | |  | |  | | |  | |  | | | | 12d | | | 12.7% | | | | 11d | | | 3.3% | | | |  | | |  | | | |  |
|  |  |  | |  | | |  | |  | | |  | |  | | | | 13d | | | 64.5% | | | | 12d | | | 4.0% | | | |  | | |  | | | |  |
|  |  |  | |  | | |  | |  | | |  | |  | | | | 14d | | | 0.0% | | | | 13d | | | 19.9% | | | |  | | |  | | | |  |
|  |  |  | |  | | |  | |  | | |  | |  | | | | 15d | | | 0.0% | | | | 14d | | | 0.0% | | | |  | | |  | | | |  |
|  |  |  | |  | | |  | |  | | |  | |  | | | | 16d | | | 0.0% | | | | 15d | | | 0.0% | | | |  | | |  | | | |  |
|  |  |  | |  | | |  | |  | | |  | |  | | | | 17d | | | 0.0% | | | | 16d | | | 0.0% | | | |  | | |  | | | |  |
|  |  |  | |  | | |  | |  | | |  | |  | | | | 18d | | | 0.0% | | | | 17d | | | 0.0% | | | |  | | |  | | | |  |
|  |  |  | |  | | |  | |  | | |  | |  | | | | 19d | | | 0.0% | | | | 18d | | | 0.0% | | | |  | | |  | | | |  |
|  |  |  | |  | | |  | |  | | |  | |  | | | | 20d | | | 0.0% | | | | 19d | | | 0.0% | | | |  | | |  | | | |  |
|  |  |  | |  | | |  | |  | | |  | |  | | | | 21d | | | 0.0% | | | | 20d | | | 0.0% | | | |  | | |  | | | |  |
|  |  |  | |  | | |  | |  | | |  | |  | | | |  | | |  | | | | 21d | | | 0.0% | | | |  | | |  | | | |  |
| **Ratio between weekly dose (IU/kg) FIX product and rIX-FP** Median (IQR) | |  | | | | | 3.2 | | | | | 0.6 | | | | | | 1.5 | | | | | | | 0.7 | | | | | | | 0.3 | | | | | | |  |
|  |  |  | | | | | (2.0–4.0) | | | | | (0.5–0.8) | | | | | | (1.5–1.7) | | | | | | | (0.5–1.5) | | | | | | | (0.3–0.3) | | | | | | |  |
| Mean (SD) | |  | | | | | 3.1 | | | | | 0.7 | | | | | | 1.7 | | | | | | | 1.0 | | | | | | | 0.3 | | | | | | |  |
|  |  |  | | | | | (1.4) | | | | | (0.2) | | | | | | (0.2) | | | | | | | (0.5) | | | | | | | (0.0) | | | | | | |  |

^Ⱡ^Patients were randomly assigned to individualized interval prophylaxis from a Bernoulli distribution with a probability 0.315, based on data from clinical trial 998HB102 (NCT01027364). In the simulations underlying the results of this table, 68.8% of the simulated patients were assigned to receive weekly prophylaxis and 31.3% to individualized interval prophylaxis.

| **Patients 12 to 17 \| Target 2%; Control 5%** | |  | |  | |  | |  | |  | | |  | | |  | | |  | | |  | | |  | | |  | | |  | | |
| --- | --- | --- | --- | --- | --- | --- | --- | --- | --- | --- | --- | --- | --- | --- | --- | --- | --- | --- | --- | --- | --- | --- | --- | --- | --- | --- | --- | --- | --- | --- | --- | --- | --- |
|  | | **rIX-FP** | | | | **rFIX** | | | | **rFIXFc** weekly | | | | | | **rFIXFc** ind. Interval | | | | | | **rFIXFc^Ⱡ^** weekly and ind.interval | | | | | | **N9-GP** | | | | | |
| **Steady-state FIX trough levels** Median (IQR) | | 4.4 | | | | 2.0 | | | | 2.0 | | | | | | 2.2 | | | | | | 2.0 | | | | | | 4.1 | | | | | |
|  |  | (3.4–5.9) | | | | (1.5–2.0) | | | | (2.0–2.0) | | | | | | (2.1–2.4) | | | | | | (2.0–2.1) | | | | | | (3.3–5.1) | | | | | |
| Mean (SD) | | 5.0 | | | | 1.7 | | | | 2.0 | | | | | | 2.3 | | | | | | 2.1 | | | | | | 4.3 | | | | | |
|  |  | (2.4) | | | | (0.5) | | | | (0.1) | | | | | | (0.3) | | | | | | (0.2) | | | | | | (1.3) | | | | | |
| **Patients below target, %** | | 0.0% | | | | 37.8% | | | | 2.7% | | | | | | 6.3% | | | | | | 3.8% | | | | | | 0.0% | | | | | |
| **Dose (IU/kg per week)** Median (IQR) | | 25.0 | | | | 149.0 | | | | 40.0 | | | | | | 70.0 | | | | | | 50.0 | | | | | | 10.0 | | | | | |
|  |  | (25.0–25.0) | | | | (120.8–182.0) | | | | (32.0–52.0) | | | | | | (58.3–77.8) | | | | | | (35.5–63.6) | | | | | | (10.0–10.0) | | | | | |
| Mean (SD) | | 26.8 | | | | 144.7 | | | | 43.4 | | | | | | 68.8 | | | | | | 51.3 | | | | | | 10 | | | | | |
|  |  | (3.9) | | | | (38.6) | | | | (16.0) | | | | | | (12.0) | | | | | | (19.0) | | | | | | (0.4) | | | | | |
| **Patients among dose-intervals, %** | | 7d | | 7.7% | | 3d | | 58.2% | | 7d | | | 100.0% | | | 8d | | | 18.3% | | | 7d | | | 68.8% | | | 7d | | | 100.0% | | |
|  |  | 10d | | 10.4% | | 3.5d | | 16.7% | |  | | |  | | | 9d | | | 16.2% | | | 8d | | | 5.8% | | |  | | |  | | |
|  |  | 14d | | 81.9% | | 4d | | 25.1% | |  | | |  | | | 10d | | | 18.0% | | | 9d | | | 5.2% | | |  | | |  | | |
|  |  |  | |  | |  | |  | |  | | |  | | | 11d | | | 16.4% | | | 10d | | | 5.6% | | |  | | |  | | |
|  |  |  | |  | |  | |  | |  | | |  | | | 12d | | | 11.6% | | | 11d | | | 4.8% | | |  | | |  | | |
|  |  |  | |  | |  | |  | |  | | |  | | | 13d | | | 15.6% | | | 12d | | | 3.5% | | |  | | |  | | |
|  |  |  | |  | |  | |  | |  | | |  | | | 14d | | | 3.9% | | | 13d | | | 5.1% | | |  | | |  | | |
|  |  |  | |  | |  | |  | |  | | |  | | | 15d | | | 0.0% | | | 14d | | | 1.2% | | |  | | |  | | |
|  |  |  | |  | |  | |  | |  | | |  | | | 16d | | | 0.0% | | | 15d | | | 0.0% | | |  | | |  | | |
|  |  |  | |  | |  | |  | |  | | |  | | | 17d | | | 0.0% | | | 16d | | | 0.0% | | |  | | |  | | |
|  |  |  | |  | |  | |  | |  | | |  | | | 18d | | | 0.0% | | | 17d | | | 0.0% | | |  | | |  | | |
|  |  |  | |  | |  | |  | |  | | |  | | | 19d | | | 0.0% | | | 18d | | | 0.0% | | |  | | |  | | |
|  |  |  | |  | |  | |  | |  | | |  | | | 20d | | | 0.0% | | | 19d | | | 0.0% | | |  | | |  | | |
|  |  |  | |  | |  | |  | |  | | |  | | | 21d | | | 0.0% | | | 20d | | | 0.0% | | |  | | |  | | |
|  |  |  | |  | |  | |  | |  | | |  | | |  | | |  | | | 21d | | | 0.0% | | |  | | |  | | |
| **Ratio between weekly dose (IU/kg) FIX product and rIX-FP** Median (IQR) | |  | | | | 5.3 | | | | 1.5 | | | | | | 2.5 | | | | | | 1.8 | | | | | | 0.4 | | | | | |
|  |  |  | | | | (4.5–7.3) | | | | (1.2–2.0) | | | | | | (2.2–3.1) | | | | | | (1.3–2.5) | | | | | | (0.4–0.4) | | | | | |
| Mean (SD) | |  | | | | 5.5 | | | | 1.6 | | | | | | 2.6 | | | | | | 1.9 | | | | | | 0.4 | | | | | |
|  |  |  | | | | (1.6) | | | | (0.6) | | | | | | (0.5) | | | | | | (0.8) | | | | | | (0.0) | | | | | |
| ^Ⱡ^Patients were randomly assigned to individualized interval prophylaxis from a Bernoulli distribution with a probability 0.315, based on data from clinical trial 998HB102 (NCT01027364). In the simulations underlying the results of this table, 68.8% of the simulated patients were assigned to receive weekly prophylaxis and 31.3% to individualized interval prophylaxis. | | | | | | | | | | | | | | | | | | | | | | | | | | | | | | | | | |
| **Patients 12 to 17 \| Target 2%; Control 7.5%** | | |  | |  | |  | |  | | |  | | |  | | |  | | |  | | |  | | |  | | |  | | |  |
|  | **rIX-FP** | | | | **rFIX** | | | | **rFIXFc** weekly | | | | | | **rFIXFc** ind. Interval | | | | | | **rFIXFc^Ⱡ^** weekly and ind.interval | | | | | | **N9-GP** | | | | | |  |
| **Steady-state FIX trough levels** Median (IQR) | 6.0 | | | | 2.0 | | | | 2.0 | | | | | | 2.2 | | | | | | 2.0 | | | | | | 4.1 | | | | | |  |
|  | (4.7–7.0) | | | | (1.5–2.0) | | | | (2.0–2.0) | | | | | | (2.1–2.4) | | | | | | (2.0–2.1) | | | | | | (3.3–5.1) | | | | | |  |
| Mean (SD) | 6.1 | | | | 1.7 | | | | 2.0 | | | | | | 2.3 | | | | | | 2.1 | | | | | | 4.3 | | | | | |  |
|  | (2.1) | | | | (0.5) | | | | (0.1) | | | | | | (0.4) | | | | | | (0.3) | | | | | | (1.3) | | | | | |  |
| **Patients below target, %** | 0.0% | | | | 37.8% | | | | 2.7% | | | | | | 6.3% | | | | | | 3.8% | | | | | | 0.0% | | | | | |  |
| **Dose (IU/kg per week)** Median (IQR) | 25.0 | | | | 149.0 | | | | 40.0 | | | | | | 70.0 | | | | | | 50.0 | | | | | | 10.0 | | | | | |  |
|  | (25.0–35.0) | | | | (120.8–182.0) | | | | (32.0–52.0) | | | | | | (58.3–77.8) | | | | | | (35.5–63.6) | | | | | | (10.0–10.0) | | | | | |  |
| Mean (SD) | 29.2 | | | | 144.7 | | | | 43.4 | | | | | | 68.9 | | | | | | 51.3 | | | | | | 10 | | | | | |  |
|  | (5.0) | | | | (38.6) | | | | (16.0) | | | | | | (11.8) | | | | | | (19.0) | | | | | | (0.4) | | | | | |  |
| **Patients among dose-intervals, %** | 7d | | 25.0% | | 3d | | 58.2% | | 7d | | | 100.0% | | | 8d | | | 18.3% | | | 7d | | | 68.8% | | | 7d | | | 100.0% | | |  |
|  | 10d | | 17.3% | | 3.5d | | 16.7% | |  | | |  | | | 9d | | | 16.2% | | | 8d | | | 5.8% | | |  | | |  | | |  |
|  | 14d | | 57.7% | | 4d | | 25.1% | |  | | |  | | | 10d | | | 18.0% | | | 9d | | | 5.2% | | |  | | |  | | |  |
|  |  | |  | |  | |  | |  | | |  | | | 11d | | | 16.4% | | | 10d | | | 5.6% | | |  | | |  | | |  |
|  |  | |  | |  | |  | |  | | |  | | | 12d | | | 11.6% | | | 11d | | | 4.8% | | |  | | |  | | |  |
|  |  | |  | |  | |  | |  | | |  | | | 13d | | | 19.2% | | | 12d | | | 3.5% | | |  | | |  | | |  |
|  |  | |  | |  | |  | |  | | |  | | | 14d | | | 0.2% | | | 13d | | | 6.2% | | |  | | |  | | |  |
|  |  | |  | |  | |  | |  | | |  | | | 15d | | | 0.0% | | | 14d | | | 0.1% | | |  | | |  | | |  |
|  |  | |  | |  | |  | |  | | |  | | | 16d | | | 0.0% | | | 15d | | | 0.0% | | |  | | |  | | |  |
|  |  | |  | |  | |  | |  | | |  | | | 17d | | | 0.0% | | | 16d | | | 0.0% | | |  | | |  | | |  |
|  |  | |  | |  | |  | |  | | |  | | | 18d | | | 0.0% | | | 17d | | | 0.0% | | |  | | |  | | |  |
|  |  | |  | |  | |  | |  | | |  | | | 19d | | | 0.0% | | | 18d | | | 0.0% | | |  | | |  | | |  |
|  |  | |  | |  | |  | |  | | |  | | | 20d | | | 0.0% | | | 19d | | | 0.0% | | |  | | |  | | |  |
|  |  | |  | |  | |  | |  | | |  | | | 21d | | | 0.0% | | | 20d | | | 0.0% | | |  | | |  | | |  |
|  |  | |  | |  | |  | |  | | |  | | |  | | |  | | | 21d | | | 0.0% | | |  | | |  | | |  |
| **Ratio between weekly dose (IU/kg) FIX product and rIX-FP** Median (IQR) |  | | | | 5.2 | | | | 1.4 | | | | | | 2.3 | | | | | | 1.7 | | | | | | 0.4 | | | | | |  |
|  |  | | | | (3.9–6.3) | | | | (1.1–1.8) | | | | | | (2.0–2.8) | | | | | | (1.2–2.3) | | | | | | (0.3–0.4) | | | | | |  |
| Mean (SD) |  | | | | 5.1 | | | | 1.5 | | | | | | 2.4 | | | | | | 1.8 | | | | | | 0.4 | | | | | |  |
|  |  | | | | (1.6) | | | | (0.6) | | | | | | (0.6) | | | | | | (0.7) | | | | | | (0.1) | | | | | |  |
| ^Ⱡ^Patients were randomly assigned to individualized interval prophylaxis from a Bernoulli distribution with a probability 0.315, based on data from clinical trial 998HB102 (NCT01027364). In the simulations underlying the results of this table, 68.8% of the simulated patients were assigned to receive weekly prophylaxis and 31.3% to individualized interval prophylaxis. | | | | | | | | | | | | | | | | | | | | | | | | | | | | | | | | |  |
| **Patients 12 to 17 \| Target 2%; Control 10%** | | |  | |  | |  | |  | | |  | | |  | | |  | | |  | | |  | | |  | | |  | | |  |
|  | **rIX-FP** | | | | **rFIX** | | | | **rFIXFc** weekly | | | | | | **rFIXFc** ind. Interval | | | | | | **rFIXFc^Ⱡ^** weekly and ind.interval | | | | | | **N9-GP** | | | | | |  |
| **Steady-state FIX trough levels** Median (IQR) | 7.7 | | | | 2.0 | | | | 2.0 | | | | | | 2.2 | | | | | | 2.0 | | | | | | 4.1 | | | | | |  |
|  | (6.0–9.0) | | | | (1.5–2.0) | | | | (2.0–2.0) | | | | | | (2.1–2.4) | | | | | | (2.0–2.1) | | | | | | (3.3–5.1) | | | | | |  |
| Mean (SD) | 7.5 | | | | 1.7 | | | | 2.0 | | | | | | 2.3 | | | | | | 2.1 | | | | | | 4.3 | | | | | |  |
|  | (2.1) | | | | (0.5) | | | | (0.1) | | | | | | (0.4) | | | | | | (0.3) | | | | | | (1.3) | | | | | |  |
| **Patients below target, %** | 0.0% | | | | 37.8% | | | | 2.7% | | | | | | 6.3% | | | | | | 3.8% | | | | | | 0.0% | | | | | |  |
| **Dose (IU/kg per week)** Median (IQR) | 35.0 | | | | 149.0 | | | | 40.0 | | | | | | 70.0 | | | | | | 50.0 | | | | | | 10.0 | | | | | |  |
|  | (25.0–35.0) | | | | (120.8–182.0) | | | | (32.0–52.0) | | | | | | (58.3–77.8) | | | | | | (35.5–63.6) | | | | | | (10.0–10.0) | | | | | |  |
| Mean (SD) | 31.4 | | | | 144.7 | | | | 43.4 | | | | | | 68.9 | | | | | | 51.3 | | | | | | 10 | | | | | |  |
|  | (4.8) | | | | (38.6) | | | | (16.0) | | | | | | (11.7) | | | | | | (19.0) | | | | | | (0.4) | | | | | |  |
| **Patients among dose-intervals, %** | 7d | | 47.4% | | 3d | | 58.2% | | 7d | | | 100.0% | | | 8d | | | 18.3% | | | 7d | | | 68.8% | | | 7d | | | 100.0% | | |  |
|  | 10d | | 16.7% | | 3.5d | | 16.7% | |  | | |  | | | 9d | | | 16.2% | | | 8d | | | 5.8% | | |  | | |  | | |  |
|  | 14d | | 35.9% | | 4d | | 25.1% | |  | | |  | | | 10d | | | 18.0% | | | 9d | | | 5.2% | | |  | | |  | | |  |
|  |  | |  | |  | |  | |  | | |  | | | 11d | | | 16.4% | | | 10d | | | 5.6% | | |  | | |  | | |  |
|  |  | |  | |  | |  | |  | | |  | | | 12d | | | 11.6% | | | 11d | | | 4.8% | | |  | | |  | | |  |
|  |  | |  | |  | |  | |  | | |  | | | 13d | | | 19.5% | | | 12d | | | 3.5% | | |  | | |  | | |  |
|  |  | |  | |  | |  | |  | | |  | | | 14d | | | 0.0% | | | 13d | | | 6.3% | | |  | | |  | | |  |
|  |  | |  | |  | |  | |  | | |  | | | 15d | | | 0.0% | | | 14d | | | 0.0% | | |  | | |  | | |  |
|  |  | |  | |  | |  | |  | | |  | | | 16d | | | 0.0% | | | 15d | | | 0.0% | | |  | | |  | | |  |
|  |  | |  | |  | |  | |  | | |  | | | 17d | | | 0.0% | | | 16d | | | 0.0% | | |  | | |  | | |  |
|  |  | |  | |  | |  | |  | | |  | | | 18d | | | 0.0% | | | 17d | | | 0.0% | | |  | | |  | | |  |
|  |  | |  | |  | |  | |  | | |  | | | 19d | | | 0.0% | | | 18d | | | 0.0% | | |  | | |  | | |  |
|  |  | |  | |  | |  | |  | | |  | | | 20d | | | 0.0% | | | 19d | | | 0.0% | | |  | | |  | | |  |
|  |  | |  | |  | |  | |  | | |  | | | 21d | | | 0.0% | | | 20d | | | 0.0% | | |  | | |  | | |  |
|  |  | |  | |  | |  | |  | | |  | | |  | | |  | | | 21d | | | 0.0% | | |  | | |  | | |  |
| **Ratio between weekly dose (IU/kg) FIX product and rIX-FP** Median (IQR) |  | | | | 5.1 | | | | 1.3 | | | | | | 2.2 | | | | | | 1.5 | | | | | | 0.3 | | | | | |  |
|  |  | | | | (3.7–5.2) | | | | (1.0–1.7) | | | | | | (1.8–2.5) | | | | | | (1.1–2.2) | | | | | | (0.3–0.4) | | | | | |  |
| Mean (SD) |  | | | | 4.7 | | | | 1.4 | | | | | | 2.2 | | | | | | 1.7 | | | | | | 0.3 | | | | | |  |
|  |  | | | | (1.5) | | | | (0.6) | | | | | | (0.5) | | | | | | (0.7) | | | | | | (0.1) | | | | | |  |
| ^Ⱡ^Patients were randomly assigned to individualized interval prophylaxis from a Bernoulli distribution with a probability 0.315, based on data from clinical trial 998HB102 (NCT01027364). In the simulations underlying the results of this table, 68.8% of the simulated patients were assigned to receive weekly prophylaxis and 31.3% to individualized interval prophylaxis. | | | | | | | | | | | | | | | | | | | | | | | | | | | | | | | | |  |
| **Patients 12 to 17 \| Target 2%; Control 15%** | | |  | |  | |  | |  | | |  | | |  | | |  | | |  | | |  | | |  | | |  | | |  |
|  | **rIX-FP** | | | | **rFIX** | | | | **rFIXFc** weekly | | | | | | **rFIXFc** ind. Interval | | | | | | **rFIXFc^Ⱡ^** weekly and ind.interval | | | | | | **N9-GP** | | | | | |  |
| **Steady-state FIX trough levels** Median (IQR) | 9.7 | | | | 2.0 | | | | 2.0 | | | | | | 2.2 | | | | | | 2.0 | | | | | | 4.1 | | | | | |  |
|  | (7.5–12.4) | | | | (1.5–2.0) | | | | (2.0–2.0) | | | | | | (2.1–2.4) | | | | | | (2.0–2.1) | | | | | | (3.3–5.1) | | | | | |  |
| Mean (SD) | 9.8 | | | | 1.7 | | | | 2.0 | | | | | | 2.3 | | | | | | 2.1 | | | | | | 4.3 | | | | | |  |
|  | (3.1) | | | | (0.5) | | | | (0.1) | | | | | | (0.4) | | | | | | (0.3) | | | | | | (1.3) | | | | | |  |
| **Patients below target, %** | 0.0% | | | | 37.8% | | | | 2.7% | | | | | | 6.3% | | | | | | 3.8% | | | | | | 0.0% | | | | | |  |
| **Dose (IU/kg per week)** Median (IQR) | 35.0 | | | | 149.0 | | | | 40.0 | | | | | | 70.0 | | | | | | 50.0 | | | | | | 10.0 | | | | | |  |
|  | (35.0–35.0) | | | | (120.8–182.0) | | | | (32.0–52.0) | | | | | | (58.3–77.8) | | | | | | (35.5–63.6) | | | | | | (10.0–10.0) | | | | | |  |
| Mean (SD) | 34 | | | | 144.7 | | | | 43.4 | | | | | | 68.9 | | | | | | 51.3 | | | | | | 10 | | | | | |  |
|  | (3.1) | | | | (38.6) | | | | (16.0) | | | | | | (11.7) | | | | | | (19.0) | | | | | | (0.4) | | | | | |  |
| **Patients among dose-intervals, %** | 7d | | 81.0% | | 3d | | 58.2% | | 7d | | | 100.0% | | | 8d | | | 18.3% | | | 7d | | | 68.8% | | | 7d | | | 100.0% | | |  |
|  | 10d | | 8.6% | | 3.5d | | 16.7% | |  | | |  | | | 9d | | | 16.2% | | | 8d | | | 5.8% | | |  | | |  | | |  |
|  | 14d | | 10.4% | | 4d | | 25.1% | |  | | |  | | | 10d | | | 18.0% | | | 9d | | | 5.2% | | |  | | |  | | |  |
|  |  | |  | |  | |  | |  | | |  | | | 11d | | | 16.4% | | | 10d | | | 5.6% | | |  | | |  | | |  |
|  |  | |  | |  | |  | |  | | |  | | | 12d | | | 11.6% | | | 11d | | | 4.8% | | |  | | |  | | |  |
|  |  | |  | |  | |  | |  | | |  | | | 13d | | | 19.5% | | | 12d | | | 3.5% | | |  | | |  | | |  |
|  |  | |  | |  | |  | |  | | |  | | | 14d | | | 0.0% | | | 13d | | | 6.3% | | |  | | |  | | |  |
|  |  | |  | |  | |  | |  | | |  | | | 15d | | | 0.0% | | | 14d | | | 0.0% | | |  | | |  | | |  |
|  |  | |  | |  | |  | |  | | |  | | | 16d | | | 0.0% | | | 15d | | | 0.0% | | |  | | |  | | |  |
|  |  | |  | |  | |  | |  | | |  | | | 17d | | | 0.0% | | | 16d | | | 0.0% | | |  | | |  | | |  |
|  |  | |  | |  | |  | |  | | |  | | | 18d | | | 0.0% | | | 17d | | | 0.0% | | |  | | |  | | |  |
|  |  | |  | |  | |  | |  | | |  | | | 19d | | | 0.0% | | | 18d | | | 0.0% | | |  | | |  | | |  |
|  |  | |  | |  | |  | |  | | |  | | | 20d | | | 0.0% | | | 19d | | | 0.0% | | |  | | |  | | |  |
|  |  | |  | |  | |  | |  | | |  | | | 21d | | | 0.0% | | | 20d | | | 0.0% | | |  | | |  | | |  |
|  |  | |  | |  | |  | |  | | |  | | |  | | |  | | | 21d | | | 0.0% | | |  | | |  | | |  |
| **Ratio between weekly dose (IU/kg) FIX product and rIX-FP** Median (IQR) |  | | | | 4.4 | | | | 1.2 | | | | | | 2.0 | | | | | | 1.5 | | | | | | 0.3 | | | | | |  |
|  |  | | | | (3.5–5.2) | | | | (0.9–1.5) | | | | | | (1.7–2.3) | | | | | | (1.0–2.0) | | | | | | (0.3–0.3) | | | | | |  |
| Mean (SD) |  | | | | 4.3 | | | | 1.3 | | | | | | 2.0 | | | | | | 1.5 | | | | | | 0.3 | | | | | |  |
|  |  | | | | (1.2) | | | | (0.5) | | | | | | (0.4) | | | | | | (0.6) | | | | | | (0.0) | | | | | |  |
| ^Ⱡ^Patients were randomly assigned to individualized interval prophylaxis from a Bernoulli distribution with a probability 0.315, based on data from clinical trial 998HB102 (NCT01027364). In the simulations underlying the results of this table, 68.8% of the simulated patients were assigned to receive weekly prophylaxis and 31.3% to individualized interval prophylaxis. | | | | | | | | | | | | | | | | | | | | | | | | | | | | | | | | |  |
| **Patients 12 to 17 \| Target 2%; Control 20%** | | |  | |  | |  | |  | |  | | |  | | |  | | |  | | |  | | |  | | |  | | |  |  |
|  | **rIX-FP** | | | | **rFIX** | | | | **rFIXFc** weekly | | | | | **rFIXFc** ind. Interval | | | | | | **rFIXFc^Ⱡ^** weekly and ind.interval | | | | | | **N9-GP** | | | | | |  |  |
| **Steady-state FIX trough levels** Median (IQR) | 10.3 | | | | 2.0 | | | | 2.0 | | | | | 2.2 | | | | | | 2.0 | | | | | | 4.1 | | | | | |  |  |
|  | (7.5–13.6) | | | | (1.5–2.0) | | | | (2.0–2.0) | | | | | (2.1–2.4) | | | | | | (2.0–2.1) | | | | | | (3.3–5.1) | | | | | |  |  |
| Mean (SD) | 10.7 | | | | 1.7 | | | | 2.0 | | | | | 2.3 | | | | | | 2.1 | | | | | | 4.3 | | | | | |  |  |
|  | (4.1) | | | | (0.5) | | | | (0.1) | | | | | (0.4) | | | | | | (0.3) | | | | | | (1.3) | | | | | |  |  |
| **Patients below target, %** | 0.0% | | | | 37.8% | | | | 2.7% | | | | | 6.3% | | | | | | 3.8% | | | | | | 0.0% | | | | | |  |  |
| **Dose (IU/kg per week)** Median (IQR) | 35.0 | | | | 149.0 | | | | 40.0 | | | | | 70.0 | | | | | | 50.0 | | | | | | 10.0 | | | | | |  |  |
|  | (35.0–35.0) | | | | (120.8–182.0) | | | | (32.0–52.0) | | | | | (58.3–77.8) | | | | | | (35.5–63.6) | | | | | | (10.0–10.0) | | | | | |  |  |
| Mean (SD) | 34.7 | | | | 144.7 | | | | 43.4 | | | | | 68.9 | | | | | | 51.3 | | | | | | 10 | | | | | |  |  |
|  | (1.7) | | | | (38.6) | | | | (16.0) | | | | | (11.7) | | | | | | (19.0) | | | | | | (0.4) | | | | | |  |  |
| **Patients among dose-intervals, %** | 7d | | 94.7% | | 3d | | 58.2% | | 7d | | 100.0% | | | 8d | | | 18.3% | | | 7d | | | 68.8% | | | 7d | | | 100.0% | | |  |  |
|  | 10d | | 2.6% | | 3.5d | | 16.7% | |  | |  | | | 9d | | | 16.2% | | | 8d | | | 5.8% | | |  | | |  | | |  |  |
|  | 14d | | 2.7% | | 4d | | 25.1% | |  | |  | | | 10d | | | 18.0% | | | 9d | | | 5.2% | | |  | | |  | | |  |  |
|  |  | |  | |  | |  | |  | |  | | | 11d | | | 16.4% | | | 10d | | | 5.6% | | |  | | |  | | |  |  |
|  |  | |  | |  | |  | |  | |  | | | 12d | | | 11.6% | | | 11d | | | 4.8% | | |  | | |  | | |  |  |
|  |  | |  | |  | |  | |  | |  | | | 13d | | | 19.5% | | | 12d | | | 3.5% | | |  | | |  | | |  |  |
|  |  | |  | |  | |  | |  | |  | | | 14d | | | 0.0% | | | 13d | | | 6.3% | | |  | | |  | | |  |  |
|  |  | |  | |  | |  | |  | |  | | | 15d | | | 0.0% | | | 14d | | | 0.0% | | |  | | |  | | |  |  |
|  |  | |  | |  | |  | |  | |  | | | 16d | | | 0.0% | | | 15d | | | 0.0% | | |  | | |  | | |  |  |
|  |  | |  | |  | |  | |  | |  | | | 17d | | | 0.0% | | | 16d | | | 0.0% | | |  | | |  | | |  |  |
|  |  | |  | |  | |  | |  | |  | | | 18d | | | 0.0% | | | 17d | | | 0.0% | | |  | | |  | | |  |  |
|  |  | |  | |  | |  | |  | |  | | | 19d | | | 0.0% | | | 18d | | | 0.0% | | |  | | |  | | |  |  |
|  |  | |  | |  | |  | |  | |  | | | 20d | | | 0.0% | | | 19d | | | 0.0% | | |  | | |  | | |  |  |
|  |  | |  | |  | |  | |  | |  | | | 21d | | | 0.0% | | | 20d | | | 0.0% | | |  | | |  | | |  |  |
|  |  | |  | |  | |  | |  | |  | | |  | | |  | | | 21d | | | 0.0% | | |  | | |  | | |  |  |
| **Ratio between weekly dose (IU/kg) FIX product and rIX-FP** Median (IQR) |  | | | | 4.3 | | | | 1.2 | | | | | 2.0 | | | | | | 1.4 | | | | | | 0.3 | | | | | |  |  |
|  |  | | | | (3.5–5.2) | | | | (0.9–1.5) | | | | | (1.7–2.2) | | | | | | (1.0–1.8) | | | | | | (0.3–0.3) | | | | | |  |  |
| Mean (SD) |  | | | | 4.2 | | | | 1.3 | | | | | 2.0 | | | | | | 1.5 | | | | | | 0.3 | | | | | |  |  |
|  |  | | | | (1.1) | | | | (0.5) | | | | | (0.4) | | | | | | (0.6) | | | | | | (0.0) | | | | | |  |  |
| ^Ⱡ^Patients were randomly assigned to individualized interval prophylaxis from a Bernoulli distribution with a probability 0.315, based on data from clinical trial 998HB102 (NCT01027364). In the simulations underlying the results of this table, 68.8% of the simulated patients were assigned to receive weekly prophylaxis and 31.3% to individualized interval prophylaxis. | | | | | | | | | | | | | | | | | | | | | | | | | | | | | | | |  |  |
| **Patients 12 to 17 \| Target 3%; Control 5%** | |  | |  | |  | |  | |  | | |  | | |  | | |  | | |  | | |  | | |  | | |  | | |
|  | | **rIX-FP** | | | | **rFIX** | | | | **rFIXFc** weekly | | | | | | **rFIXFc** ind. Interval | | | | | | **rFIXFc^Ⱡ^** weekly and ind.interval | | | | | | **N9-GP** | | | | | |
| **Steady-state FIX trough levels** Median (IQR) | | 4.4 | | | | 2.6 | | | | 3.0 | | | | | | 3.2 | | | | | | 3.0 | | | | | | 4.1 | | | | | |
|  |  | (3.4–5.9) | | | | (1.5–3.0) | | | | (3.0–3.0) | | | | | | (2.9–3.4) | | | | | | (3.0–3.0) | | | | | | (3.3–5.1) | | | | | |
| Mean (SD) | | 5.1 | | | | 2.2 | | | | 2.9 | | | | | | 3.1 | | | | | | 3.0 | | | | | | 4.4 | | | | | |
|  |  | (2.3) | | | | (0.9) | | | | (0.3) | | | | | | (0.6) | | | | | | (0.4) | | | | | | (1.2) | | | | | |
| **Patients below target, %** | | 0.1% | | | | 57.7% | | | | 15.6% | | | | | | 26.5% | | | | | | 19.2% | | | | | | 0.0% | | | | | |
| **Dose (IU/kg per week)** Median (IQR) | | 25.0 | | | | 182.0 | | | | 60.0 | | | | | | 77.8 | | | | | | 69.5 | | | | | | 10.0 | | | | | |
|  |  | (25.0–28.7) | | | | (142.0–182.0) | | | | (47.5–77.5) | | | | | | (70.0–87.5) | | | | | | (52.5–87.5) | | | | | | (10.0–10.0) | | | | | |
| Mean (SD) | | 27.4 | | | | 161.9 | | | | 61.7 | | | | | | 78.6 | | | | | | 67 | | | | | | 10.4 | | | | | |
|  |  | (4.1) | | | | (29.4) | | | | (17.8) | | | | | | (10.2) | | | | | | (17.7) | | | | | | (1.4) | | | | | |
| **Patients among dose-intervals, %** | | 7d | | 7.7% | | 3d | | 77.0% | | 7d | | | 100.0% | | | 8d | | | 48.7% | | | 7d | | | 68.8% | | | 7d | | | 100.0% | | |
|  |  | 10d | | 10.4% | | 3.5d | | 11.4% | |  | | |  | | | 9d | | | 20.5% | | | 8d | | | 15.4% | | |  | | |  | | |
|  |  | 14d | | 81.9% | | 4d | | 11.6% | |  | | |  | | | 10d | | | 16.0% | | | 9d | | | 6.2% | | |  | | |  | | |
|  |  |  | |  | |  | |  | |  | | |  | | | 11d | | | 8.5% | | | 10d | | | 5.0% | | |  | | |  | | |
|  |  |  | |  | |  | |  | |  | | |  | | | 12d | | | 4.0% | | | 11d | | | 2.8% | | |  | | |  | | |
|  |  |  | |  | |  | |  | |  | | |  | | | 13d | | | 0.5% | | | 12d | | | 1.2% | | |  | | |  | | |
|  |  |  | |  | |  | |  | |  | | |  | | | 14d | | | 1.8% | | | 13d | | | 0.1% | | |  | | |  | | |
|  |  |  | |  | |  | |  | |  | | |  | | | 15d | | | 0.0% | | | 14d | | | 0.5% | | |  | | |  | | |
|  |  |  | |  | |  | |  | |  | | |  | | | 16d | | | 0.0% | | | 15d | | | 0.0% | | |  | | |  | | |
|  |  |  | |  | |  | |  | |  | | |  | | | 17d | | | 0.0% | | | 16d | | | 0.0% | | |  | | |  | | |
|  |  |  | |  | |  | |  | |  | | |  | | | 18d | | | 0.0% | | | 17d | | | 0.0% | | |  | | |  | | |
|  |  |  | |  | |  | |  | |  | | |  | | | 19d | | | 0.0% | | | 18d | | | 0.0% | | |  | | |  | | |
|  |  |  | |  | |  | |  | |  | | |  | | | 20d | | | 0.0% | | | 19d | | | 0.0% | | |  | | |  | | |
|  |  |  | |  | |  | |  | |  | | |  | | | 21d | | | 0.0% | | | 20d | | | 0.0% | | |  | | |  | | |
|  |  |  | |  | |  | |  | |  | | |  | | |  | | |  | | | 21d | | | 0.0% | | |  | | |  | | |
| **Ratio between weekly dose (IU/kg) FIX product and rIX-FP** Median (IQR) | |  | | | | 6.2 | | | | 2.2 | | | | | | 3.0 | | | | | | 2.5 | | | | | | 0.4 | | | | | |
|  |  |  | | | | (5.2–7.3) | | | | (1.7–2.8) | | | | | | (2.5–3.5) | | | | | | (1.9–3.1) | | | | | | (0.4–0.4) | | | | | |
| Mean (SD) | |  | | | | 6.0 | | | | 2.3 | | | | | | 2.9 | | | | | | 2.5 | | | | | | 0.4 | | | | | |
|  |  |  | | | | (1.3) | | | | (0.7) | | | | | | (0.5) | | | | | | (0.7) | | | | | | (0.1) | | | | | |
| ^Ⱡ^Patients were randomly assigned to individualized interval prophylaxis from a Bernoulli distribution with a probability 0.315, based on data from clinical trial 998HB102 (NCT01027364). In the simulations underlying the results of this table, 68.8% of the simulated patients were assigned to receive weekly prophylaxis and 31.3% to individualized interval prophylaxis. | | | | | | | | | | | | | | | | | | | | | | | | | | | | | | | | | |
| **Patients 12 to 17 \| Target 3%; Control 7.5%** | | |  | |  | |  | |  | | |  | | |  | | |  | | |  | | |  | | |  | | |  | | |  |
|  | **rIX-FP** | | | | **rFIX** | | | | **rFIXFc** weekly | | | | | | **rFIXFc** ind. Interval | | | | | | **rFIXFc^Ⱡ^** weekly and ind.interval | | | | | | **N9-GP** | | | | | |  |
| **Steady-state FIX trough levels** Median (IQR) | 6.0 | | | | 2.6 | | | | 3.0 | | | | | | 3.2 | | | | | | 3.0 | | | | | | 4.1 | | | | | |  |
|  | (4.7–7.0) | | | | (1.5–3.0) | | | | (3.0–3.0) | | | | | | (2.9–3.4) | | | | | | (3.0–3.0) | | | | | | (3.3–5.1) | | | | | |  |
| Mean (SD) | 6.1 | | | | 2.2 | | | | 2.9 | | | | | | 3.1 | | | | | | 3.0 | | | | | | 4.4 | | | | | |  |
|  | (2.0) | | | | (0.9) | | | | (0.3) | | | | | | (0.6) | | | | | | (0.4) | | | | | | (1.2) | | | | | |  |
| **Patients below target, %** | 0.1% | | | | 57.7% | | | | 15.6% | | | | | | 26.5% | | | | | | 19.2% | | | | | | 0.0% | | | | | |  |
| **Dose (IU/kg per week)** Median (IQR) | 25.0 | | | | 182.0 | | | | 60.0 | | | | | | 77.8 | | | | | | 69.0 | | | | | | 10.0 | | | | | |  |
|  | (25.0–35.0) | | | | (142.0–182.0) | | | | (47.5–77.5) | | | | | | (70.0–87.5) | | | | | | (53.0–87.5) | | | | | | (10.0–10.0) | | | | | |  |
| Mean (SD) | 29.3 | | | | 161.9 | | | | 61.7 | | | | | | 78.4 | | | | | | 66.9 | | | | | | 10.4 | | | | | |  |
|  | (5.1) | | | | (29.4) | | | | (17.8) | | | | | | (10.6) | | | | | | (17.7) | | | | | | (1.4) | | | | | |  |
| **Patients among dose-intervals, %** | 7d | | 25.0% | | 3d | | 77.0% | | 7d | | | 100.0% | | | 8d | | | 48.7% | | | 7d | | | 68.8% | | | 7d | | | 100.0% | | |  |
|  | 10d | | 17.3% | | 3.5d | | 11.4% | |  | | |  | | | 9d | | | 20.5% | | | 8d | | | 15.4% | | |  | | |  | | |  |
|  | 14d | | 57.7% | | 4d | | 11.6% | |  | | |  | | | 10d | | | 13.9% | | | 9d | | | 6.2% | | |  | | |  | | |  |
|  |  | |  | |  | |  | |  | | |  | | | 11d | | | 8.5% | | | 10d | | | 4.3% | | |  | | |  | | |  |
|  |  | |  | |  | |  | |  | | |  | | | 12d | | | 4.4% | | | 11d | | | 2.8% | | |  | | |  | | |  |
|  |  | |  | |  | |  | |  | | |  | | | 13d | | | 3.8% | | | 12d | | | 1.3% | | |  | | |  | | |  |
|  |  | |  | |  | |  | |  | | |  | | | 14d | | | 0.2% | | | 13d | | | 1.2% | | |  | | |  | | |  |
|  |  | |  | |  | |  | |  | | |  | | | 15d | | | 0.0% | | | 14d | | | 0.1% | | |  | | |  | | |  |
|  |  | |  | |  | |  | |  | | |  | | | 16d | | | 0.0% | | | 15d | | | 0.0% | | |  | | |  | | |  |
|  |  | |  | |  | |  | |  | | |  | | | 17d | | | 0.0% | | | 16d | | | 0.0% | | |  | | |  | | |  |
|  |  | |  | |  | |  | |  | | |  | | | 18d | | | 0.0% | | | 17d | | | 0.0% | | |  | | |  | | |  |
|  |  | |  | |  | |  | |  | | |  | | | 19d | | | 0.0% | | | 18d | | | 0.0% | | |  | | |  | | |  |
|  |  | |  | |  | |  | |  | | |  | | | 20d | | | 0.0% | | | 19d | | | 0.0% | | |  | | |  | | |  |
|  |  | |  | |  | |  | |  | | |  | | | 21d | | | 0.0% | | | 20d | | | 0.0% | | |  | | |  | | |  |
|  |  | |  | |  | |  | |  | | |  | | |  | | |  | | | 21d | | | 0.0% | | |  | | |  | | |  |
| **Ratio between weekly dose (IU/kg) FIX product and rIX-FP** Median (IQR) |  | | | | 5.2 | | | | 2.1 | | | | | | 2.5 | | | | | | 2.3 | | | | | | 0.4 | | | | | |  |
|  |  | | | | (5.0–7.3) | | | | (1.6–2.5) | | | | | | (2.3–3.5) | | | | | | (1.8–2.8) | | | | | | (0.3–0.4) | | | | | |  |
| Mean (SD) |  | | | | 5.7 | | | | 2.2 | | | | | | 2.8 | | | | | | 2.3 | | | | | | 0.4 | | | | | |  |
|  |  | | | | (1.4) | | | | (0.7) | | | | | | (0.6) | | | | | | (0.7) | | | | | | (0.1) | | | | | |  |
| ^Ⱡ^Patients were randomly assigned to individualized interval prophylaxis from a Bernoulli distribution with a probability 0.315, based on data from clinical trial 998HB102 (NCT01027364). In the simulations underlying the results of this table, 68.8% of the simulated patients were assigned to receive weekly prophylaxis and 31.3% to individualized interval prophylaxis. | | | | | | | | | | | | | | | | | | | | | | | | | | | | | | | | |  |
| **Patients 12 to 17 \| Target 3%; Control 10%** |  | |  | |  | |  | |  | | |  | | |  | | |  | | |  | | |  | | |  | | |  | | |  |
|  | **rIX-FP** | | | | **rFIX** | | | | **rFIXFc** weekly | | | | | | **rFIXFc** ind. Interval | | | | | | **rFIXFc^Ⱡ^** weekly and ind.interval | | | | | | **N9-GP** | | | | | |  |
| **Steady-state FIX trough levels** Median (IQR) | 7.7 | | | | 2.6 | | | | 3.0 | | | | | | 3.2 | | | | | | 3.0 | | | | | | 4.1 | | | | | |  |
|  | (6.0–9.0) | | | | (1.5–3.0) | | | | (3.0–3.0) | | | | | | (2.9–3.4) | | | | | | (3.0–3.0) | | | | | | (3.3–5.1) | | | | | |  |
| Mean (SD) | 7.5 | | | | 2.2 | | | | 2.9 | | | | | | 3.1 | | | | | | 3.0 | | | | | | 4.4 | | | | | |  |
|  | (2.1) | | | | (0.9) | | | | (0.3) | | | | | | (0.6) | | | | | | (0.4) | | | | | | (1.2) | | | | | |  |
| **Patients below target, %** | 0.1% | | | | 57.7% | | | | 15.6% | | | | | | 26.5% | | | | | | 19.2% | | | | | | 0.0% | | | | | |  |
| **Dose (IU/kg per week)** Median (IQR) | 35.0 | | | | 182.0 | | | | 60.0 | | | | | | 77.8 | | | | | | 69.0 | | | | | | 10.0 | | | | | |  |
|  | (25.0–35.0) | | | | (142.0–182.0) | | | | (47.5–77.5) | | | | | | (70.0–87.5) | | | | | | (53.0–87.5) | | | | | | (10.0–10.0) | | | | | |  |
| Mean (SD) | 31.5 | | | | 161.9 | | | | 61.7 | | | | | | 78.4 | | | | | | 66.9 | | | | | | 10.4 | | | | | |  |
|  | (4.9) | | | | (29.4) | | | | (17.8) | | | | | | (10.5) | | | | | | (17.7) | | | | | | (1.4) | | | | | |  |
| **Patients among dose-intervals, %** | 7d | | 47.4% | | 3d | | 77.0% | | 7d | | | 100.0% | | | 8d | | | 48.7% | | | 7d | | | 68.8% | | | 7d | | | 100.0% | | |  |
|  | 10d | | 16.7% | | 3.5d | | 11.4% | |  | | |  | | | 9d | | | 20.5% | | | 8d | | | 15.4% | | |  | | |  | | |  |
|  | 14d | | 35.9% | | 4d | | 11.6% | |  | | |  | | | 10d | | | 13.9% | | | 9d | | | 6.2% | | |  | | |  | | |  |
|  |  | |  | |  | |  | |  | | |  | | | 11d | | | 8.5% | | | 10d | | | 4.3% | | |  | | |  | | |  |
|  |  | |  | |  | |  | |  | | |  | | | 12d | | | 4.4% | | | 11d | | | 2.8% | | |  | | |  | | |  |
|  |  | |  | |  | |  | |  | | |  | | | 13d | | | 4.1% | | | 12d | | | 1.3% | | |  | | |  | | |  |
|  |  | |  | |  | |  | |  | | |  | | | 14d | | | 0.0% | | | 13d | | | 1.2% | | |  | | |  | | |  |
|  |  | |  | |  | |  | |  | | |  | | | 15d | | | 0.0% | | | 14d | | | 0.0% | | |  | | |  | | |  |
|  |  | |  | |  | |  | |  | | |  | | | 16d | | | 0.0% | | | 15d | | | 0.0% | | |  | | |  | | |  |
|  |  | |  | |  | |  | |  | | |  | | | 17d | | | 0.0% | | | 16d | | | 0.0% | | |  | | |  | | |  |
|  |  | |  | |  | |  | |  | | |  | | | 18d | | | 0.0% | | | 17d | | | 0.0% | | |  | | |  | | |  |
|  |  | |  | |  | |  | |  | | |  | | | 19d | | | 0.0% | | | 18d | | | 0.0% | | |  | | |  | | |  |
|  |  | |  | |  | |  | |  | | |  | | | 20d | | | 0.0% | | | 19d | | | 0.0% | | |  | | |  | | |  |
|  |  | |  | |  | |  | |  | | |  | | | 21d | | | 0.0% | | | 20d | | | 0.0% | | |  | | |  | | |  |
|  |  | |  | |  | |  | |  | | |  | | |  | | |  | | | 21d | | | 0.0% | | |  | | |  | | |  |
| **Ratio between weekly dose (IU/kg) FIX product and rIX-FP** Median (IQR) |  | | | | 5.2 | | | | 1.9 | | | | | | 2.5 | | | | | | 2.2 | | | | | | 0.3 | | | | | |  |
|  |  | | | | (4.5–5.9) | | | | (1.5–2.5) | | | | | | (2.2–2.8) | | | | | | (1.7–2.5) | | | | | | (0.3–0.4) | | | | | |  |
| Mean (SD) |  | | | | 5.3 | | | | 2.0 | | | | | | 2.6 | | | | | | 2.2 | | | | | | 0.3 | | | | | |  |
|  |  | | | | (1.3) | | | | (0.7) | | | | | | (0.5) | | | | | | (0.7) | | | | | | (0.1) | | | | | |  |
| ^Ⱡ^Patients were randomly assigned to individualized interval prophylaxis from a Bernoulli distribution with a probability 0.315, based on data from clinical trial 998HB102 (NCT01027364). In the simulations underlying the results of this table, 68.8% of the simulated patients were assigned to receive weekly prophylaxis and 31.3% to individualized interval prophylaxis. | | | | | | | | | | | | | | | | | | | | | | | | | | | | | | | | |  |

| **Patients 12 to 17 \| Target 3%; Control 15%** |  |  |  |  |  |  |  |  |  |  |  |  |
| --- | --- | --- | --- | --- | --- | --- | --- | --- | --- | --- | --- | --- |
|  | **rIX-FP** | | **rFIX** | | **rFIXFc** weekly | | **rFIXFc** ind. Interval | | **rFIXFc^Ⱡ^** weekly and ind.interval | | **N9-GP** | |
| **Steady-state FIX trough levels** Median (IQR) | 9.7 | | 2.6 | | 3.0 | | 3.2 | | 3.0 | | 4.1 | |
|  | (7.5–12.4) | | (1.5–3.0) | | (3.0–3.0) | | (2.9–3.4) | | (3.0–3.0) | | (3.3–5.1) | |
| Mean (SD) | 9.8 | | 2.2 | | 2.9 | | 3.1 | | 3.0 | | 4.4 | |
|  | (3.1) | | (0.9) | | (0.3) | | (0.6) | | (0.4) | | (1.2) | |
| **Patients below target, %** | 0.1% | | 57.7% | | 15.6% | | 26.5% | | 19.2% | | 0.0% | |
| **Dose (IU/kg per week)** Median (IQR) | 35.0 | | 182.0 | | 60.0 | | 77.8 | | 69.0 | | 10.0 | |
|  | (35.0–35.0) | | (142.0–182.0) | | (47.5–77.5) | | (70.0–87.5) | | (53.0–87.5) | | (10.0–10.0) | |
| Mean (SD) | 34 | | 161.9 | | 61.7 | | 78.4 | | 66.9 | | 10.4 | |
|  | (3.2) | | (29.4) | | (17.8) | | (10.5) | | (17.7) | | (1.4) | |
| **Patients among dose-intervals, %** | 7d | 81.0% | 3d | 77.0% | 7d | 100.0% | 8d | 48.7% | 7d | 68.8% | 7d | 100.0% |
|  | 10d | 8.6% | 3.5d | 11.4% |  |  | 9d | 20.5% | 8d | 15.4% |  |  |
|  | 14d | 10.4% | 4d | 11.6% |  |  | 10d | 13.9% | 9d | 6.2% |  |  |
|  |  |  |  |  |  |  | 11d | 8.5% | 10d | 4.3% |  |  |
|  |  |  |  |  |  |  | 12d | 4.4% | 11d | 2.8% |  |  |
|  |  |  |  |  |  |  | 13d | 4.1% | 12d | 1.3% |  |  |
|  |  |  |  |  |  |  | 14d | 0.0% | 13d | 1.2% |  |  |
|  |  |  |  |  |  |  | 15d | 0.0% | 14d | 0.0% |  |  |
|  |  |  |  |  |  |  | 16d | 0.0% | 15d | 0.0% |  |  |
|  |  |  |  |  |  |  | 17d | 0.0% | 16d | 0.0% |  |  |
|  |  |  |  |  |  |  | 18d | 0.0% | 17d | 0.0% |  |  |
|  |  |  |  |  |  |  | 19d | 0.0% | 18d | 0.0% |  |  |
|  |  |  |  |  |  |  | 20d | 0.0% | 19d | 0.0% |  |  |
|  |  |  |  |  |  |  | 21d | 0.0% | 20d | 0.0% |  |  |
|  |  |  |  |  |  |  |  |  | 21d | 0.0% |  |  |
| **Ratio between weekly dose (IU/kg) FIX product and rIX-FP** Median (IQR) |  | | 5.2 | | 1.8 | | 2.5 | | 2.0 | | 0.3 | |
|  |  | | (4.2–5.2) | | (1.4–2.3) | | (2.0–2.5) | | (1.5–2.5) | | (0.3–0.3) | |
| Mean (SD) |  | | 4.8 | | 1.8 | | 2.3 | | 2.0 | | 0.3 | |
|  |  | | (1.0) | | (0.6) | | (0.4) | | (0.6) | | (0.1) | |
| ^Ⱡ^Patients were randomly assigned to individualized interval prophylaxis from a Bernoulli distribution with a probability 0.315, based on data from clinical trial 998HB102 (NCT01027364). In the simulations underlying the results of this table, 68.8% of the simulated patients were assigned to receive weekly prophylaxis and 31.3% to individualized interval prophylaxis. | | | | | | | | | | | | |

| **Patients 12 to 17 \| Target 3%; Control 20%** |  |  |  |  |  |  |  |  |  |  |  |  |
| --- | --- | --- | --- | --- | --- | --- | --- | --- | --- | --- | --- | --- |
|  | **rIX-FP** | | **rFIX** | | **rFIXFc** weekly | | **rFIXFc** ind. Interval | | **rFIXFc^Ⱡ^** weekly and ind.interval | | **N9-GP** | |
| **Steady-state FIX trough levels** Median (IQR) | 10.3 | | 2.6 | | 3.0 | | 3.2 | | 3.0 | | 4.1 | |
|  | (7.5–13.6) | | (1.5–3.0) | | (3.0–3.0) | | (2.9–3.4) | | (3.0–3.0) | | (3.3–5.1) | |
| Mean (SD) | 10.7 | | 2.2 | | 2.9 | | 3.1 | | 3.0 | | 4.4 | |
|  | (4.1) | | (0.9) | | (0.3) | | (0.6) | | (0.4) | | (1.2) | |
| **Patients below target, %** | 0.1% | | 57.7% | | 15.6% | | 26.5% | | 19.2% | | 0.0% | |
| **Dose (IU/kg per week)** Median (IQR) | 35.0 | | 182.0 | | 60.0 | | 77.8 | | 69.0 | | 10.0 | |
|  | (35.0–35.0) | | (142.0–182.0) | | (47.5–77.5) | | (70.0–87.5) | | (53.0–87.5) | | (10.0–10.0) | |
| Mean (SD) | 34.8 | | 161.9 | | 61.7 | | 78.4 | | 66.9 | | 10.4 | |
|  | (1.8) | | (29.4) | | (17.8) | | (10.5) | | (17.7) | | (1.4) | |
| **Patients among dose-intervals, %** | 7d | 94.7% | 3d | 77.0% | 7d | 100.0% | 8d | 48.7% | 7d | 68.8% | 7d | 100.0% |
|  | 10d | 2.6% | 3.5d | 11.4% |  |  | 9d | 20.5% | 8d | 15.4% |  |  |
|  | 14d | 2.7% | 4d | 11.6% |  |  | 10d | 13.9% | 9d | 6.2% |  |  |
|  |  |  |  |  |  |  | 11d | 8.5% | 10d | 4.3% |  |  |
|  |  |  |  |  |  |  | 12d | 4.4% | 11d | 2.8% |  |  |
|  |  |  |  |  |  |  | 13d | 4.1% | 12d | 1.3% |  |  |
|  |  |  |  |  |  |  | 14d | 0.0% | 13d | 1.2% |  |  |
|  |  |  |  |  |  |  | 15d | 0.0% | 14d | 0.0% |  |  |
|  |  |  |  |  |  |  | 16d | 0.0% | 15d | 0.0% |  |  |
|  |  |  |  |  |  |  | 17d | 0.0% | 16d | 0.0% |  |  |
|  |  |  |  |  |  |  | 18d | 0.0% | 17d | 0.0% |  |  |
|  |  |  |  |  |  |  | 19d | 0.0% | 18d | 0.0% |  |  |
|  |  |  |  |  |  |  | 20d | 0.0% | 19d | 0.0% |  |  |
|  |  |  |  |  |  |  | 21d | 0.0% | 20d | 0.0% |  |  |
|  |  |  |  |  |  |  |  |  | 21d | 0.0% |  |  |
| **Ratio between weekly dose (IU/kg) FIX product and rIX-FP** Median (IQR) |  | | 5.2 | | 1.7 | | 2.2 | | 2.0 | | 0.3 | |
|  |  | | (4.1–5.2) | | (1.4–2.2) | | (2.0–2.5) | | (1.5–2.5) | | (0.3–0.3) | |
| Mean (SD) |  | | 4.7 | | 1.8 | | 2.3 | | 1.9 | | 0.3 | |
|  |  | | (0.9) | | (0.5) | | (0.3) | | (0.5) | | (0.0) | |
| ^Ⱡ^Patients were randomly assigned to individualized interval prophylaxis from a Bernoulli distribution with a probability 0.315, based on data from clinical trial 998HB102 (NCT01027364). In the simulations underlying the results of this table, 68.8% of the simulated patients were assigned to receive weekly prophylaxis and 31.3% to individualized interval prophylaxis. | | | | | | | | | | | | |

| **Patients 12 to 17 \| Target 5%; Control 7.5%** | |  |  |  |  |  |  |  |  |  |  |  |
| --- | --- | --- | --- | --- | --- | --- | --- | --- | --- | --- | --- | --- |
|  | **rIX-FP** | | **rFIX** | | **rFIXFc** weekly | | **rFIXFc** ind. Interval | | **rFIXFc^Ⱡ^** weekly and ind.interval | | **N9-GP** | |
| **Steady-state FIX trough levels** Median (IQR) | 6.0 | | 2.6 | | 4.4 | | 3.9 | | 4.2 | | 5.1 | |
|  | (5.1–7.0) | | (1.5–4.2) | | (3.4–5.0) | | (2.9–5.0) | | (3.3–5.0) | | (5.1–5.2) | |
| Mean (SD) | 6.4 | | 2.8 | | 4.1 | | 3.9 | | 4.1 | | 5.3 | |
|  | (1.8) | | (1.5) | | (1.0) | | (1.2) | | (1.1) | | (0.6) | |
| **Patients below target, %** | 1.2% | | 83.0% | | 63.8% | | 75.3% | | 67.3% | | 0.0% | |
| **Dose (IU/kg per week)** Median (IQR) | 29.8 | | 182.0 | | 88.0 | | 87.5 | | 87.5 | | 12.5 | |
|  | (25.0–35.0) | | (182.0–182.0) | | (79.4–88.0) | | (87.5–87.5) | | (84.5–88.0) | | (10.0–15.5) | |
| Mean (SD) | 30.6 | | 176 | | 81.9 | | 86.1 | | 83.2 | | 13.5 | |
|  | (5.6) | | (15.9) | | (10.8) | | (4.5) | | (9.6) | | (4.1) | |
| **Patients among dose-intervals, %** | 7d | 25.0% | 3d | 93.2% | 7d | 100.0% | 8d | 89.9% | 7d | 68.8% | 7d | 100.0% |
|  | 10d | 17.3% | 3.5d | 4.4% |  |  | 9d | 6.2% | 8d | 28.2% |  |  |
|  | 14d | 57.7% | 4d | 2.4% |  |  | 10d | 2.8% | 9d | 1.8% |  |  |
|  |  |  |  |  |  |  | 11d | 1.0% | 10d | 0.9% |  |  |
|  |  |  |  |  |  |  | 12d | 0.2% | 11d | 0.2% |  |  |
|  |  |  |  |  |  |  | 13d | 0.0% | 12d | 0.1% |  |  |
|  |  |  |  |  |  |  | 14d | 0.0% | 13d | 0.0% |  |  |
|  |  |  |  |  |  |  | 15d | 0.0% | 14d | 0.0% |  |  |
|  |  |  |  |  |  |  | 16d | 0.0% | 15d | 0.0% |  |  |
|  |  |  |  |  |  |  | 17d | 0.0% | 16d | 0.0% |  |  |
|  |  |  |  |  |  |  | 18d | 0.0% | 17d | 0.0% |  |  |
|  |  |  |  |  |  |  | 19d | 0.0% | 18d | 0.0% |  |  |
|  |  |  |  |  |  |  | 20d | 0.0% | 19d | 0.0% |  |  |
|  |  |  |  |  |  |  | 21d | 0.0% | 20d | 0.0% |  |  |
|  |  |  |  |  |  |  |  |  | 21d | 0.0% |  |  |
| **Ratio between weekly dose (IU/kg) FIX product and rIX-FP** Median (IQR) |  | | 5.7 | | 2.5 | | 2.8 | | 2.7 | | 0.4 | |
|  |  | | (5.2–7.3) | | (2.5–3.4) | | (2.5–3.5) | | (2.5–3.5) | | (0.3–0.5) | |
| Mean (SD) |  | | 5.9 | | 2.8 | | 2.9 | | 2.8 | | 0.5 | |
|  |  | | (1.1) | | (0.6) | | (0.5) | | (0.6) | | (0.2) | |
| ^Ⱡ^Patients were randomly assigned to individualized interval prophylaxis from a Bernoulli distribution with a probability 0.315, based on data from clinical trial 998HB102 (NCT01027364). In the simulations underlying the results of this table, 68.8% of the simulated patients were assigned to receive weekly prophylaxis and 31.3% to individualized interval prophylaxis. | | | | | | | | | | | | |
| **Patients 12 to 17 \| Target 5%; Control 10%** | |  |  |  |  |  |  |  |  |  |  |  |
|  | **rIX-FP** | | **rFIX** | | **rFIXFc** weekly | | **rFIXFc** ind. Interval | | **rFIXFc^Ⱡ^** weekly and ind.interval | | **N9-GP** | |
| **Steady-state FIX trough levels** Median (IQR) | 7.7 | | 2.6 | | 4.4 | | 3.9 | | 4.2 | | 5.1 | |
|  | (6.0–9.0) | | (1.5–4.2) | | (3.4–5.0) | | (2.9–5.0) | | (3.3–5.0) | | (5.1–5.2) | |
| Mean (SD) | 7.6 | | 2.8 | | 4.1 | | 3.9 | | 4.1 | | 5.3 | |
|  | (2.0) | | (1.5) | | (1.0) | | (1.2) | | (1.1) | | (0.6) | |
| **Patients below target, %** | 1.2% | | 83.0% | | 63.8% | | 75.3% | | 67.3% | | 0.0% | |
| **Dose (IU/kg per week)** Median (IQR) | 35.0 | | 182.0 | | 88.0 | | 87.5 | | 87.5 | | 12.5 | |
|  | (25.0–35.0) | | (182.0–182.0) | | (79.4–88.0) | | (87.5–87.5) | | (84.5–88.0) | | (10.0–15.5) | |
| Mean (SD) | 31.9 | | 176 | | 81.9 | | 86.1 | | 83.2 | | 13.5 | |
|  | (5.7) | | (15.9) | | (10.8) | | (4.6) | | (9.6) | | (4.1) | |
| **Patients among dose-intervals, %** | 7d | 47.4% | 3d | 93.2% | 7d | 100.0% | 8d | 89.9% | 7d | 68.8% | 7d | 100.0% |
|  | 10d | 16.7% | 3.5d | 4.4% |  |  | 9d | 6.2% | 8d | 28.2% |  |  |
|  | 14d | 35.9% | 4d | 2.4% |  |  | 10d | 2.6% | 9d | 1.8% |  |  |
|  |  |  |  |  |  |  | 11d | 1.0% | 10d | 0.8% |  |  |
|  |  |  |  |  |  |  | 12d | 0.2% | 11d | 0.2% |  |  |
|  |  |  |  |  |  |  | 13d | 0.2% | 12d | 0.1% |  |  |
|  |  |  |  |  |  |  | 14d | 0.0% | 13d | 0.0% |  |  |
|  |  |  |  |  |  |  | 15d | 0.0% | 14d | 0.0% |  |  |
|  |  |  |  |  |  |  | 16d | 0.0% | 15d | 0.0% |  |  |
|  |  |  |  |  |  |  | 17d | 0.0% | 16d | 0.0% |  |  |
|  |  |  |  |  |  |  | 18d | 0.0% | 17d | 0.0% |  |  |
|  |  |  |  |  |  |  | 19d | 0.0% | 18d | 0.0% |  |  |
|  |  |  |  |  |  |  | 20d | 0.0% | 19d | 0.0% |  |  |
|  |  |  |  |  |  |  | 21d | 0.0% | 20d | 0.0% |  |  |
|  |  |  |  |  |  |  |  |  | 21d | 0.0% |  |  |
| **Ratio between weekly dose (IU/kg) FIX product and rIX-FP** Median (IQR) |  | | 5.2 | | 2.5 | | 2.5 | | 2.5 | | 0.4 | |
|  |  | | (5.2–7.3) | | (2.4–3.3) | | (2.5–3.5) | | (2.5–3.5) | | (0.3–0.5) | |
| Mean (SD) |  | | 5.7 | | 2.6 | | 2.8 | | 2.7 | | 0.4 | |
|  |  | | (1.2) | | (0.6) | | (0.5) | | (0.6) | | (0.2) | |
| ^Ⱡ^Patients were randomly assigned to individualized interval prophylaxis from a Bernoulli distribution with a probability 0.315, based on data from clinical trial 998HB102 (NCT01027364). In the simulations underlying the results of this table, 68.8% of the simulated patients were assigned to receive weekly prophylaxis and 31.3% to individualized interval prophylaxis. | | | | | | | | | | | | |

| **Patients 12 to 17 \| Target 5%; Control 15%** | | | | | | | |  |  |  |  |  |
| --- | --- | --- | --- | --- | --- | --- | --- | --- | --- | --- | --- | --- |
|  | **rIX-FP** | | **rFIX** | | **rFIXFc** weekly | | **rFIXFc** ind. Interval | | **rFIXFc^Ⱡ^** weekly and ind.interval | | **N9-GP** | |
| **Steady-state FIX trough levels** Median (IQR) | 9.7 | | 2.6 | | 4.4 | | 3.9 | | 4.2 | | 5.1 | |
|  | (7.5–12.4) | | (1.5–4.2) | | (3.4–5.0) | | (2.9–5.0) | | (3.3–5.0) | | (5.1–5.2) | |
| Mean (SD) | 9.8 | | 2.8 | | 4.1 | | 3.9 | | 4.1 | | 5.3 | |
|  | (3.0) | | (1.5) | | (1.0) | | (1.2) | | (1.1) | | (0.6) | |
| **Patients below target, %** | 1.2% | | 83.0% | | 63.8% | | 75.3% | | 67.3% | | 0.0% | |
| **Dose (IU/kg per week)** Median (IQR) | 35.0 | | 182.0 | | 88.0 | | 87.5 | | 87.5 | | 12.5 | |
|  | (35.0–35.0) | | (182.0–182.0) | | (79.4–88.0) | | (87.5–87.5) | | (84.5–88.0) | | (10.0–15.5) | |
| Mean (SD) | 34.5 | | 176 | | 81.9 | | 86.1 | | 83.2 | | 13.5 | |
|  | (4.0) | | (15.9) | | (10.8) | | (4.6) | | (9.6) | | (4.1) | |
| **Patients among dose-intervals, %** | 7d | 81.0% | 3d | 93.2% | 7d | 100.0% | 8d | 89.9% | 7d | 68.8% | 7d | 100.0% |
|  | 10d | 8.6% | 3.5d | 4.4% |  |  | 9d | 6.2% | 8d | 28.2% |  |  |
|  | 14d | 10.4% | 4d | 2.4% |  |  | 10d | 2.6% | 9d | 1.8% |  |  |
|  |  |  |  |  |  |  | 11d | 1.0% | 10d | 0.8% |  |  |
|  |  |  |  |  |  |  | 12d | 0.2% | 11d | 0.2% |  |  |
|  |  |  |  |  |  |  | 13d | 0.2% | 12d | 0.1% |  |  |
|  |  |  |  |  |  |  | 14d | 0.0% | 13d | 0.0% |  |  |
|  |  |  |  |  |  |  | 15d | 0.0% | 14d | 0.0% |  |  |
|  |  |  |  |  |  |  | 16d | 0.0% | 15d | 0.0% |  |  |
|  |  |  |  |  |  |  | 17d | 0.0% | 16d | 0.0% |  |  |
|  |  |  |  |  |  |  | 18d | 0.0% | 17d | 0.0% |  |  |
|  |  |  |  |  |  |  | 19d | 0.0% | 18d | 0.0% |  |  |
|  |  |  |  |  |  |  | 20d | 0.0% | 19d | 0.0% |  |  |
|  |  |  |  |  |  |  | 21d | 0.0% | 20d | 0.0% |  |  |
|  |  |  |  |  |  |  |  |  | 21d | 0.0% |  |  |
| **Ratio between weekly dose (IU/kg) FIX product and rIX-FP** Median (IQR) |  | | 5.2 | | 2.5 | | 2.5 | | 2.5 | | 0.4 | |
|  |  | | (5.2–5.2) | | (2.2–2.5) | | (2.5–2.5) | | (2.3–2.5) | | (0.3–0.5) | |
| Mean (SD) |  | | 5.2 | | 2.4 | | 2.5 | | 2.4 | | 0.4 | |
|  |  | | (0.8) | | (0.4) | | (0.4) | | (0.4) | | (0.1) | |
| ^Ⱡ^Patients were randomly assigned to individualized interval prophylaxis from a Bernoulli distribution with a probability 0.315, based on data from clinical trial 998HB102 (NCT01027364). In the simulations underlying the results of this table, 68.8% of the simulated patients were assigned to receive weekly prophylaxis and 31.3% to individualized interval prophylaxis. | | | | | | | | | | | | |

| **Patients 12 to 17 \| Target 5%; Control 20%** | | | | | | | | |  | | |  | | |  | | |  | | |  | | |  | | |  | | |  | | |  | | |  | | |
| --- | --- | --- | --- | --- | --- | --- | --- | --- | --- | --- | --- | --- | --- | --- | --- | --- | --- | --- | --- | --- | --- | --- | --- | --- | --- | --- | --- | --- | --- | --- | --- | --- | --- | --- | --- | --- | --- | --- |
|  | **rIX-FP** | | | | | | | | **rFIX** | | | | | | **rFIXFc** weekly | | | | | | **rFIXFc** ind. Interval | | | | | | **rFIXFc^Ⱡ^** weekly and ind.interval | | | | | | **N9-GP** | | | | | |
| **Steady-state FIX trough levels** Median (IQR) | 10.3 | | | | | | | | 2.6 | | | | | | 4.4 | | | | | | 3.9 | | | | | | 4.2 | | | | | | 5.1 | | | | | |
|  | (7.5–13.6) | | | | | | | | (1.5–4.2) | | | | | | (3.4–5.0) | | | | | | (2.9–5.0) | | | | | | (3.3–5.0) | | | | | | (5.1–5.2) | | | | | |
| Mean (SD) | 10.7 | | | | | | | | 2.8 | | | | | | 4.1 | | | | | | 3.9 | | | | | | 4.1 | | | | | | 5.3 | | | | | |
|  | (4.0) | | | | | | | | (1.5) | | | | | | (1.0) | | | | | | (1.2) | | | | | | (1.1) | | | | | | (0.6) | | | | | |
| **Patients below target, %** | 1.2% | | | | | | | | 83.0% | | | | | | 63.8% | | | | | | 75.3% | | | | | | 67.3% | | | | | | 0.0% | | | | | |
| **Dose (IU/kg per week)** Median (IQR) | 35.0 | | | | | | | | 182.0 | | | | | | 88.0 | | | | | | 87.5 | | | | | | 87.5 | | | | | | 12.5 | | | | | |
|  | (35.0–35.0) | | | | | | | | (182.0–182.0) | | | | | | (79.4–88.0) | | | | | | (87.5–87.5) | | | | | | (84.5–88.0) | | | | | | (10.0–15.5) | | | | | |
| Mean (SD) | 35.3 | | | | | | | | 176 | | | | | | 81.9 | | | | | | 86.1 | | | | | | 83.2 | | | | | | 13.5 | | | | | |
|  | (2.9) | | | | | | | | (15.9) | | | | | | (10.8) | | | | | | (4.6) | | | | | | (9.6) | | | | | | (4.1) | | | | | |
| **Patients among dose-intervals, %** | 7d | | | 94.7% | | | | | 3d | | | 93.2% | | | 7d | | | 100.0% | | | 8d | | | 89.9% | | | 7d | | | 68.8% | | | 7d | | | 100.0% | | |
|  | 10d | | | 2.6% | | | | | 3.5d | | | 4.4% | | |  | | |  | | | 9d | | | 6.2% | | | 8d | | | 28.2% | | |  | | |  | | |
|  | 14d | | | 2.7% | | | | | 4d | | | 2.4% | | |  | | |  | | | 10d | | | 2.6% | | | 9d | | | 1.8% | | |  | | |  | | |
|  |  | | |  | | | | |  | | |  | | |  | | |  | | | 11d | | | 1.0% | | | 10d | | | 0.8% | | |  | | |  | | |
|  |  | | |  | | | | |  | | |  | | |  | | |  | | | 12d | | | 0.2% | | | 11d | | | 0.2% | | |  | | |  | | |
|  |  | | |  | | | | |  | | |  | | |  | | |  | | | 13d | | | 0.2% | | | 12d | | | 0.1% | | |  | | |  | | |
|  |  | | |  | | | | |  | | |  | | |  | | |  | | | 14d | | | 0.0% | | | 13d | | | 0.0% | | |  | | |  | | |
|  |  | | |  | | | | |  | | |  | | |  | | |  | | | 15d | | | 0.0% | | | 14d | | | 0.0% | | |  | | |  | | |
|  |  | | |  | | | | |  | | |  | | |  | | |  | | | 16d | | | 0.0% | | | 15d | | | 0.0% | | |  | | |  | | |
|  |  | | |  | | | | |  | | |  | | |  | | |  | | | 17d | | | 0.0% | | | 16d | | | 0.0% | | |  | | |  | | |
|  |  | | |  | | | | |  | | |  | | |  | | |  | | | 18d | | | 0.0% | | | 17d | | | 0.0% | | |  | | |  | | |
|  |  | | |  | | | | |  | | |  | | |  | | |  | | | 19d | | | 0.0% | | | 18d | | | 0.0% | | |  | | |  | | |
|  |  | | |  | | | | |  | | |  | | |  | | |  | | | 20d | | | 0.0% | | | 19d | | | 0.0% | | |  | | |  | | |
|  |  | | |  | | | | |  | | |  | | |  | | |  | | | 21d | | | 0.0% | | | 20d | | | 0.0% | | |  | | |  | | |
|  |  | | |  | | | | |  | | |  | | |  | | |  | | |  | | |  | | | 21d | | | 0.0% | | |  | | |  | | |
| **Ratio between weekly dose (IU/kg) FIX product and rIX-FP** Median (IQR) |  | | | | | | | | 5.2 | | | | | | 2.5 | | | | | | 2.5 | | | | | | 2.5 | | | | | | 0.4 | | | | | |
|  |  | | | | | | | | (5.2–5.2) | | | | | | (2.2–2.5) | | | | | | (2.5–2.5) | | | | | | (2.3–2.5) | | | | | | (0.3–0.4) | | | | | |
| Mean (SD) |  | | | | | | | | 5.0 | | | | | | 2.3 | | | | | | 2.5 | | | | | | 2.4 | | | | | | 0.4 | | | | | |
|  |  | | | | | | | | (0.6) | | | | | | (0.4) | | | | | | (0.2) | | | | | | (0.3) | | | | | | (0.1) | | | | | |
| ^Ⱡ^Patients were randomly assigned to individualized interval prophylaxis from a Bernoulli distribution with a probability 0.315, based on data from clinical trial 998HB102 (NCT01027364). In the simulations underlying the results of this table, 68.8% of the simulated patients were assigned to receive weekly prophylaxis and 31.3% to individualized interval prophylaxis. | | | | | | | | | | | | | | | | | | | | | | | | | | | | | | | | | | | | | | |
| **Patients 12 to 17 \| Target 7.5%; Control 10%** | | | | | | | |  | | |  | | |  | | |  | | |  | | |  | | |  | | |  | | |  | | |  | | |  |
|  | | | **rIX-FP** | | | | | **rFIX** | | | | | | **rFIXFc** weekly | | | | | | **rFIXFc** ind. Interval | | | | | | **rFIXFc^Ⱡ^** weekly and ind.interval | | | | | | **N9-GP** | | | | | |  |
| **Steady-state FIX trough levels** Median (IQR) | | | 7.7 | | | | | 2.6 | | | | | | 4.4 | | | | | | 3.9 | | | | | | 4.2 | | | | | | 7.6 | | | | | |  |
|  |  |  | (7.5–9.0) | | | | | (1.5–4.2) | | | | | | (3.4–5.6) | | | | | | (2.9–5.0) | | | | | | (3.3–5.4) | | | | | | (7.5–7.7) | | | | | |  |
| Mean (SD) | | | 8.3 | | | | | 3.0 | | | | | | 4.6 | | | | | | 4.1 | | | | | | 4.4 | | | | | | 7.6 | | | | | |  |
|  |  |  | (1.4) | | | | | (2.0) | | | | | | (1.5) | | | | | | (1.6) | | | | | | (1.5) | | | | | | (0.1) | | | | | |  |
| **Patients below target, %** | | | 5.8% | | | | | 94.7% | | | | | | 93.9% | | | | | | 96.4% | | | | | | 94.7% | | | | | | 0.0% | | | | | |  |
| **Dose (IU/kg per week)** Median (IQR) | | | 35.0 | | | | | 182.0 | | | | | | 88.0 | | | | | | 87.5 | | | | | | 88.0 | | | | | | 18.5 | | | | | |  |
|  |  |  | (30.0–35.3) | | | | | (182.0–182.0) | | | | | | (88.0–88.0) | | | | | | (87.5–87.5) | | | | | | (87.5–88.0) | | | | | | (15.0–23.0) | | | | | |  |
| Mean (SD) | | | 34.5 | | | | | 180.6 | | | | | | 87.3 | | | | | | 87.4 | | | | | | 87.3 | | | | | | 19.7 | | | | | |  |
|  |  |  | (6.6) | | | | | (7.1) | | | | | | (3.5) | | | | | | (1.2) | | | | | | (2.9) | | | | | | (6.6) | | | | | |  |
| **Patients among dose-intervals, %** | | | 7d | | | 47.4% | | 3d | | | 98.7% | | | 7d | | | 100.0% | | | 8d | | | 99.1% | | | 7d | | | 68.8% | | | 7d | | | 100.0% | | |  |
|  |  |  | 10d | | | 16.7% | | 3.5d | | | 1.0% | | |  | | |  | | | 9d | | | 0.6% | | | 8d | | | 31.0% | | |  | | |  | | |  |
|  |  |  | 14d | | | 35.9% | | 4d | | | 0.3% | | |  | | |  | | | 10d | | | 0.2% | | | 9d | | | 0.2% | | |  | | |  | | |  |
|  |  |  |  | | |  | |  | | |  | | |  | | |  | | | 11d | | | 0.0% | | | 10d | | | 0.0% | | |  | | |  | | |  |
|  |  |  |  | | |  | |  | | |  | | |  | | |  | | | 12d | | | 0.0% | | | 11d | | | 0.0% | | |  | | |  | | |  |
|  |  |  |  | | |  | |  | | |  | | |  | | |  | | | 13d | | | 0.0% | | | 12d | | | 0.0% | | |  | | |  | | |  |
|  |  |  |  | | |  | |  | | |  | | |  | | |  | | | 14d | | | 0.0% | | | 13d | | | 0.0% | | |  | | |  | | |  |
|  |  |  |  | | |  | |  | | |  | | |  | | |  | | | 15d | | | 0.0% | | | 14d | | | 0.0% | | |  | | |  | | |  |
|  |  |  |  | | |  | |  | | |  | | |  | | |  | | | 16d | | | 0.0% | | | 15d | | | 0.0% | | |  | | |  | | |  |
|  |  |  |  | | |  | |  | | |  | | |  | | |  | | | 17d | | | 0.0% | | | 16d | | | 0.0% | | |  | | |  | | |  |
|  |  |  |  | | |  | |  | | |  | | |  | | |  | | | 18d | | | 0.0% | | | 17d | | | 0.0% | | |  | | |  | | |  |
|  |  |  |  | | |  | |  | | |  | | |  | | |  | | | 19d | | | 0.0% | | | 18d | | | 0.0% | | |  | | |  | | |  |
|  |  |  |  | | |  | |  | | |  | | |  | | |  | | | 20d | | | 0.0% | | | 19d | | | 0.0% | | |  | | |  | | |  |
|  |  |  |  | | |  | |  | | |  | | |  | | |  | | | 21d | | | 0.0% | | | 20d | | | 0.0% | | |  | | |  | | |  |
|  |  |  |  | | |  | |  | | |  | | |  | | |  | | |  | | |  | | | 21d | | | 0.0% | | |  | | |  | | |  |
| **Ratio between weekly dose (IU/kg) FIX product and rIX-FP** Median (IQR) | | |  | | | | | 5.2 | | | | | | 2.5 | | | | | | 2.5 | | | | | | 2.5 | | | | | | 0.5 | | | | | |  |
|  |  |  |  | | | | | (5.0–6.0) | | | | | | (2.4–2.9) | | | | | | (2.5–2.9) | | | | | | (2.4–2.9) | | | | | | (0.4–0.7) | | | | | |  |
| Mean (SD) | | |  | | | | | 5.4 | | | | | | 2.6 | | | | | | 2.6 | | | | | | 2.6 | | | | | | 0.6 | | | | | |  |
|  |  |  |  | | | | | (1.0) | | | | | | (0.5) | | | | | | (0.5) | | | | | | (0.5) | | | | | | (0.2) | | | | | |  |
| ^Ⱡ^Patients were randomly assigned to individualized interval prophylaxis from a Bernoulli distribution with a probability 0.315, based on data from clinical trial 998HB102 (NCT01027364). In the simulations underlying the results of this table, 68.8% of the simulated patients were assigned to receive weekly prophylaxis and 31.3% to individualized interval prophylaxis. | | | | | | | | | | | | | | | | | | | | | | | | | | | | | | | | | | | | | |  |
| **Patients 12 to 17 \| Target 7.5%; Control 15%** | | | | | | |  | | |  | | |  | | |  | | |  | | |  | | |  | | |  | | |  | | |  | | |  |  |
|  | | **rIX-FP** | | | | | **rFIX** | | | | | | **rFIXFc** weekly | | | | | | **rFIXFc** ind. Interval | | | | | | **rFIXFc^Ⱡ^** weekly and ind.interval | | | | | | **N9-GP** | | | | | |  |  |
| **Steady-state FIX trough levels** Median (IQR) | | 9.7 | | | | | 2.6 | | | | | | 4.4 | | | | | | 3.9 | | | | | | 4.2 | | | | | | 7.6 | | | | | |  |  |
|  |  | (7.6–12.4) | | | | | (1.5–4.2) | | | | | | (3.4–5.6) | | | | | | (2.9–5.0) | | | | | | (3.3–5.4) | | | | | | (7.5–7.7) | | | | | |  |  |
| Mean (SD) | | 10.1 | | | | | 3.0 | | | | | | 4.6 | | | | | | 4.1 | | | | | | 4.4 | | | | | | 7.6 | | | | | |  |  |
|  |  | (2.6) | | | | | (2.0) | | | | | | (1.5) | | | | | | (1.6) | | | | | | (1.5) | | | | | | (0.1) | | | | | |  |  |
| **Patients below target, %** | | 5.8% | | | | | 94.7% | | | | | | 93.9% | | | | | | 96.4% | | | | | | 94.7% | | | | | | 0.0% | | | | | |  |  |
| **Dose (IU/kg per week)** Median (IQR) | | 35.0 | | | | | 182.0 | | | | | | 88.0 | | | | | | 87.5 | | | | | | 88.0 | | | | | | 18.5 | | | | | |  |  |
|  |  | (35.0–35.0) | | | | | (182.0–182.0) | | | | | | (88.0–88.0) | | | | | | (87.5–87.5) | | | | | | (87.5–88.0) | | | | | | (15.0–23.0) | | | | | |  |  |
| Mean (SD) | | 36 | | | | | 180.6 | | | | | | 87.3 | | | | | | 87.4 | | | | | | 87.3 | | | | | | 19.7 | | | | | |  |  |
|  |  | (5.7) | | | | | (7.1) | | | | | | (3.5) | | | | | | (1.2) | | | | | | (2.9) | | | | | | (6.6) | | | | | |  |  |
| **Patients among dose-intervals, %** | | 7d | | | 81.0% | | 3d | | | 98.7% | | | 7d | | | 100.0% | | | 8d | | | 99.1% | | | 7d | | | 68.8% | | | 7d | | | 100.0% | | |  |  |
|  |  | 10d | | | 8.6% | | 3.5d | | | 1.0% | | |  | | |  | | | 9d | | | 0.6% | | | 8d | | | 31.0% | | |  | | |  | | |  |  |
|  |  | 14d | | | 10.4% | | 4d | | | 0.3% | | |  | | |  | | | 10d | | | 0.2% | | | 9d | | | 0.2% | | |  | | |  | | |  |  |
|  |  |  | | |  | |  | | |  | | |  | | |  | | | 11d | | | 0.0% | | | 10d | | | 0.0% | | |  | | |  | | |  |  |
|  |  |  | | |  | |  | | |  | | |  | | |  | | | 12d | | | 0.0% | | | 11d | | | 0.0% | | |  | | |  | | |  |  |
|  |  |  | | |  | |  | | |  | | |  | | |  | | | 13d | | | 0.0% | | | 12d | | | 0.0% | | |  | | |  | | |  |  |
|  |  |  | | |  | |  | | |  | | |  | | |  | | | 14d | | | 0.0% | | | 13d | | | 0.0% | | |  | | |  | | |  |  |
|  |  |  | | |  | |  | | |  | | |  | | |  | | | 15d | | | 0.0% | | | 14d | | | 0.0% | | |  | | |  | | |  |  |
|  |  |  | | |  | |  | | |  | | |  | | |  | | | 16d | | | 0.0% | | | 15d | | | 0.0% | | |  | | |  | | |  |  |
|  |  |  | | |  | |  | | |  | | |  | | |  | | | 17d | | | 0.0% | | | 16d | | | 0.0% | | |  | | |  | | |  |  |
|  |  |  | | |  | |  | | |  | | |  | | |  | | | 18d | | | 0.0% | | | 17d | | | 0.0% | | |  | | |  | | |  |  |
|  |  |  | | |  | |  | | |  | | |  | | |  | | | 19d | | | 0.0% | | | 18d | | | 0.0% | | |  | | |  | | |  |  |
|  |  |  | | |  | |  | | |  | | |  | | |  | | | 20d | | | 0.0% | | | 19d | | | 0.0% | | |  | | |  | | |  |  |
|  |  |  | | |  | |  | | |  | | |  | | |  | | | 21d | | | 0.0% | | | 20d | | | 0.0% | | |  | | |  | | |  |  |
|  |  |  | | |  | |  | | |  | | |  | | |  | | |  | | |  | | | 21d | | | 0.0% | | |  | | |  | | |  |  |
| **Ratio between weekly dose (IU/kg) FIX product and rIX-FP** Median (IQR) | |  | | | | | 5.2 | | | | | | 2.5 | | | | | | 2.5 | | | | | | 2.5 | | | | | | 0.5 | | | | | |  |  |
|  |  |  | | | | | (5.0–5.2) | | | | | | (2.4–2.5) | | | | | | (2.5–2.5) | | | | | | (2.4–2.5) | | | | | | (0.4–0.7) | | | | | |  |  |
| Mean (SD) | |  | | | | | 5.1 | | | | | | 2.5 | | | | | | 2.5 | | | | | | 2.5 | | | | | | 0.6 | | | | | |  |  |
|  |  |  | | | | | (0.9) | | | | | | (0.4) | | | | | | (0.4) | | | | | | (0.4) | | | | | | (0.2) | | | | | |  |  |
| ^Ⱡ^Patients were randomly assigned to individualized interval prophylaxis from a Bernoulli distribution with a probability 0.315, based on data from clinical trial 998HB102 (NCT01027364). In the simulations underlying the results of this table, 68.8% of the simulated patients were assigned to receive weekly prophylaxis and 31.3% to individualized interval prophylaxis. | | | | | | | | | | | | | | | | | | | | | | | | | | | | | | | | | | | | |  |  |

| **Patients 12 to 17 \| Target 7.5%; Control 20%** | |  |  |  |  |  |  |  |  |  |  |  |
| --- | --- | --- | --- | --- | --- | --- | --- | --- | --- | --- | --- | --- |
|  | **rIX-FP** | | **rFIX** | | **rFIXFc** weekly | | **rFIXFc** ind. Interval | | **rFIXFc^Ⱡ^** weekly and ind.interval | | **N9-GP** | |
| **Steady-state FIX trough levels** Median (IQR) | 10.3 | | 2.6 | | 4.4 | | 3.9 | | 4.2 | | 7.6 | |
|  | (7.6–13.6) | | (1.5–4.2) | | (3.4–5.6) | | (2.9–5.0) | | (3.3–5.4) | | (7.5–7.7) | |
| Mean (SD) | 11.1 | | 3.0 | | 4.6 | | 4.1 | | 4.4 | | 7.6 | |
|  | (3.6) | | (2.0) | | (1.5) | | (1.6) | | (1.5) | | (0.1) | |
| **Patients below target, %** | 5.8% | | 94.7% | | 93.9% | | 96.4% | | 94.7% | | 0.0% | |
| **Dose (IU/kg per week)** Median (IQR) | 35.0 | | 182.0 | | 88.0 | | 87.5 | | 88.0 | | 18.5 | |
|  | (35.0–35.0) | | (182.0–182.0) | | (88.0–88.0) | | (87.5–87.5) | | (87.5–88.0) | | (15.0–23.0) | |
| Mean (SD) | 36.8 | | 180.6 | | 87.3 | | 87.4 | | 87.3 | | 19.7 | |
|  | (4.8) | | (7.1) | | (3.5) | | (1.2) | | (2.9) | | (6.6) | |
| **Patients among dose-intervals, %** | 7d | 94.7% | 3d | 98.7% | 7d | 100.0% | 8d | 99.1% | 7d | 68.8% | 7d | 100.0% |
|  | 10d | 2.6% | 3.5d | 1.0% |  |  | 9d | 0.6% | 8d | 31.0% |  |  |
|  | 14d | 2.7% | 4d | 0.3% |  |  | 10d | 0.2% | 9d | 0.2% |  |  |
|  |  |  |  |  |  |  | 11d | 0.0% | 10d | 0.0% |  |  |
|  |  |  |  |  |  |  | 12d | 0.0% | 11d | 0.0% |  |  |
|  |  |  |  |  |  |  | 13d | 0.0% | 12d | 0.0% |  |  |
|  |  |  |  |  |  |  | 14d | 0.0% | 13d | 0.0% |  |  |
|  |  |  |  |  |  |  | 15d | 0.0% | 14d | 0.0% |  |  |
|  |  |  |  |  |  |  | 16d | 0.0% | 15d | 0.0% |  |  |
|  |  |  |  |  |  |  | 17d | 0.0% | 16d | 0.0% |  |  |
|  |  |  |  |  |  |  | 18d | 0.0% | 17d | 0.0% |  |  |
|  |  |  |  |  |  |  | 19d | 0.0% | 18d | 0.0% |  |  |
|  |  |  |  |  |  |  | 20d | 0.0% | 19d | 0.0% |  |  |
|  |  |  |  |  |  |  | 21d | 0.0% | 20d | 0.0% |  |  |
|  |  |  |  |  |  |  |  |  | 21d | 0.0% |  |  |
| **Ratio between weekly dose (IU/kg) FIX product and rIX-FP** Median (IQR) |  | | 5.2 | | 2.5 | | 2.5 | | 2.5 | | 0.5 | |
|  |  | | (5.0–5.2) | | (2.4–2.5) | | (2.5–2.5) | | (2.4–2.5) | | (0.4–0.6) | |
| Mean (SD) |  | | 5.0 | | 2.4 | | 2.4 | | 2.4 | | 0.5 | |
|  |  | | (0.6) | | (0.3) | | (0.3) | | (0.3) | | (0.2) | |
| ^Ⱡ^Patients were randomly assigned to individualized interval prophylaxis from a Bernoulli distribution with a probability 0.315, based on data from clinical trial 998HB102 (NCT01027364). In the simulations underlying the results of this table, 68.8% of the simulated patients were assigned to receive weekly prophylaxis and 31.3% to individualized interval prophylaxis. | | | | | | | | | | | | |

| **Patients 12 to 17 \| Target 10.0%; Control 15%** | | | | | |  | |  | |  | |  | |  | |  | |  | |  | |  | |  | |
| --- | --- | --- | --- | --- | --- | --- | --- | --- | --- | --- | --- | --- | --- | --- | --- | --- | --- | --- | --- | --- | --- | --- | --- | --- | --- |
|  | | **rIX-FP** | | | | **rFIX** | | | | **rFIXFc** weekly | | | | **rFIXFc** ind. Interval | | | | **rFIXFc^Ⱡ^** weekly and ind.interval | | | | **N9-GP** | | | |
| **Steady-state FIX trough levels** Median (IQR) | | 10.1 | | | | 2.6 | | | | 4.4 | | | | 3.9 | | | | 4.2 | | | | 10.1 | | | |
|  |  | (10.0–12.4) | | | | (1.5–4.2) | | | | (3.4–5.6) | | | | (2.9–5.0) | | | | (3.3–5.4) | | | | (10.0–10.2) | | | |
| Mean (SD) | | 10.9 | | | | 3.1 | | | | 4.6 | | | | 4.1 | | | | 4.5 | | | | 10.1 | | | |
|  |  | (2.1) | | | | (2.2) | | | | (1.7) | | | | (1.6) | | | | (1.7) | | | | (0.1) | | | |
| **Patients below target, %** | | 15.3% | | | | 98.7% | | | | 99.2% | | | | 99.6% | | | | 99.4% | | | | 0.0% | | | |
| **Dose (IU/kg per week)** Median (IQR) | | 35.0 | | | | 182.0 | | | | 88.0 | | | | 87.5 | | | | 88.0 | | | | 24.5 | | | |
|  |  | (35.0–44.0) | | | | (182.0–182.0) | | | | (88.0–88.0) | | | | (87.5–87.5) | | | | (87.5–88.0) | | | | (20.0–30.5) | | | |
| Mean (SD) | | 38.5 | | | | 181.7 | | | | 87.9 | | | | 87.5 | | | | 87.8 | | | | 26.1 | | | |
|  |  | (7.0) | | | | (3.0) | | | | (1.0) | | | | (0.2) | | | | (0.8) | | | | (8.8) | | | |
| **Patients among dose-intervals, %** | | 7d | | 81.0% | | 3d | | 99.7% | | 7d | | 100.0% | | 8d | | 100.0% | | 7d | | 68.8% | | 7d | | 100.0% | |
|  |  | 10d | | 8.6% | | 3.5d | | 0.2% | |  | |  | | 9d | | 0.0% | | 8d | | 31.2% | |  | |  | |
|  |  | 14d | | 10.4% | | 4d | | 0.1% | |  | |  | | 10d | | 0.0% | | 9d | | 0.0% | |  | |  | |
|  |  |  | |  | |  | |  | |  | |  | | 11d | | 0.0% | | 10d | | 0.0% | |  | |  | |
|  |  |  | |  | |  | |  | |  | |  | | 12d | | 0.0% | | 11d | | 0.0% | |  | |  | |
|  |  |  | |  | |  | |  | |  | |  | | 13d | | 0.0% | | 12d | | 0.0% | |  | |  | |
|  |  |  | |  | |  | |  | |  | |  | | 14d | | 0.0% | | 13d | | 0.0% | |  | |  | |
|  |  |  | |  | |  | |  | |  | |  | | 15d | | 0.0% | | 14d | | 0.0% | |  | |  | |
|  |  |  | |  | |  | |  | |  | |  | | 16d | | 0.0% | | 15d | | 0.0% | |  | |  | |
|  |  |  | |  | |  | |  | |  | |  | | 17d | | 0.0% | | 16d | | 0.0% | |  | |  | |
|  |  |  | |  | |  | |  | |  | |  | | 18d | | 0.0% | | 17d | | 0.0% | |  | |  | |
|  |  |  | |  | |  | |  | |  | |  | | 19d | | 0.0% | | 18d | | 0.0% | |  | |  | |
|  |  |  | |  | |  | |  | |  | |  | | 20d | | 0.0% | | 19d | | 0.0% | |  | |  | |
|  |  |  | |  | |  | |  | |  | |  | | 21d | | 0.0% | | 20d | | 0.0% | |  | |  | |
|  |  |  | |  | |  | |  | |  | |  | |  | |  | | 21d | | 0.0% | |  | |  | |
| **Ratio between weekly dose (IU/kg) FIX product and rIX-FP** Median (IQR) | |  | | | | 5.2 | | | | 2.5 | | | | 2.5 | | | | 2.5 | | | | 0.7 | | | |
|  |  |  | | | | (4.1–5.2) | | | | (2.0–2.5) | | | | (2.0–2.5) | | | | (2.0–2.5) | | | | (0.5–0.8) | | | |
| Mean (SD) | |  | | | | 4.9 | | | | 2.4 | | | | 2.4 | | | | 2.4 | | | | 0.7 | | | |
|  |  |  | | | | (0.9) | | | | (0.4) | | | | (0.4) | | | | (0.4) | | | | (0.3) | | | |
| ^Ⱡ^Patients were randomly assigned to individualized interval prophylaxis from a Bernoulli distribution with a probability 0.315, based on data from clinical trial 998HB102 (NCT01027364). In the simulations underlying the results of this table, 68.8% of the simulated patients were assigned to receive weekly prophylaxis and 31.3% to individualized interval prophylaxis. | | | | | | | | | | | | | | | | | | | | | | | | | |
| **Patients 12 to 17 \| Target 10.0%; Control 20%** | | |  | |  | |  | |  | |  | |  | |  | |  | |  | |  | |  | |  |
|  | **rIX-FP** | | | | **rFIX** | | | | **rFIXFc** weekly | | | | **rFIXFc** ind. Interval | | | | **rFIXFc^Ⱡ^** weekly and ind.interval | | | | **N9-GP** | | | |  |
| **Steady-state FIX trough levels** Median (IQR) | 10.3 | | | | 2.6 | | | | 4.4 | | | | 3.9 | | | | 4.2 | | | | 10.1 | | | |  |
|  | (10.0–13.6) | | | | (1.5–4.2) | | | | (3.4–5.6) | | | | (2.9–5.0) | | | | (3.3–5.4) | | | | (10.0–10.2) | | | |  |
| **Steady-state FIX trough levels Mean (SD)** | 11.7 | | | | 3.1 | | | | 4.6 | | | | 4.1 | | | | 4.5 | | | | 10.1 | | | |  |
|  | (3.2) | | | | (2.2) | | | | (1.7) | | | | (1.6) | | | | (1.7) | | | | (0.1) | | | |  |
| **Patients below target** | 15.3% | | | | 98.7% | | | | 99.2% | | | | 99.6% | | | | 99.4% | | | | 0.0% | | | |  |
| **Dose (IU/kg per week)** Median (IQR) | 35.0 | | | | 182.0 | | | | 88.0 | | | | 87.5 | | | | 88.0 | | | | 24.5 | | | |  |
|  | (35.0–44.0) | | | | (182.0–182.0) | | | | (88.0–88.0) | | | | (87.5–87.5) | | | | (87.5–88.0) | | | | (20.0–30.5) | | | |  |
| **Dose (IU/kg per week) Mean (SD)** | 39.1 | | | | 181.7 | | | | 87.9 | | | | 87.5 | | | | 87.8 | | | | 26.1 | | | |  |
|  | (6.3) | | | | (3.0) | | | | (1.0) | | | | (0.2) | | | | (0.8) | | | | (8.8) | | | |  |
| **Patients among dose-intervals** | 7d | | 94.7% | | 3d | | 99.7% | | 7d | | 100.0% | | 8d | | 100.0% | | 7d | | 68.8% | | 7d | | 100.0% | |  |
|  | 10d | | 2.6% | | 3.5d | | 0.2% | |  | |  | | 9d | | 0.0% | | 8d | | 31.2% | |  | |  | |  |
|  | 14d | | 2.7% | | 4d | | 0.1% | |  | |  | | 10d | | 0.0% | | 9d | | 0.0% | |  | |  | |  |
|  |  | |  | |  | |  | |  | |  | | 11d | | 0.0% | | 10d | | 0.0% | |  | |  | |  |
|  |  | |  | |  | |  | |  | |  | | 12d | | 0.0% | | 11d | | 0.0% | |  | |  | |  |
|  |  | |  | |  | |  | |  | |  | | 13d | | 0.0% | | 12d | | 0.0% | |  | |  | |  |
|  |  | |  | |  | |  | |  | |  | | 14d | | 0.0% | | 13d | | 0.0% | |  | |  | |  |
|  |  | |  | |  | |  | |  | |  | | 15d | | 0.0% | | 14d | | 0.0% | |  | |  | |  |
|  |  | |  | |  | |  | |  | |  | | 16d | | 0.0% | | 15d | | 0.0% | |  | |  | |  |
|  |  | |  | |  | |  | |  | |  | | 17d | | 0.0% | | 16d | | 0.0% | |  | |  | |  |
|  |  | |  | |  | |  | |  | |  | | 18d | | 0.0% | | 17d | | 0.0% | |  | |  | |  |
|  |  | |  | |  | |  | |  | |  | | 19d | | 0.0% | | 18d | | 0.0% | |  | |  | |  |
|  |  | |  | |  | |  | |  | |  | | 20d | | 0.0% | | 19d | | 0.0% | |  | |  | |  |
|  |  | |  | |  | |  | |  | |  | | 21d | | 0.0% | | 20d | | 0.0% | |  | |  | |  |
|  |  | |  | |  | |  | |  | |  | |  | |  | | 21d | | 0.0% | |  | |  | |  |
| **Ratio between weekly dose (IU/kg) FIX product and rIX-FP** Median (IQR) |  | | | | 5.2 | | | | 2.5 | | | | 2.5 | | | | 2.5 | | | | 0.6 | | | |  |
|  |  | | | | (4.1–5.2) | | | | (2.0–2.5) | | | | (2.0–2.5) | | | | (2.0–2.5) | | | | (0.5–0.8) | | | |  |
| **Ratio between weekly dose (IU/kg) FIX product and rIX-FP Mean (SD)** |  | | | | 4.8 | | | | 2.3 | | | | 2.3 | | | | 2.3 | | | | 0.7 | | | |  |
|  |  | | | | (0.7) | | | | (0.4) | | | | (0.4) | | | | (0.4) | | | | (0.3) | | | |  |
| ^Ⱡ^Patients were randomly assigned to individualized interval prophylaxis from a Bernoulli distribution with a probability 0.315, based on data from clinical trial 998HB102 (NCT01027364). In the simulations underlying the results of this table, 68.8% of the simulated patients were assigned to receive weekly prophylaxis and 31.3% to individualized interval prophylaxis. | | | | | | | | | | | | | | | | | | | | | | | | |  |
| **Patients 12 to 17 \| Target 15%; Control 20%** | | |  | |  | |  | |  | |  | |  | |  | |  | |  | |  | |  | |  |
|  | **rIX-FP** | | | | **rFIX** | | | | **rFIXFc** weekly | | | | **rFIXFc** ind. Interval | | | | **rFIXFc^Ⱡ^** weekly and ind.interval | | | | **N9-GP** | | | |  |
| **Steady-state FIX trough levels** Median (IQR) | 15.0 | | | | 2.6 | | | | 4.4 | | | | 3.9 | | | | 4.2 | | | | 15.1 | | | |  |
|  | (11.9–15.2) | | | | (1.5–4.2) | | | | (3.4–5.6) | | | | (2.9–5.0) | | | | (3.3–5.4) | | | | (15.0–15.2) | | | |  |
| **Steady-state FIX trough levels Mean (SD)** | 13.6 | | | | 3.1 | | | | 4.6 | | | | 4.1 | | | | 4.5 | | | | 15.1 | | | |  |
|  | (3.2) | | | | (2.2) | | | | (1.7) | | | | (1.6) | | | | (1.7) | | | | (0.1) | | | |  |
| **Patients below target** | 43.6% | | | | 99.9% | | | | 100.0% | | | | 100.0% | | | | 100.0% | | | | 0.0% | | | |  |
| **Dose (IU/kg per week)** Median (IQR) | 47.5 | | | | 182.0 | | | | 88.0 | | | | 87.5 | | | | 88.0 | | | | 36.5 | | | |  |
|  | (37.5–50.0) | | | | (182.0–182.0) | | | | (88.0–88.0) | | | | (87.5–87.5) | | | | (87.5–88.0) | | | | (30.0–45.5) | | | |  |
| **Dose (IU/kg per week) Mean (SD)** | 44.2 | | | | 182 | | | | 88 | | | | 87.5 | | | | 87.8 | | | | 39 | | | |  |
|  | (6.6) | | | | (0.6) | | | | (0.0) | | | | (0.0) | | | | (0.2) | | | | (13.3) | | | |  |
| **Patients among dose-intervals** | 7d | | 94.7% | | 3d | | 100.0% | | 7d | | 100.0% | | 8d | | 100.0% | | 7d | | 68.8% | | 7d | | 100.0% | |  |
|  | 10d | | 2.6% | | 3.5d | | 0.0% | |  | |  | | 9d | | 0.0% | | 8d | | 31.2% | |  | |  | |  |
|  | 14d | | 2.7% | | 4d | | 0.0% | |  | |  | | 10d | | 0.0% | | 9d | | 0.0% | |  | |  | |  |
|  |  | |  | |  | |  | |  | |  | | 11d | | 0.0% | | 10d | | 0.0% | |  | |  | |  |
|  |  | |  | |  | |  | |  | |  | | 12d | | 0.0% | | 11d | | 0.0% | |  | |  | |  |
|  |  | |  | |  | |  | |  | |  | | 13d | | 0.0% | | 12d | | 0.0% | |  | |  | |  |
|  |  | |  | |  | |  | |  | |  | | 14d | | 0.0% | | 13d | | 0.0% | |  | |  | |  |
|  |  | |  | |  | |  | |  | |  | | 15d | | 0.0% | | 14d | | 0.0% | |  | |  | |  |
|  |  | |  | |  | |  | |  | |  | | 16d | | 0.0% | | 15d | | 0.0% | |  | |  | |  |
|  |  | |  | |  | |  | |  | |  | | 17d | | 0.0% | | 16d | | 0.0% | |  | |  | |  |
|  |  | |  | |  | |  | |  | |  | | 18d | | 0.0% | | 17d | | 0.0% | |  | |  | |  |
|  |  | |  | |  | |  | |  | |  | | 19d | | 0.0% | | 18d | | 0.0% | |  | |  | |  |
|  |  | |  | |  | |  | |  | |  | | 20d | | 0.0% | | 19d | | 0.0% | |  | |  | |  |
|  |  | |  | |  | |  | |  | |  | | 21d | | 0.0% | | 20d | | 0.0% | |  | |  | |  |
|  |  | |  | |  | |  | |  | |  | |  | |  | | 21d | | 0.0% | |  | |  | |  |
| **Ratio between weekly dose (IU/kg) FIX product and rIX-FP** Median (IQR) |  | | | | 3.8 | | | | 1.9 | | | | 1.8 | | | | 1.9 | | | | 0.8 | | | |  |
|  |  | | | | (3.6–4.9) | | | | (1.8–2.3) | | | | (1.8–2.3) | | | | (1.8–2.3) | | | | (0.7–1.1) | | | |  |
| **Ratio between weekly dose (IU/kg) FIX product and rIX-FP Mean (SD)** |  | | | | 4.2 | | | | 2.0 | | | | 2.0 | | | | 2.0 | | | | 0.9 | | | |  |
|  |  | | | | (0.7) | | | | (0.4) | | | | (0.4) | | | | (0.4) | | | | (0.3) | | | |  |
| ^Ⱡ^Patients were randomly assigned to individualized interval prophylaxis from a Bernoulli distribution with a probability 0.315, based on data from clinical trial 998HB102 (NCT01027364). In the simulations underlying the results of this table, 68.8% of the simulated patients were assigned to receive weekly prophylaxis and 31.3% to individualized interval prophylaxis. | | | | | | | | | | | | | | | | | | | | | | | | |  |

***Adults – Ideal posology***

| **Weight** |  |  |
| --- | --- | --- |
| Mean (SD) | 73.3 (15.4) | |

| **Patients ≥18 \| Target 1%; Control 5%** |  |  |  |  |  |  |  |  |  |  |  |  |
| --- | --- | --- | --- | --- | --- | --- | --- | --- | --- | --- | --- | --- |
|  | **rIX-FP** | | **rFIX** | | **rFIXFc** weekly | | **rFIXFc** ind. Interval | | **rFIXFc^Ⱡ^** weekly and ind.interval | | **N9-GP** | |
| **Steady-state FIX trough levels** Median (IQR) | 4.8 | | 1.0 | | 1.0 | | 1.3 | | 1.1 | | 4.1 | |
|  | (3.6–7.2) | | (1.0–1.0) | | (1.0–1.1) | | (1.2–1.8) | | (1.0–1.3) | | (3.4–5.1) | |
| Mean (SD) | 5.9 | | 1.0 | | 1.1 | | 1.6 | | 1.3 | | 4.3 | |
|  | (3.4) | | (0.1) | | (0.2) | | (0.6) | | (0.4) | | (1.3) | |
| **Patients below target, %** | 0.0% | | 11.3% | | 0.0% | | 0.2% | | 0.1% | | 0.0% | |
| **Dose (IU/kg per week)** Median (IQR) | 33.3 | | 107.0 | | 19.5 | | 53.8 | | 24.5 | | 10.0 | |
|  | (25.0–33.3) | | (63.0–134.2) | | (17.0–25.0) | | (53.8–58.3) | | (17.0–53.8) | | (10.0–10.0) | |
| Mean (SD) | 29.9 | | 103.7 | | 22.4 | | 57.9 | | 33.5 | | 10 | |
|  | (4.3) | | (46.4) | | (7.5) | | (7.5) | | (18.2) | | (0.0) | |
| **Patients among dose-intervals, %** | 7d | 4.3% | 3d | 24.7% | 7d | 100.0% | 8d | 1.5% | 7d | 68.6% | 7d | 100.0% |
|  | 10d | 7.3% | 3.5d | 17.5% |  |  | 9d | 4.1% | 8d | 0.5% |  |  |
|  | 14d | 43.4% | 4d | 57.7% |  |  | 10d | 7.0% | 9d | 1.3% |  |  |
|  | 21d | 45.1% |  |  |  |  | 11d | 10.9% | 10d | 2.3% |  |  |
|  |  |  |  |  |  |  | 12d | 12.6% | 11d | 3.5% |  |  |
|  |  |  |  |  |  |  | 13d | 59.0% | 12d | 4.1% |  |  |
|  |  |  |  |  |  |  | 14d | 4.8% | 13d | 18.2% |  |  |
|  |  |  |  |  |  |  | 15d | 0.0% | 14d | 1.5% |  |  |
|  |  |  |  |  |  |  | 16d | 0.0% | 15d | 0.0% |  |  |
|  |  |  |  |  |  |  | 17d | 0.0% | 16d | 0.0% |  |  |
|  |  |  |  |  |  |  | 18d | 0.0% | 17d | 0.0% |  |  |
|  |  |  |  |  |  |  | 19d | 0.0% | 18d | 0.0% |  |  |
|  |  |  |  |  |  |  | 20d | 0.0% | 19d | 0.0% |  |  |
|  |  |  |  |  |  |  | 21d | 0.0% | 20d | 0.0% |  |  |
|  |  |  |  |  |  |  |  |  | 21d | 0.0% |  |  |
| **Ratio between weekly dose (IU/kg) FIX product and rIX-FP** Median (IQR) |  | | 3.5 | | 0.7 | | 2.0 | | 0.8 | | 0.3 | |
|  |  | | (2.1–4.7) | | (0.5–0.9) | | (1.6–2.2) | | (0.7–1.6) | | (0.3–0.4) | |
| Mean (SD) |  | | 3.5 | | 0.8 | | 2.0 | | 1.1 | | 0.3 | |
|  |  | | (1.7) | | (0.3) | | (0.4) | | (0.7) | | (0.1) | |
| ^Ⱡ^Patients were randomly assigned to individualized interval prophylaxis from a Bernoulli distribution with a probability 0.315, based on data from clinical trial 998HB102 (NCT01027364). In the simulations underlying the results of this table, 68.6% of the simulated patients were assigned to receive weekly prophylaxis and 31.4% to individualized interval prophylaxis. | | | | | | | | | | | | |

| **Patients ≥18 \| Target 1%; Control 7.5%** |  |  |  |  |  |  |  |  |  |  |  |  |
| --- | --- | --- | --- | --- | --- | --- | --- | --- | --- | --- | --- | --- |
|  | **rIX-FP** | | **rFIX** | | **rFIXFc** weekly | | **rFIXFc** ind. Interval | | **rFIXFc^Ⱡ^** weekly and ind.interval | | **N9-GP** | |
| **Steady-state FIX trough levels** Median (IQR) | 6.2 | | 1.0 | | 1.0 | | 1.3 | | 1.1 | | 4.1 | |
|  | (4.8–7.3) | | (1.0–1.0) | | (1.0–1.1) | | (1.2–1.8) | | (1.0–1.3) | | (3.4–5.1) | |
| Mean (SD) | 6.8 | | 1.0 | | 1.1 | | 1.6 | | 1.3 | | 4.3 | |
|  | (2.9) | | (0.1) | | (0.2) | | (0.7) | | (0.5) | | (1.3) | |
| **Patients below target, %** | 0.0% | | 11.3% | | 0.0% | | 0.2% | | 0.1% | | 0.0% | |
| **Dose (IU/kg per week)** Median (IQR) | 33.3 | | 107.0 | | 19.5 | | 53.8 | | 24.5 | | 10.0 | |
|  | (25.0–35.0) | | (63.0–134.2) | | (17.0–25.0) | | (53.8–58.3) | | (17.0–53.8) | | (10.0–10.0) | |
| Mean (SD) | 29.9 | | 103.7 | | 22.4 | | 58.1 | | 33.6 | | 10 | |
|  | (4.7) | | (46.4) | | (7.5) | | (7.3) | | (18.2) | | (0.0) | |
| **Patients among dose-intervals, %** | 7d | 16.9% | 3d | 24.7% | 7d | 100.0% | 8d | 1.5% | 7d | 68.6% | 7d | 100.0% |
|  | 10d | 14.8% | 3.5d | 17.5% |  |  | 9d | 4.1% | 8d | 0.5% |  |  |
|  | 14d | 47.9% | 4d | 57.7% |  |  | 10d | 7.0% | 9d | 1.3% |  |  |
|  | 21d | 20.4% |  |  |  |  | 11d | 10.9% | 10d | 2.3% |  |  |
|  |  |  |  |  |  |  | 12d | 12.6% | 11d | 3.5% |  |  |
|  |  |  |  |  |  |  | 13d | 63.6% | 12d | 4.1% |  |  |
|  |  |  |  |  |  |  | 14d | 0.3% | 13d | 19.6% |  |  |
|  |  |  |  |  |  |  | 15d | 0.0% | 14d | 0.1% |  |  |
|  |  |  |  |  |  |  | 16d | 0.0% | 15d | 0.0% |  |  |
|  |  |  |  |  |  |  | 17d | 0.0% | 16d | 0.0% |  |  |
|  |  |  |  |  |  |  | 18d | 0.0% | 17d | 0.0% |  |  |
|  |  |  |  |  |  |  | 19d | 0.0% | 18d | 0.0% |  |  |
|  |  |  |  |  |  |  | 20d | 0.0% | 19d | 0.0% |  |  |
|  |  |  |  |  |  |  | 21d | 0.0% | 20d | 0.0% |  |  |
|  |  |  |  |  |  |  |  |  | 21d | 0.0% |  |  |
| **Ratio between weekly dose (IU/kg) FIX product and rIX-FP** Median (IQR) |  | | 3.5 | | 0.7 | | 2.2 | | 0.8 | | 0.3 | |
|  |  | | (2.1–4.8) | | (0.6–0.9) | | (1.6–2.2) | | (0.7–1.6) | | (0.3–0.4) | |
| Mean (SD) |  | | 3.6 | | 0.8 | | 2.0 | | 1.2 | | 0.3 | |
|  |  | | (1.7) | | (0.3) | | (0.4) | | (0.7) | | (0.1) | |
| ^Ⱡ^Patients were randomly assigned to individualized interval prophylaxis from a Bernoulli distribution with a probability 0.315, based on data from clinical trial 998HB102 (NCT01027364). In the simulations underlying the results of this table, 68.6% of the simulated patients were assigned to receive weekly prophylaxis and 31.4% to individualized interval prophylaxis. | | | | | | | | | | | | |

| **Patients ≥18 \| Target 1%; Control 10%** |  |  |  |  |  |  |  |  |  |  |  |  |
| --- | --- | --- | --- | --- | --- | --- | --- | --- | --- | --- | --- | --- |
|  | **rIX-FP** | | **rFIX** | | **rFIXFc** weekly | | **rFIXFc** ind. Interval | | **rFIXFc^Ⱡ^** weekly and ind.interval | | **N9-GP** | |
| **Steady-state FIX trough levels** Median (IQR) | 7.9 | | 1.0 | | 1.0 | | 1.3 | | 1.1 | | 4.1 | |
|  | (6.2–9.2) | | (1.0–1.0) | | (1.0–1.1) | | (1.2–1.8) | | (1.0–1.3) | | (3.4–5.1) | |
| Mean (SD) | 8.0 | | 1.0 | | 1.1 | | 1.6 | | 1.3 | | 4.3 | |
|  | (2.6) | | (0.1) | | (0.2) | | (0.7) | | (0.5) | | (1.3) | |
| **Patients below target, %** | 0.0% | | 11.3% | | 0.0% | | 0.2% | | 0.1% | | 0.0% | |
| **Dose (IU/kg per week)** Median (IQR) | 35.0 | | 107.0 | | 19.5 | | 53.8 | | 24.5 | | 10.0 | |
|  | (25.0–35.0) | | (63.0–134.2) | | (17.0–25.0) | | (53.8–58.3) | | (17.0–53.8) | | (10.0–10.0) | |
| Mean (SD) | 31 | | 103.7 | | 22.4 | | 58.1 | | 33.6 | | 10 | |
|  | (4.8) | | (46.4) | | (7.5) | | (7.3) | | (18.3) | | (0.0) | |
| **Patients among dose-intervals, %** | 7d | 35.9% | 3d | 24.7% | 7d | 100.0% | 8d | 1.5% | 7d | 68.6% | 7d | 100.0% |
|  | 10d | 17.1% | 3.5d | 17.5% |  |  | 9d | 4.1% | 8d | 0.5% |  |  |
|  | 14d | 38.3% | 4d | 57.7% |  |  | 10d | 7.0% | 9d | 1.3% |  |  |
|  | 21d | 8.7% |  |  |  |  | 11d | 10.9% | 10d | 2.3% |  |  |
|  |  |  |  |  |  |  | 12d | 12.6% | 11d | 3.5% |  |  |
|  |  |  |  |  |  |  | 13d | 63.9% | 12d | 4.1% |  |  |
|  |  |  |  |  |  |  | 14d | 0.1% | 13d | 19.7% |  |  |
|  |  |  |  |  |  |  | 15d | 0.0% | 14d | 0.0% |  |  |
|  |  |  |  |  |  |  | 16d | 0.0% | 15d | 0.0% |  |  |
|  |  |  |  |  |  |  | 17d | 0.0% | 16d | 0.0% |  |  |
|  |  |  |  |  |  |  | 18d | 0.0% | 17d | 0.0% |  |  |
|  |  |  |  |  |  |  | 19d | 0.0% | 18d | 0.0% |  |  |
|  |  |  |  |  |  |  | 20d | 0.0% | 19d | 0.0% |  |  |
|  |  |  |  |  |  |  | 21d | 0.0% | 20d | 0.0% |  |  |
|  |  |  |  |  |  |  |  |  | 21d | 0.0% |  |  |
| **Ratio between weekly dose (IU/kg) FIX product and rIX-FP** Median (IQR) |  | | 3.3 | | 0.7 | | 1.8 | | 0.8 | | 0.3 | |
|  |  | | (2.0–4.6) | | (0.5–0.8) | | (1.5–2.2) | | (0.6–1.5) | | (0.3–0.4) | |
| Mean (SD) |  | | 3.4 | | 0.7 | | 1.9 | | 1.1 | | 0.3 | |
|  |  | | (1.7) | | (0.3) | | (0.4) | | (0.6) | | (0.1) | |
| ^Ⱡ^Patients were randomly assigned to individualized interval prophylaxis from a Bernoulli distribution with a probability 0.315, based on data from clinical trial 998HB102 (NCT01027364). In the simulations underlying the results of this table, 68.6% of the simulated patients were assigned to receive weekly prophylaxis and 31.4% to individualized interval prophylaxis. | | | | | | | | | | | | |

| **Patients ≥18 \| Target 1%; Control 15%** |  |  |  |  |  |  |  |  |  |  |  |  |
| --- | --- | --- | --- | --- | --- | --- | --- | --- | --- | --- | --- | --- |
|  | **rIX-FP** | | **rFIX** | | **rFIXFc** weekly | | **rFIXFc** ind. Interval | | **rFIXFc^Ⱡ^** weekly and ind.interval | | **N9-GP** | |
| **Steady-state FIX trough levels** Median (IQR) | 10.5 | | 1.0 | | 1.0 | | 1.3 | | 1.1 | | 4.1 | |
|  | (8.3–13.0) | | (1.0–1.0) | | (1.0–1.1) | | (1.2–1.8) | | (1.0–1.3) | | (3.4–5.1) | |
| Mean (SD) | 10.5 | | 1.0 | | 1.1 | | 1.6 | | 1.3 | | 4.3 | |
|  | (3.1) | | (0.1) | | (0.2) | | (0.7) | | (0.5) | | (1.3) | |
| **Patients below target, %** | 0.0% | | 11.3% | | 0.0% | | 0.2% | | 0.1% | | 0.0% | |
| **Dose (IU/kg per week)** Median (IQR) | 35.0 | | 107.0 | | 19.5 | | 53.8 | | 24.5 | | 10.0 | |
|  | (35.0–35.0) | | (63.0–134.2) | | (17.0–25.0) | | (53.8–58.3) | | (17.0–53.8) | | (10.0–10.0) | |
| Mean (SD) | 33.4 | | 103.7 | | 22.4 | | 58.1 | | 33.6 | | 10 | |
|  | (3.7) | | (46.4) | | (7.5) | | (7.3) | | (18.3) | | (0.0) | |
| **Patients among dose-intervals, %** | 7d | 71.5% | 3d | 24.7% | 7d | 100.0% | 8d | 1.5% | 7d | 68.6% | 7d | 100.0% |
|  | 10d | 11.3% | 3.5d | 17.5% |  |  | 9d | 4.1% | 8d | 0.5% |  |  |
|  | 14d | 16.2% | 4d | 57.7% |  |  | 10d | 7.0% | 9d | 1.3% |  |  |
|  | 21d | 1.0% |  |  |  |  | 11d | 10.9% | 10d | 2.3% |  |  |
|  |  |  |  |  |  |  | 12d | 12.6% | 11d | 3.5% |  |  |
|  |  |  |  |  |  |  | 13d | 63.9% | 12d | 4.1% |  |  |
|  |  |  |  |  |  |  | 14d | 0.0% | 13d | 19.7% |  |  |
|  |  |  |  |  |  |  | 15d | 0.0% | 14d | 0.0% |  |  |
|  |  |  |  |  |  |  | 16d | 0.0% | 15d | 0.0% |  |  |
|  |  |  |  |  |  |  | 17d | 0.0% | 16d | 0.0% |  |  |
|  |  |  |  |  |  |  | 18d | 0.0% | 17d | 0.0% |  |  |
|  |  |  |  |  |  |  | 19d | 0.0% | 18d | 0.0% |  |  |
|  |  |  |  |  |  |  | 20d | 0.0% | 19d | 0.0% |  |  |
|  |  |  |  |  |  |  | 21d | 0.0% | 20d | 0.0% |  |  |
|  |  |  |  |  |  |  |  |  | 21d | 0.0% |  |  |
| **Ratio between weekly dose (IU/kg) FIX product and rIX-FP** Median (IQR) |  | | 3.2 | | 0.6 | | 1.5 | | 0.7 | | 0.3 | |
|  |  | | (1.9–4.1) | | (0.5–0.8) | | (1.5–2.0) | | (0.5–1.5) | | (0.3–0.3) | |
| Mean (SD) |  | | 3.2 | | 0.7 | | 1.8 | | 1.0 | | 0.3 | |
|  |  | | (1.5) | | (0.2) | | (0.3) | | (0.6) | | (0.0) | |
| ^Ⱡ^Patients were randomly assigned to individualized interval prophylaxis from a Bernoulli distribution with a probability 0.315, based on data from clinical trial 998HB102 (NCT01027364). In the simulations underlying the results of this table, 68.6% of the simulated patients were assigned to receive weekly prophylaxis and 31.4% to individualized interval prophylaxis. | | | | | | | | | | | | |

| **Patients ≥18 \| Target 1%; Control 20%** |  |  |  |  |  |  |  |  |  |  |  |  |
| --- | --- | --- | --- | --- | --- | --- | --- | --- | --- | --- | --- | --- |
|  | **rIX-FP** | | **rFIX** | | **rFIXFc** weekly | | **rFIXFc** ind. Interval | | **rFIXFc^Ⱡ^** weekly and ind.interval | | **N9-GP** | |
| **Steady-state FIX trough levels** Median (IQR) | 11.7 | | 1.0 | | 1.0 | | 1.3 | | 1.1 | | 4.1 | |
|  | (8.6–14.9) | | (1.0–1.0) | | (1.0–1.1) | | (1.2–1.8) | | (1.0–1.3) | | (3.4–5.1) | |
| Mean (SD) | 11.8 | | 1.0 | | 1.1 | | 1.6 | | 1.3 | | 4.3 | |
|  | (4.2) | | (0.1) | | (0.2) | | (0.7) | | (0.5) | | (1.3) | |
| **Patients below target, %** | 0.0% | | 11.3% | | 0.0% | | 0.2% | | 0.1% | | 0.0% | |
| **Dose (IU/kg per week)** Median (IQR) | 35.0 | | 107.0 | | 19.5 | | 53.8 | | 24.5 | | 10.0 | |
|  | (35.0–35.0) | | (63.0–134.2) | | (17.0–25.0) | | (53.8–58.3) | | (17.0–53.8) | | (10.0–10.0) | |
| Mean (SD) | 34.5 | | 103.7 | | 22.4 | | 58.1 | | 33.6 | | 10 | |
|  | (2.2) | | (46.4) | | (7.5) | | (7.3) | | (18.3) | | (0.0) | |
| **Patients among dose-intervals, %** | 7d | 89.9% | 3d | 24.7% | 7d | 100.0% | 8d | 1.5% | 7d | 68.6% | 7d | 100.0% |
|  | 10d | 4.8% | 3.5d | 17.5% |  |  | 9d | 4.1% | 8d | 0.5% |  |  |
|  | 14d | 5.2% | 4d | 57.7% |  |  | 10d | 7.0% | 9d | 1.3% |  |  |
|  | 21d | 0.1% |  |  |  |  | 11d | 10.9% | 10d | 2.3% |  |  |
|  |  |  |  |  |  |  | 12d | 12.6% | 11d | 3.5% |  |  |
|  |  |  |  |  |  |  | 13d | 63.9% | 12d | 4.1% |  |  |
|  |  |  |  |  |  |  | 14d | 0.0% | 13d | 19.7% |  |  |
|  |  |  |  |  |  |  | 15d | 0.0% | 14d | 0.0% |  |  |
|  |  |  |  |  |  |  | 16d | 0.0% | 15d | 0.0% |  |  |
|  |  |  |  |  |  |  | 17d | 0.0% | 16d | 0.0% |  |  |
|  |  |  |  |  |  |  | 18d | 0.0% | 17d | 0.0% |  |  |
|  |  |  |  |  |  |  | 19d | 0.0% | 18d | 0.0% |  |  |
|  |  |  |  |  |  |  | 20d | 0.0% | 19d | 0.0% |  |  |
|  |  |  |  |  |  |  | 21d | 0.0% | 20d | 0.0% |  |  |
|  |  |  |  |  |  |  |  |  | 21d | 0.0% |  |  |
| **Ratio between weekly dose (IU/kg) FIX product and rIX-FP** Median (IQR) |  | | 3.1 | | 0.6 | | 1.5 | | 0.7 | | 0.3 | |
|  |  | | (1.8–3.9) | | (0.5–0.7) | | (1.5–1.8) | | (0.5–1.5) | | (0.3–0.3) | |
| Mean (SD) |  | | 3.0 | | 0.7 | | 1.7 | | 1.0 | | 0.3 | |
|  |  | | (1.4) | | (0.2) | | (0.3) | | (0.5) | | (0.0) | |
| ^Ⱡ^Patients were randomly assigned to individualized interval prophylaxis from a Bernoulli distribution with a probability 0.315, based on data from clinical trial 998HB102 (NCT01027364). In the simulations underlying the results of this table, 68.6% of the simulated patients were assigned to receive weekly prophylaxis and 31.4% to individualized interval prophylaxis. | | | | | | | | | | | | |

| **Patients ≥18 \| Target 2%; Control 5%** | |  | |  | |  | |  | |  | |  | |  | |  | |  | |  | |  | |  | |
| --- | --- | --- | --- | --- | --- | --- | --- | --- | --- | --- | --- | --- | --- | --- | --- | --- | --- | --- | --- | --- | --- | --- | --- | --- | --- |
|  | | **rIX-FP** | | | | **rFIX** | | | | **rFIXFc** weekly | | | | **rFIXFc** ind. Interval | | | | **rFIXFc^Ⱡ^** weekly and ind.interval | | | | **N9-GP** | | | |
| **Steady-state FIX trough levels** Median (IQR) | | 4.8 | | | | 2.0 | | | | 2.0 | | | | 2.3 | | | | 2.0 | | | | 4.1 | | | |
|  |  | (3.6–7.2) | | | | (1.6–2.0) | | | | (2.0–2.0) | | | | (2.1–2.4) | | | | (2.0–2.1) | | | | (3.4–5.1) | | | |
| Mean (SD) | | 5.9 | | | | 1.7 | | | | 2.0 | | | | 2.3 | | | | 2.1 | | | | 4.3 | | | |
|  |  | (3.4) | | | | (0.5) | | | | (0.1) | | | | (0.3) | | | | (0.2) | | | | (1.3) | | | |
| **Patients below target, %** | | 0.0% | | | | 33.2% | | | | 1.8% | | | | 5.9% | | | | 3.1% | | | | 0.0% | | | |
| **Dose (IU/kg per week)** Median (IQR) | | 33.3 | | | | 143.0 | | | | 38.5 | | | | 70.0 | | | | 48.0 | | | | 10.0 | | | |
|  |  | (25.0–33.3) | | | | (116.0–182.0) | | | | (30.5–50.0) | | | | (58.3–77.8) | | | | (34.0–63.6) | | | | (10.0–10.0) | | | |
| Mean (SD) | | 29.9 | | | | 140.6 | | | | 41.7 | | | | 68.5 | | | | 50.1 | | | | 10 | | | |
|  |  | (4.3) | | | | (39.8) | | | | (15.7) | | | | (11.9) | | | | (19.3) | | | | (0.4) | | | |
| **Patients among dose-intervals, %** | | 7d | | 4.3% | | 3d | | 54.6% | | 7d | | 100.0% | | 8d | | 17.1% | | 7d | | 68.6% | | 7d | | 100.0% | |
|  |  | 10d | | 7.3% | | 3.5d | | 17.4% | |  | |  | | 9d | | 16.8% | | 8d | | 5.7% | |  | |  | |
|  |  | 14d | | 43.4% | | 4d | | 27.9% | |  | |  | | 10d | | 18.4% | | 9d | | 5.3% | |  | |  | |
|  |  | 21d | | 45.1% | |  | |  | |  | |  | | 11d | | 16.1% | | 10d | | 5.7% | |  | |  | |
|  |  |  | |  | |  | |  | |  | |  | | 12d | | 11.2% | | 11d | | 5.1% | |  | |  | |
|  |  |  | |  | |  | |  | |  | |  | | 13d | | 15.5% | | 12d | | 3.4% | |  | |  | |
|  |  |  | |  | |  | |  | |  | |  | | 14d | | 4.8% | | 13d | | 4.7% | |  | |  | |
|  |  |  | |  | |  | |  | |  | |  | | 15d | | 0.0% | | 14d | | 1.5% | |  | |  | |
|  |  |  | |  | |  | |  | |  | |  | | 16d | | 0.0% | | 15d | | 0.0% | |  | |  | |
|  |  |  | |  | |  | |  | |  | |  | | 17d | | 0.0% | | 16d | | 0.0% | |  | |  | |
|  |  |  | |  | |  | |  | |  | |  | | 18d | | 0.0% | | 17d | | 0.0% | |  | |  | |
|  |  |  | |  | |  | |  | |  | |  | | 19d | | 0.0% | | 18d | | 0.0% | |  | |  | |
|  |  |  | |  | |  | |  | |  | |  | | 20d | | 0.0% | | 19d | | 0.0% | |  | |  | |
|  |  |  | |  | |  | |  | |  | |  | | 21d | | 0.0% | | 20d | | 0.0% | |  | |  | |
|  |  |  | |  | |  | |  | |  | |  | |  | |  | | 21d | | 0.0% | |  | |  | |
| **Ratio between weekly dose (IU/kg) FIX product and rIX-FP** Median (IQR) | |  | | | | 5.0 | | | | 1.3 | | | | 2.3 | | | | 1.6 | | | | 0.3 | | | |
|  |  |  | | | | (3.7–5.5) | | | | (1.0–1.7) | | | | (1.9–2.6) | | | | (1.1–2.2) | | | | (0.3–0.4) | | | |
| Mean (SD) | |  | | | | 4.8 | | | | 1.4 | | | | 2.3 | | | | 1.7 | | | | 0.3 | | | |
|  |  |  | | | | (1.6) | | | | (0.6) | | | | (0.5) | | | | (0.7) | | | | (0.1) | | | |
| ^Ⱡ^Patients were randomly assigned to individualized interval prophylaxis from a Bernoulli distribution with a probability 0.315, based on data from clinical trial 998HB102 (NCT01027364). In the simulations underlying the results of this table, 68.6% of the simulated patients were assigned to receive weekly prophylaxis and 31.4% to individualized interval prophylaxis. | | | | | | | | | | | | | | | | | | | | | | | | | |
| **Patients ≥18 \| Target 2%; Control 7.5%** |  | |  | |  | |  | |  | |  | |  | |  | |  | |  | |  | |  | |  |
|  | **rIX-FP** | | | | **rFIX** | | | | **rFIXFc** weekly | | | | **rFIXFc** ind. Interval | | | | **rFIXFc^Ⱡ^** weekly and ind.interval | | | | **N9-GP** | | | |  |
| **Steady-state FIX trough levels** Median (IQR) | 6.2 | | | | 2.0 | | | | 2.0 | | | | 2.3 | | | | 2.0 | | | | 4.1 | | | |  |
|  | (4.8–7.3) | | | | (1.6–2.0) | | | | (2.0–2.0) | | | | (2.1–2.4) | | | | (2.0–2.1) | | | | (3.4–5.1) | | | |  |
| Mean (SD) | 6.8 | | | | 1.7 | | | | 2.0 | | | | 2.3 | | | | 2.1 | | | | 4.3 | | | |  |
|  | (2.9) | | | | (0.5) | | | | (0.1) | | | | (0.4) | | | | (0.3) | | | | (1.3) | | | |  |
| **Patients below target, %** | 0.0% | | | | 33.2% | | | | 1.8% | | | | 5.9% | | | | 3.1% | | | | 0.0% | | | |  |
| **Dose (IU/kg per week)** Median (IQR) | 33.3 | | | | 143.0 | | | | 38.5 | | | | 70.0 | | | | 48.0 | | | | 10.0 | | | |  |
|  | (25.0–35.0) | | | | (116.0–182.0) | | | | (30.5–50.0) | | | | (58.3–77.8) | | | | (34–63.6) | | | | (10.0–10.0) | | | |  |
| Mean (SD) | 29.9 | | | | 140.6 | | | | 41.7 | | | | 68.7 | | | | 50.2 | | | | 10 | | | |  |
|  | (4.7) | | | | (39.8) | | | | (15.7) | | | | (11.7) | | | | (19.3) | | | | (0.4) | | | |  |
| **Patients among dose-intervals, %** | 7d | | 16.9% | | 3d | | 54.6% | | 7d | | 100.0% | | 8d | | 17.1% | | 7d | | 68.6% | | 7d | | 100.0% | |  |
|  | 10d | | 14.8% | | 3.5d | | 17.4% | |  | |  | | 9d | | 16.8% | | 8d | | 5.7% | |  | |  | |  |
|  | 14d | | 47.9% | | 4d | | 27.9% | |  | |  | | 10d | | 18.4% | | 9d | | 5.3% | |  | |  | |  |
|  | 21d | | 20.4% | |  | |  | |  | |  | | 11d | | 16.1% | | 10d | | 5.7% | |  | |  | |  |
|  |  | |  | |  | |  | |  | |  | | 12d | | 11.2% | | 11d | | 5.1% | |  | |  | |  |
|  |  | |  | |  | |  | |  | |  | | 13d | | 20.1% | | 12d | | 3.4% | |  | |  | |  |
|  |  | |  | |  | |  | |  | |  | | 14d | | 0.3% | | 13d | | 6.1% | |  | |  | |  |
|  |  | |  | |  | |  | |  | |  | | 15d | | 0.0% | | 14d | | 0.1% | |  | |  | |  |
|  |  | |  | |  | |  | |  | |  | | 16d | | 0.0% | | 15d | | 0.0% | |  | |  | |  |
|  |  | |  | |  | |  | |  | |  | | 17d | | 0.0% | | 16d | | 0.0% | |  | |  | |  |
|  |  | |  | |  | |  | |  | |  | | 18d | | 0.0% | | 17d | | 0.0% | |  | |  | |  |
|  |  | |  | |  | |  | |  | |  | | 19d | | 0.0% | | 18d | | 0.0% | |  | |  | |  |
|  |  | |  | |  | |  | |  | |  | | 20d | | 0.0% | | 19d | | 0.0% | |  | |  | |  |
|  |  | |  | |  | |  | |  | |  | | 21d | | 0.0% | | 20d | | 0.0% | |  | |  | |  |
|  |  | |  | |  | |  | |  | |  | |  | |  | | 21d | | 0.0% | |  | |  | |  |
| **Ratio between weekly dose (IU/kg) FIX product and rIX-FP** Median (IQR) |  | | | | 5.0 | | | | 1.3 | | | | 2.2 | | | | 1.6 | | | | 0.3 | | | |  |
|  |  | | | | (3.7–5.6) | | | | (1.0–1.7) | | | | (2.0–2.8) | | | | (1.1–2.2) | | | | (0.3–0.4) | | | |  |
| Mean (SD) |  | | | | 4.8 | | | | 1.4 | | | | 2.4 | | | | 1.7 | | | | 0.3 | | | |  |
|  |  | | | | (1.6) | | | | (0.6) | | | | (0.6) | | | | (0.7) | | | | (0.1) | | | |  |
| ^Ⱡ^Patients were randomly assigned to individualized interval prophylaxis from a Bernoulli distribution with a probability 0.315, based on data from clinical trial 998HB102 (NCT01027364). In the simulations underlying the results of this table, 68.6% of the simulated patients were assigned to receive weekly prophylaxis and 31.4% to individualized interval prophylaxis. | | | | | | | | | | | | | | | | | | | | | | | | |  |

| **Patients ≥18 \| Target 2%; Control 10%** |  |  |  |  |  |  |  |  |  |  |  |  |
| --- | --- | --- | --- | --- | --- | --- | --- | --- | --- | --- | --- | --- |
|  | **rIX-FP** | | **rFIX** | | **rFIXFc** weekly | | **rFIXFc** ind. Interval | | **rFIXFc^Ⱡ^** weekly and ind.interval | | **N9-GP** | |
| **Steady-state FIX trough levels** Median (IQR) | 7.9 | | 2.0 | | 2.0 | | 2.3 | | 2.0 | | 4.1 | |
|  | (6.2–9.2) | | (1.6–2.0) | | (2.0–2.0) | | (2.1–2.4) | | (2.0–2.1) | | (3.4–5.1) | |
| Mean (SD) | 8.0 | | 1.7 | | 2.0 | | 2.3 | | 2.1 | | 4.3 | |
|  | (2.6) | | (0.5) | | (0.1) | | (0.4) | | (0.3) | | (1.3) | |
| **Patients below target, %** | 0.0% | | 33.2% | | 1.8% | | 5.9% | | 3.1% | | 0.0% | |
| **Dose (IU/kg per week)** Median (IQR) | 35.0 | | 143.0 | | 38.5 | | 70.0 | | 48.0 | | 10.0 | |
|  | (25.0–35.0) | | (116.0–182.0) | | (30.5–50.0) | | (58.3–77.8) | | (34.0–63.6) | | (10.0–10.0) | |
| Mean (SD) | 31 | | 140.6 | | 41.7 | | 68.7 | | 50.2 | | 10 | |
|  | (4.8) | | (39.8) | | (15.7) | | (11.7) | | (19.3) | | (0.4) | |
| **Patients among dose-intervals, %** | 7d | 35.9% | 3d | 54.6% | 7d | 100.0% | 8d | 17.1% | 7d | 68.6% | 7d | 100.0% |
|  | 10d | 17.1% | 3.5d | 17.4% |  |  | 9d | 16.8% | 8d | 5.7% |  |  |
|  | 14d | 38.3% | 4d | 27.9% |  |  | 10d | 18.4% | 9d | 5.3% |  |  |
|  | 21d | 8.7% |  |  |  |  | 11d | 16.1% | 10d | 5.7% |  |  |
|  |  |  |  |  |  |  | 12d | 11.2% | 11d | 5.1% |  |  |
|  |  |  |  |  |  |  | 13d | 20.4% | 12d | 3.4% |  |  |
|  |  |  |  |  |  |  | 14d | 0.1% | 13d | 6.2% |  |  |
|  |  |  |  |  |  |  | 15d | 0.0% | 14d | 0.0% |  |  |
|  |  |  |  |  |  |  | 16d | 0.0% | 15d | 0.0% |  |  |
|  |  |  |  |  |  |  | 17d | 0.0% | 16d | 0.0% |  |  |
|  |  |  |  |  |  |  | 18d | 0.0% | 17d | 0.0% |  |  |
|  |  |  |  |  |  |  | 19d | 0.0% | 18d | 0.0% |  |  |
|  |  |  |  |  |  |  | 20d | 0.0% | 19d | 0.0% |  |  |
|  |  |  |  |  |  |  | 21d | 0.0% | 20d | 0.0% |  |  |
|  |  |  |  |  |  |  |  |  | 21d | 0.0% |  |  |
| **Ratio between weekly dose (IU/kg) FIX product and rIX-FP** Median (IQR) |  | | 4.8 | | 1.3 | | 2.2 | | 1.5 | | 0.3 | |
|  |  | | (3.6–5.3) | | (1.0–1.7) | | (1.8–2.5) | | (1.1–2.2) | | (0.3–0.4) | |
| Mean (SD) |  | | 4.6 | | 1.4 | | 2.3 | | 1.7 | | 0.3 | |
|  |  | | (1.5) | | (0.6) | | (0.5) | | (0.7) | | (0.1) | |
| ^Ⱡ^Patients were randomly assigned to individualized interval prophylaxis from a Bernoulli distribution with a probability 0.315, based on data from clinical trial 998HB102 (NCT01027364). In the simulations underlying the results of this table, 68.6% of the simulated patients were assigned to receive weekly prophylaxis and 31.4% to individualized interval prophylaxis. | | | | | | | | | | | | |

| **Patients ≥18 \| Target 2%; Control 15%** |  |  |  |  |  |  |  |  |  |  |  |  |
| --- | --- | --- | --- | --- | --- | --- | --- | --- | --- | --- | --- | --- |
|  | **rIX-FP** | | **rFIX** | | **rFIXFc** weekly | | **rFIXFc** ind. Interval | | **rFIXFc^Ⱡ^** weekly and ind.interval | | **N9-GP** | |
| **Steady-state FIX trough levels** Median (IQR) | 10.5 | | 2.0 | | 2.0 | | 2.3 | | 2.0 | | 4.1 | |
|  | (8.3–13.0) | | (1.6–2.0) | | (2.0–2.0) | | (2.1–2.4) | | (2.0–2.1) | | (3.4–5.1) | |
| Mean (SD) | 10.5 | | 1.7 | | 2.0 | | 2.3 | | 2.1 | | 4.3 | |
|  | (3.1) | | (0.5) | | (0.1) | | (0.4) | | (0.3) | | (1.3) | |
| **Patients below target, %** | 0.0% | | 33.2% | | 1.8% | | 5.9% | | 3.1% | | 0.0% | |
| **Dose (IU/kg per week)** Median (IQR) | 35.0 | | 143.0 | | 38.5 | | 70.0 | | 48.0 | | 10.0 | |
|  | (35.0–35.0) | | (116.0–182.0) | | (30.5–50.0) | | (58.3–77.8) | | (34.0–63.6) | | (10.0–10.0) | |
| Mean (SD) | 33.4 | | 140.6 | | 41.7 | | 68.7 | | 50.2 | | 10 | |
|  | (3.7) | | (39.8) | | (15.7) | | (11.7) | | (19.3) | | (0.4) | |
| **Patients among dose-intervals, %** | 7d | 71.5% | 3d | 54.6% | 7d | 100.0% | 8d | 17.1% | 7d | 68.6% | 7d | 100.0% |
|  | 10d | 11.3% | 3.5d | 17.4% |  |  | 9d | 16.8% | 8d | 5.7% |  |  |
|  | 14d | 16.2% | 4d | 27.9% |  |  | 10d | 18.4% | 9d | 5.3% |  |  |
|  | 21d | 1.0% |  |  |  |  | 11d | 16.1% | 10d | 5.7% |  |  |
|  |  |  |  |  |  |  | 12d | 11.2% | 11d | 5.1% |  |  |
|  |  |  |  |  |  |  | 13d | 20.4% | 12d | 3.4% |  |  |
|  |  |  |  |  |  |  | 14d | 0.0% | 13d | 6.2% |  |  |
|  |  |  |  |  |  |  | 15d | 0.0% | 14d | 0.0% |  |  |
|  |  |  |  |  |  |  | 16d | 0.0% | 15d | 0.0% |  |  |
|  |  |  |  |  |  |  | 17d | 0.0% | 16d | 0.0% |  |  |
|  |  |  |  |  |  |  | 18d | 0.0% | 17d | 0.0% |  |  |
|  |  |  |  |  |  |  | 19d | 0.0% | 18d | 0.0% |  |  |
|  |  |  |  |  |  |  | 20d | 0.0% | 19d | 0.0% |  |  |
|  |  |  |  |  |  |  | 21d | 0.0% | 20d | 0.0% |  |  |
|  |  |  |  |  |  |  |  |  | 21d | 0.0% |  |  |
| **Ratio between weekly dose (IU/kg) FIX product and rIX-FP** Median (IQR) |  | | 4.3 | | 1.2 | | 2.0 | | 1.5 | | 0.3 | |
|  |  | | (3.4–5.2) | | (0.9–1.5) | | (1.7–2.5) | | (1.0–2.0) | | (0.3–0.3) | |
| Mean (SD) |  | | 4.3 | | 1.3 | | 2.1 | | 1.5 | | 0.3 | |
|  |  | | (1.3) | | (0.5) | | (0.5) | | (0.6) | | (0.0) | |
| ^Ⱡ^Patients were randomly assigned to individualized interval prophylaxis from a Bernoulli distribution with a probability 0.315, based on data from clinical trial 998HB102 (NCT01027364). In the simulations underlying the results of this table, 68.6% of the simulated patients were assigned to receive weekly prophylaxis and 31.4% to individualized interval prophylaxis. | | | | | | | | | | | | |

| **Patients ≥18 \| Target 2%; Control 20%** |  |  |  |  |  |  |  |  |  |  |  |  |
| --- | --- | --- | --- | --- | --- | --- | --- | --- | --- | --- | --- | --- |
|  | **rIX-FP** | | **rFIX** | | **rFIXFc** weekly | | **rFIXFc** ind. Interval | | **rFIXFc^Ⱡ^** weekly and ind.interval | | **N9-GP** | |
| **Steady-state FIX trough levels** Median (IQR) | 11.7 | | 2.0 | | 2.0 | | 2.3 | | 2.0 | | 4.1 | |
|  | (8.6–14.9) | | (1.6–2.0) | | (2.0–2.0) | | (2.1–2.4) | | (2.0–2.1) | | (3.4–5.1) | |
| Mean (SD) | 11.8 | | 1.7 | | 2.0 | | 2.3 | | 2.1 | | 4.3 | |
|  | (4.2) | | (0.5) | | (0.1) | | (0.4) | | (0.3) | | (1.3) | |
| **Patients below target, %** | 0.0% | | 33.2% | | 1.8% | | 5.9% | | 3.1% | | 0.0% | |
| **Dose (IU/kg per week)** Median (IQR) | 35.0 | | 143.0 | | 38.5 | | 70.0 | | 48.0 | | 10.0 | |
|  | (35.0–35.0) | | (116.0–182.0) | | (30.5–50.0) | | (58.3–77.8) | | (34.0–63.6) | | (10.0–10.0) | |
| Mean (SD) | 34.5 | | 140.6 | | 41.7 | | 68.7 | | 50.2 | | 10 | |
|  | (2.2) | | (39.8) | | (15.7) | | (11.7) | | (19.3) | | (0.4) | |
| **Patients among dose-intervals, %** | 7d | 89.9% | 3d | 54.6% | 7d | 100.0% | 8d | 17.1% | 7d | 68.6% | 7d | 100.0% |
|  | 10d | 4.8% | 3.5d | 17.4% |  |  | 9d | 16.8% | 8d | 5.7% |  |  |
|  | 14d | 5.2% | 4d | 27.9% |  |  | 10d | 18.4% | 9d | 5.3% |  |  |
|  | 21d | 0.1% |  |  |  |  | 11d | 16.1% | 10d | 5.7% |  |  |
|  |  |  |  |  |  |  | 12d | 11.2% | 11d | 5.1% |  |  |
|  |  |  |  |  |  |  | 13d | 20.4% | 12d | 3.4% |  |  |
|  |  |  |  |  |  |  | 14d | 0.0% | 13d | 6.2% |  |  |
|  |  |  |  |  |  |  | 15d | 0.0% | 14d | 0.0% |  |  |
|  |  |  |  |  |  |  | 16d | 0.0% | 15d | 0.0% |  |  |
|  |  |  |  |  |  |  | 17d | 0.0% | 16d | 0.0% |  |  |
|  |  |  |  |  |  |  | 18d | 0.0% | 17d | 0.0% |  |  |
|  |  |  |  |  |  |  | 19d | 0.0% | 18d | 0.0% |  |  |
|  |  |  |  |  |  |  | 20d | 0.0% | 19d | 0.0% |  |  |
|  |  |  |  |  |  |  | 21d | 0.0% | 20d | 0.0% |  |  |
|  |  |  |  |  |  |  |  |  | 21d | 0.0% |  |  |
| **Ratio between weekly dose (IU/kg) FIX product and rIX-FP** Median (IQR) |  | | 4.2 | | 1.1 | | 2.0 | | 1.4 | | 0.3 | |
|  |  | | (3.3–5.2) | | (0.9–1.5) | | (1.7–2.2) | | (1.0–1.8) | | (0.3–0.3) | |
| Mean (SD) |  | | 4.1 | | 1.2 | | 2.0 | | 1.5 | | 0.3 | |
|  |  | | (1.2) | | (0.5) | | (0.4) | | (0.6) | | (0.0) | |
| ^Ⱡ^Patients were randomly assigned to individualized interval prophylaxis from a Bernoulli distribution with a probability 0.315, based on data from clinical trial 998HB102 (NCT01027364). In the simulations underlying the results of this table, 68.6% of the simulated patients were assigned to receive weekly prophylaxis and 31.4% to individualized interval prophylaxis. | | | | | | | | | | | | |

| **Patients ≥18 \| Target 3%; Control 5%** |  |  |  |  |  |  |  |  |  |  |  |  |
| --- | --- | --- | --- | --- | --- | --- | --- | --- | --- | --- | --- | --- |
|  | **rIX-FP** | | **rFIX** | | **rFIXFc** weekly | | **rFIXFc** ind. Interval | | **rFIXFc^Ⱡ^** weekly and ind.interval | | **N9-GP** | |
| **Steady-state FIX trough levels** Median (IQR) | 4.8 | | 2.8 | | 3.0 | | 3.2 | | 3.0 | | 4.1 | |
|  | (3.6–7.2) | | (1.6–3.0) | | (3.0–3.0) | | (3.0–3.5) | | (3.0–3.0) | | (3.4–5.1) | |
| Mean (SD) | 6.0 | | 2.3 | | 2.9 | | 3.2 | | 3.0 | | 4.4 | |
|  | (3.3) | | (0.9) | | (0.2) | | (0.7) | | (0.4) | | (1.2) | |
| **Patients below target, %** | 0.0% | | 54.2% | | 13.9% | | 24.6% | | 17.4% | | 0.0% | |
| **Dose (IU/kg per week)** Median (IQR) | 33.3 | | 182.0 | | 58.0 | | 77.8 | | 67.0 | | 10.0 | |
|  | (25.0–33.3) | | (137.0–182.0) | | (45.5–74.5) | | (70.0–87.5) | | (50.5–86.0) | | (10.0–10.0) | |
| Mean (SD) | 30.4 | | 159.3 | | 59.8 | | 78.2 | | 65.5 | | 10.4 | |
|  | (4.1) | | (31.2) | | (18.1) | | (10.5) | | (18.3) | | (1.4) | |
| **Patients among dose-intervals, %** | 7d | 4.3% | 3d | 73.6% | 7d | 100.0% | 8d | 46.7% | 7d | 68.6% | 7d | 100.0% |
|  | 10d | 7.3% | 3.5d | 12.9% |  |  | 9d | 20.8% | 8d | 14.8% |  |  |
|  | 14d | 43.4% | 4d | 13.6% |  |  | 10d | 16.4% | 9d | 6.7% |  |  |
|  | 21d | 45.1% |  |  |  |  | 11d | 8.7% | 10d | 5.0% |  |  |
|  |  |  |  |  |  |  | 12d | 4.4% | 11d | 2.6% |  |  |
|  |  |  |  |  |  |  | 13d | 0.7% | 12d | 1.3% |  |  |
|  |  |  |  |  |  |  | 14d | 2.2% | 13d | 0.2% |  |  |
|  |  |  |  |  |  |  | 15d | 0.0% | 14d | 0.8% |  |  |
|  |  |  |  |  |  |  | 16d | 0.0% | 15d | 0.0% |  |  |
|  |  |  |  |  |  |  | 17d | 0.0% | 16d | 0.0% |  |  |
|  |  |  |  |  |  |  | 18d | 0.0% | 17d | 0.0% |  |  |
|  |  |  |  |  |  |  | 19d | 0.0% | 18d | 0.0% |  |  |
|  |  |  |  |  |  |  | 20d | 0.0% | 19d | 0.0% |  |  |
|  |  |  |  |  |  |  | 21d | 0.0% | 20d | 0.0% |  |  |
|  |  |  |  |  |  |  |  |  | 21d | 0.0% |  |  |
| **Ratio between weekly dose (IU/kg) FIX product and rIX-FP** Median (IQR) |  | | 5.5 | | 1.9 | | 2.6 | | 2.2 | | 0.3 | |
|  |  | | (4.5–6.2) | | (1.5–2.5) | | (2.3–3.0) | | (1.7–2.6) | | (0.3–0.4) | |
| Mean (SD) |  | | 5.3 | | 2.0 | | 2.6 | | 2.2 | | 0.3 | |
|  |  | | (1.3) | | (0.7) | | (0.5) | | (0.7) | | (0.1) | |
| ^Ⱡ^Patients were randomly assigned to individualized interval prophylaxis from a Bernoulli distribution with a probability 0.315, based on data from clinical trial 998HB102 (NCT01027364). In the simulations underlying the results of this table, 68.6% of the simulated patients were assigned to receive weekly prophylaxis and 31.4% to individualized interval prophylaxis. | | | | | | | | | | | | |

| **Patients ≥18 \| Target 3%; Control 7.5%** |  |  |  |  |  |  |  |  |  |  |  |  |
| --- | --- | --- | --- | --- | --- | --- | --- | --- | --- | --- | --- | --- |
|  | **rIX-FP** | | **rFIX** | | **rFIXFc** weekly | | **rFIXFc** ind. int. | | **rFIXFc^Ⱡ^** weekly and ind.interval | | **N9-GP** | |
| **Steady-state FIX trough levels** Median (IQR) | 6.2 | | 2.8 | | 3.0 | | 3.2 | | 3.0 | | 4.1 | |
|  | (4.8–7.3) | | (1.6–3.0) | | (3.0–3.0) | | (3.0–3.5) | | (3.0–3.0) | | (3.4–5.1) | |
| Mean (SD) | 6.8 | | 2.3 | | 2.9 | | 3.1 | | 3.0 | | 4.4 | |
|  | (2.9) | | (0.8) | | (0.2) | | (0.6) | | (0.4) | | (1.2) | |
| **Patients below target, %** | 0.0% | | 54.2% | | 13.9% | | 24.6% | | 17.4% | | 0.0% | |
| **Dose (IU/kg per week)** Median (IQR) | 33.3 | | 182.0 | | 58.0 | | 77.8 | | 66.0 | | 10.0 | |
|  | (25.0–35.0) | | (137.0–182.0) | | (45.5–74.5) | | (70.0–87.5) | | (51.0–86.0) | | (10.0–10.0) | |
| Mean (SD) | 29.9 | | 159.3 | | 59.8 | | 77.8 | | 65.4 | | 10.4 | |
|  | (4.7) | | (31.2) | | (18.1) | | (10.9) | | (18.3) | | (1.4) | |
| **Patients among dose-intervals, %** | 7d | 16.9% | 3d | 73.6% | 7d | 100.0% | 8d | 46.7% | 7d | 68.6% | 7d | 100.0% |
|  | 10d | 14.8% | 3.5d | 12.9% |  |  | 9d | 20.8% | 8d | 14.8% |  |  |
|  | 14d | 47.9% | 4d | 13.6% |  |  | 10d | 13.8% | 9d | 6.7% |  |  |
|  | 21d | 20.4% |  |  |  |  | 11d | 8.7% | 10d | 4.3% |  |  |
|  |  |  |  |  |  |  | 12d | 5.0% | 11d | 2.6% |  |  |
|  |  |  |  |  |  |  | 13d | 4.6% | 12d | 1.5% |  |  |
|  |  |  |  |  |  |  | 14d | 0.3% | 13d | 1.4% |  |  |
|  |  |  |  |  |  |  | 15d | 0.0% | 14d | 0.1% |  |  |
|  |  |  |  |  |  |  | 16d | 0.0% | 15d | 0.0% |  |  |
|  |  |  |  |  |  |  | 17d | 0.0% | 16d | 0.0% |  |  |
|  |  |  |  |  |  |  | 18d | 0.0% | 17d | 0.0% |  |  |
|  |  |  |  |  |  |  | 19d | 0.0% | 18d | 0.0% |  |  |
|  |  |  |  |  |  |  | 20d | 0.0% | 19d | 0.0% |  |  |
|  |  |  |  |  |  |  | 21d | 0.0% | 20d | 0.0% |  |  |
|  |  |  |  |  |  |  |  |  | 21d | 0.0% |  |  |
| **Ratio between weekly dose (IU/kg) FIX product and rIX-FP** Median (IQR) |  | | 5.2 | | 2.0 | | 2.5 | | 2.2 | | 0.4 | |
|  |  | | (4.7–7.3) | | (1.5–2.5) | | (2.2–3.1) | | (1.7–2.6) | | (0.3–0.4) | |
| Mean (SD) |  | | 5.5 | | 2.1 | | 2.7 | | 2.2 | | 0.4 | |
|  |  | | (1.4) | | (0.7) | | (0.6) | | (0.7) | | (0.1) | |
| ^Ⱡ^Patients were randomly assigned to individualized interval prophylaxis from a Bernoulli distribution with a probability 0.315, based on data from clinical trial 998HB102 (NCT01027364). In the simulations underlying the results of this table, 68.6% of the simulated patients were assigned to receive weekly prophylaxis and 31.4% to individualized interval prophylaxis. | | | | | | | | | | | | |

| **Patients ≥18 \| Target 3%; Control 10%** |  |  |  |  |  |  |  |  |  |  |  |  |
| --- | --- | --- | --- | --- | --- | --- | --- | --- | --- | --- | --- | --- |
|  | **rIX-FP** | | **rFIX** | | **rFIXFc** weekly | | **rFIXFc** ind. Interval | | **rFIXFc^Ⱡ^** weekly and ind.interval | | **N9-GP** | |
| **Steady-state FIX trough levels** Median (IQR) | 7.9 | | 2.8 | | 3.0 | | 3.2 | | 3.0 | | 4.1 | |
|  | (6.2–9.2) | | (1.6–3.0) | | (3.0–3.0) | | (3.0–3.5) | | (3.0–3.0) | | (3.4–5.1) | |
| Mean (SD) | 8.0 | | 2.3 | | 2.9 | | 3.1 | | 3.0 | | 4.4 | |
|  | (2.6) | | (0.8) | | (0.2) | | (0.6) | | (0.4) | | (1.2) | |
| **Patients below target, %** | 0.0% | | 54.2% | | 13.9% | | 24.6% | | 17.4% | | 0.0% | |
| **Dose (IU/kg per week)** Median (IQR) | 35.0 | | 182.0 | | 58.0 | | 77.8 | | 66.0 | | 10.0 | |
|  | (25.0–35.0) | | (137.0–182.0) | | (45.5–74.5) | | (70.0–87.5) | | (51.0–86.0) | | (10.0–10.0) | |
| Mean (SD) | 31 | | 159.3 | | 59.8 | | 77.9 | | 65.4 | | 10.4 | |
|  | (4.8) | | (31.2) | | (18.1) | | (10.8) | | (18.3) | | (1.4) | |
| **Patients among dose-intervals, %** | 7d | 35.9% | 3d | 73.6% | 7d | 100.0% | 8d | 46.7% | 7d | 68.6% | 7d | 100.0% |
|  | 10d | 17.1% | 3.5d | 12.9% |  |  | 9d | 20.8% | 8d | 14.8% |  |  |
|  | 14d | 38.3% | 4d | 13.6% |  |  | 10d | 13.8% | 9d | 6.7% |  |  |
|  | 21d | 8.7% |  |  |  |  | 11d | 8.7% | 10d | 4.3% |  |  |
|  |  |  |  |  |  |  | 12d | 5.0% | 11d | 2.6% |  |  |
|  |  |  |  |  |  |  | 13d | 4.8% | 12d | 1.5% |  |  |
|  |  |  |  |  |  |  | 14d | 0.1% | 13d | 1.5% |  |  |
|  |  |  |  |  |  |  | 15d | 0.0% | 14d | 0.0% |  |  |
|  |  |  |  |  |  |  | 16d | 0.0% | 15d | 0.0% |  |  |
|  |  |  |  |  |  |  | 17d | 0.0% | 16d | 0.0% |  |  |
|  |  |  |  |  |  |  | 18d | 0.0% | 17d | 0.0% |  |  |
|  |  |  |  |  |  |  | 19d | 0.0% | 18d | 0.0% |  |  |
|  |  |  |  |  |  |  | 20d | 0.0% | 19d | 0.0% |  |  |
|  |  |  |  |  |  |  | 21d | 0.0% | 20d | 0.0% |  |  |
|  |  |  |  |  |  |  |  |  | 21d | 0.0% |  |  |
| **Ratio between weekly dose (IU/kg) FIX product and rIX-FP** Median (IQR) |  | | 5.2 | | 1.9 | | 2.5 | | 2.2 | | 0.3 | |
|  |  | | (4.4–6.0) | | (1.4–2.5) | | (2.2–3.1) | | (1.6–2.5) | | (0.3–0.4) | |
| Mean (SD) |  | | 5.3 | | 2.0 | | 2.6 | | 2.2 | | 0.3 | |
|  |  | | (1.4) | | (0.7) | | (0.6) | | (0.7) | | (0.1) | |
| ^Ⱡ^Patients were randomly assigned to individualized interval prophylaxis from a Bernoulli distribution with a probability 0.315, based on data from clinical trial 998HB102 (NCT01027364). In the simulations underlying the results of this table, 68.6% of the simulated patients were assigned to receive weekly prophylaxis and 31.4% to individualized interval prophylaxis. | | | | | | | | | | | | |

| **Patients ≥18 \| Target 3%; Control 15%** |  |  |  |  |  |  |  |  |  |  |  |  |
| --- | --- | --- | --- | --- | --- | --- | --- | --- | --- | --- | --- | --- |
|  | **rIX-FP** | | **rFIX** | | **rFIXFc** weekly | | **rFIXFc** ind. Interval | | **rFIXFc^Ⱡ^** weekly and ind.interval | | **N9-GP** | |
| **Steady-state FIX trough levels** Median (IQR) | 10.5 | | 2.8 | | 3.0 | | 3.2 | | 3.0 | | 4.1 | |
|  | (8.3–13.0) | | (1.6–3.0) | | (3.0–3.0) | | (3.0–3.5) | | (3.0–3.0) | | (3.4–5.1) | |
| Mean (SD) | 10.5 | | 2.3 | | 2.9 | | 3.1 | | 3.0 | | 4.4 | |
|  | (3.1) | | (0.8) | | (0.2) | | (0.6) | | (0.4) | | (1.2) | |
| **Patients below target, %** | 0.0% | | 54.2% | | 13.9% | | 24.6% | | 17.4% | | 0.0% | |
| **Dose (IU/kg per week)** Median (IQR) | 35.0 | | 182.0 | | 58.0 | | 77.8 | | 66.0 | | 10.0 | |
|  | (35.0–35.0) | | (137.0–182.0) | | (45.5–74.5) | | (70.0–87.5) | | (51.0–86.0) | | (10.0–10.0) | |
| Mean (SD) | 33.4 | | 159.3 | | 59.8 | | 77.9 | | 65.4 | | 10.4 | |
|  | (3.7) | | (31.2) | | (18.1) | | (10.8) | | (18.3) | | (1.4) | |
| **Patients among dose-intervals, %** | 7d | 71.5% | 3d | 73.6% | 7d | 100.0% | 8d | 46.7% | 7d | 68.6% | 7d | 100.0% |
|  | 10d | 11.3% | 3.5d | 12.9% |  |  | 9d | 20.8% | 8d | 14.8% |  |  |
|  | 14d | 16.2% | 4d | 13.6% |  |  | 10d | 13.8% | 9d | 6.7% |  |  |
|  | 21d | 1.0% |  |  |  |  | 11d | 8.7% | 10d | 4.3% |  |  |
|  |  |  |  |  |  |  | 12d | 5.0% | 11d | 2.6% |  |  |
|  |  |  |  |  |  |  | 13d | 4.9% | 12d | 1.5% |  |  |
|  |  |  |  |  |  |  | 14d | 0.0% | 13d | 1.5% |  |  |
|  |  |  |  |  |  |  | 15d | 0.0% | 14d | 0.0% |  |  |
|  |  |  |  |  |  |  | 16d | 0.0% | 15d | 0.0% |  |  |
|  |  |  |  |  |  |  | 17d | 0.0% | 16d | 0.0% |  |  |
|  |  |  |  |  |  |  | 18d | 0.0% | 17d | 0.0% |  |  |
|  |  |  |  |  |  |  | 19d | 0.0% | 18d | 0.0% |  |  |
|  |  |  |  |  |  |  | 20d | 0.0% | 19d | 0.0% |  |  |
|  |  |  |  |  |  |  | 21d | 0.0% | 20d | 0.0% |  |  |
|  |  |  |  |  |  |  |  |  | 21d | 0.0% |  |  |
| **Ratio between weekly dose (IU/kg) FIX product and rIX-FP** Median (IQR) |  | | 5.2 | | 1.7 | | 2.5 | | 2.0 | | 0.3 | |
|  |  | | (4.1–5.2) | | (1.4–2.3) | | (2.0–2.5) | | (1.5–2.5) | | (0.3–0.3) | |
| Mean (SD) |  | | 4.8 | | 1.8 | | 2.4 | | 2.0 | | 0.3 | |
|  |  | | (1.2) | | (0.6) | | (0.5) | | (0.6) | | (0.1) | |
| ^Ⱡ^Patients were randomly assigned to individualized interval prophylaxis from a Bernoulli distribution with a probability 0.315, based on data from clinical trial 998HB102 (NCT01027364). In the simulations underlying the results of this table, 68.6% of the simulated patients were assigned to receive weekly prophylaxis and 31.4% to individualized interval prophylaxis. | | | | | | | | | | | | |

| **Patients ≥18 \| Target 3%; Control 20%** |  |  |  |  |  |  |  |  |  |  |  |  |
| --- | --- | --- | --- | --- | --- | --- | --- | --- | --- | --- | --- | --- |
|  | **rIX-FP** | | **rFIX** | | **rFIXFc** weekly | | **rFIXFc** ind. Interval | | **rFIXFc^Ⱡ^** weekly and ind.interval | | **N9-GP** | |
| **Steady-state FIX trough levels** Median (IQR) | 11.7 | | 2.8 | | 3.0 | | 3.2 | | 3.0 | | 4.1 | |
|  | (8.6–14.9) | | (1.6–3.0) | | (3.0–3.0) | | (3.0–3.5) | | (3.0–3.0) | | (3.4–5.1) | |
| Mean (SD) | 11.8 | | 2.3 | | 2.9 | | 3.1 | | 3.0 | | 4.4 | |
|  | (4.2) | | (0.9) | | (0.2) | | (0.6) | | (0.4) | | (1.2) | |
| **Patients below target, %** | 0.0% | | 54.2% | | 13.9% | | 24.6% | | 17.4% | | 0.0% | |
| **Dose (IU/kg per week)** Median (IQR) | 35.0 | | 182.0 | | 58.0 | | 77.8 | | 66.0 | | 10.0 | |
|  | (35.0–35.0) | | (137.0–182.0) | | (45.5–74.5) | | (70.0–87.5) | | (51.0–86.0) | | (10.0–10.0) | |
| Mean (SD) | 34.5 | | 159.3 | | 59.8 | | 77.9 | | 65.4 | | 10.4 | |
|  | (2.3) | | (31.2) | | (18.1) | | (10.8) | | (18.3) | | (1.4) | |
| **Patients among dose-intervals, %** | 7d | 89.9% | 3d | 73.6% | 7d | 100.0% | 8d | 46.7% | 7d | 68.6% | 7d | 100.0% |
|  | 10d | 4.8% | 3.5d | 12.9% |  |  | 9d | 20.8% | 8d | 14.8% |  |  |
|  | 14d | 5.2% | 4d | 13.6% |  |  | 10d | 13.8% | 9d | 6.7% |  |  |
|  | 21d | 0.1% |  |  |  |  | 11d | 8.7% | 10d | 4.3% |  |  |
|  |  |  |  |  |  |  | 12d | 5.0% | 11d | 2.6% |  |  |
|  |  |  |  |  |  |  | 13d | 4.9% | 12d | 1.5% |  |  |
|  |  |  |  |  |  |  | 14d | 0.0% | 13d | 1.5% |  |  |
|  |  |  |  |  |  |  | 15d | 0.0% | 14d | 0.0% |  |  |
|  |  |  |  |  |  |  | 16d | 0.0% | 15d | 0.0% |  |  |
|  |  |  |  |  |  |  | 17d | 0.0% | 16d | 0.0% |  |  |
|  |  |  |  |  |  |  | 18d | 0.0% | 17d | 0.0% |  |  |
|  |  |  |  |  |  |  | 19d | 0.0% | 18d | 0.0% |  |  |
|  |  |  |  |  |  |  | 20d | 0.0% | 19d | 0.0% |  |  |
|  |  |  |  |  |  |  | 21d | 0.0% | 20d | 0.0% |  |  |
|  |  |  |  |  |  |  |  |  | 21d | 0.0% |  |  |
| **Ratio between weekly dose (IU/kg) FIX product and rIX-FP** Median (IQR) |  | | 5.2 | | 1.7 | | 2.2 | | 1.9 | | 0.3 | |
|  |  | | (4.0–5.2) | | (1.3–2.2) | | (2.0–2.5) | | (1.5–2.5) | | (0.3–0.3) | |
| Mean (SD) |  | | 4.6 | | 1.7 | | 2.3 | | 1.9 | | 0.3 | |
|  |  | | (1.0) | | (0.6) | | (0.4) | | (0.6) | | (0.0) | |
| ^Ⱡ^Patients were randomly assigned to individualized interval prophylaxis from a Bernoulli distribution with a probability 0.315, based on data from clinical trial 998HB102 (NCT01027364). In the simulations underlying the results of this table, 68.6% of the simulated patients were assigned to receive weekly prophylaxis and 31.4% to individualized interval prophylaxis. | | | | | | | | | | | | |

| **Patients ≥18 \| Target 5%; Control 7.5%** |  |  |  |  |  |  |  |  |  |  |  |  |
| --- | --- | --- | --- | --- | --- | --- | --- | --- | --- | --- | --- | --- |
|  | **rIX-FP** | | **rFIX** | | **rFIXFc** weekly | | **rFIXFc** ind. Interval | | **rFIXFc^Ⱡ^** weekly and ind.interval | | **N9-GP** | |
| **Steady-state FIX trough levels** Median (IQR) | 6.2 | | 2.8 | | 4.6 | | 4.0 | | 4.4 | | 5.1 | |
|  | (5.1–7.3) | | (1.6–4.5) | | (3.6–5.0) | | (3.0–5.1) | | (3.4–5.0) | | (5.1–5.2) | |
| Mean (SD) | 7.0 | | 2.9 | | 4.2 | | 4.0 | | 4.1 | | 5.3 | |
|  | (2.7) | | (1.5) | | (0.9) | | (1.3) | | (1.1) | | (0.6) | |
| **Patients below target, %** | 0.4% | | 79.9% | | 59.3% | | 71.9% | | 63.4% | | 0.0% | |
| **Dose (IU/kg per week)** Median (IQR) | 33.3 | | 182.0 | | 88.0 | | 87.5 | | 87.5 | | 12.5 | |
|  | (25.2–35.0) | | (182.0–182.0) | | (75.5–88.0) | | (87.5–87.5) | | (80.5–88.0) | | (10.0–15.0) | |
| Mean (SD) | 31 | | 174.7 | | 80.5 | | 85.8 | | 82.2 | | 13.5 | |
|  | (4.7) | | (17.6) | | (12.0) | | (5.0) | | (10.6) | | (4.0) | |
| **Patients among dose-intervals, %** | 7d | 16.9% | 3d | 91.7% | 7d | 100.0% | 8d | 87.4% | 7d | 68.6% | 7d | 100.0% |
|  | 10d | 14.8% | 3.5d | 5.1% |  |  | 9d | 7.7% | 8d | 27.7% |  |  |
|  | 14d | 47.9% | 4d | 3.1% |  |  | 10d | 3.3% | 9d | 2.2% |  |  |
|  | 21d | 20.4% |  |  |  |  | 11d | 1.2% | 10d | 1.0% |  |  |
|  |  |  |  |  |  |  | 12d | 0.3% | 11d | 0.4% |  |  |
|  |  |  |  |  |  |  | 13d | 0.0% | 12d | 0.1% |  |  |
|  |  |  |  |  |  |  | 14d | 0.1% | 13d | 0.0% |  |  |
|  |  |  |  |  |  |  | 15d | 0.0% | 14d | 0.0% |  |  |
|  |  |  |  |  |  |  | 16d | 0.0% | 15d | 0.0% |  |  |
|  |  |  |  |  |  |  | 17d | 0.0% | 16d | 0.0% |  |  |
|  |  |  |  |  |  |  | 18d | 0.0% | 17d | 0.0% |  |  |
|  |  |  |  |  |  |  | 19d | 0.0% | 18d | 0.0% |  |  |
|  |  |  |  |  |  |  | 20d | 0.0% | 19d | 0.0% |  |  |
|  |  |  |  |  |  |  | 21d | 0.0% | 20d | 0.0% |  |  |
|  |  |  |  |  |  |  |  |  | 21d | 0.0% |  |  |
| **Ratio between weekly dose (IU/kg) FIX product and rIX-FP** Median (IQR) |  | | 5.5 | | 2.6 | | 2.6 | | 2.6 | | 0.4 | |
|  |  | | (5.2–6.8) | | (2.4–3.1) | | (2.5–3.3) | | (2.5–3.2) | | (0.3–0.5) | |
| Mean (SD) |  | | 5.8 | | 2.7 | | 2.8 | | 2.7 | | 0.4 | |
|  |  | | (1.0) | | (0.6) | | (0.5) | | (0.5) | | (0.2) | |
| ^Ⱡ^Patients were randomly assigned to individualized interval prophylaxis from a Bernoulli distribution with a probability 0.315, based on data from clinical trial 998HB102 (NCT01027364). In the simulations underlying the results of this table, 68.6% of the simulated patients were assigned to receive weekly prophylaxis and 31.4% to individualized interval prophylaxis. | | | | | | | | | | | | |

| **Patients ≥18 \| Target 5%; Control 10%** |  |  |  |  |  |  |  |  |  |  |  |  |
| --- | --- | --- | --- | --- | --- | --- | --- | --- | --- | --- | --- | --- |
|  | **rIX-FP** | | **rFIX** | | **rFIXFc** weekly | | **rFIXFc** ind. Interval | | **rFIXFc^Ⱡ^** weekly and ind.interval | | **N9-GP** | |
| **Steady-state FIX trough levels** Median (IQR) | 7.9 | | 2.8 | | 4.6 | | 4.0 | | 4.4 | | 5.1 | |
|  | (6.2–9.2) | | (1.6–4.5) | | (3.6–5.0) | | (3.0–5.1) | | (3.4–5.0) | | (5.1–5.2) | |
| Mean (SD) | 8.1 | | 2.9 | | 4.2 | | 4.0 | | 4.1 | | 5.3 | |
|  | (2.6) | | (1.5) | | (0.9) | | (1.2) | | (1.0) | | (0.6) | |
| **Patients below target, %** | 0.4% | | 79.9% | | 59.3% | | 71.9% | | 63.4% | | 0.0% | |
| **Dose (IU/kg per week)** Median (IQR) | 35.0 | | 182.0 | | 88.0 | | 87.5 | | 87.5 | | 12.5 | |
|  | (25.0–35.0) | | (182.0–182.0) | | (75.5–88.0) | | (87.5–87.5) | | (80.5–88.0) | | (10.0–15.0) | |
| Mean (SD) | 31.3 | | 174.7 | | 80.5 | | 85.7 | | 82.1 | | 13.5 | |
|  | (5.2) | | (17.6) | | (12.0) | | (5.1) | | (10.7) | | (4.0) | |
| **Patients among dose-intervals, %** | 7d | 35.9% | 3d | 91.7% | 7d | 100.0% | 8d | 87.4% | 7d | 68.6% | 7d | 100.0% |
|  | 10d | 17.1% | 3.5d | 5.1% |  |  | 9d | 7.7% | 8d | 27.7% |  |  |
|  | 14d | 38.3% | 4d | 3.1% |  |  | 10d | 3.1% | 9d | 2.2% |  |  |
|  | 21d | 8.7% |  |  |  |  | 11d | 1.2% | 10d | 0.9% |  |  |
|  |  |  |  |  |  |  | 12d | 0.4% | 11d | 0.4% |  |  |
|  |  |  |  |  |  |  | 13d | 0.2% | 12d | 0.1% |  |  |
|  |  |  |  |  |  |  | 14d | 0.1% | 13d | 0.0% |  |  |
|  |  |  |  |  |  |  | 15d | 0.0% | 14d | 0.0% |  |  |
|  |  |  |  |  |  |  | 16d | 0.0% | 15d | 0.0% |  |  |
|  |  |  |  |  |  |  | 17d | 0.0% | 16d | 0.0% |  |  |
|  |  |  |  |  |  |  | 18d | 0.0% | 17d | 0.0% |  |  |
|  |  |  |  |  |  |  | 19d | 0.0% | 18d | 0.0% |  |  |
|  |  |  |  |  |  |  | 20d | 0.0% | 19d | 0.0% |  |  |
|  |  |  |  |  |  |  | 21d | 0.0% | 20d | 0.0% |  |  |
|  |  |  |  |  |  |  |  |  | 21d | 0.0% |  |  |
| **Ratio between weekly dose (IU/kg) FIX product and rIX-FP** Median (IQR) |  | | 5.2 | | 2.5 | | 2.5 | | 2.5 | | 0.4 | |
|  |  | | (5.2–7.3) | | (2.3–3.3) | | (2.5–3.5) | | (2.5–3.5) | | (0.3–0.5) | |
| Mean (SD) |  | | 5.7 | | 2.6 | | 2.8 | | 2.7 | | 0.4 | |
|  |  | | (1.1) | | (0.6) | | (0.5) | | (0.6) | | (0.2) | |
| ^Ⱡ^Patients were randomly assigned to individualized interval prophylaxis from a Bernoulli distribution with a probability 0.315, based on data from clinical trial 998HB102 (NCT01027364). In the simulations underlying the results of this table, 68.6% of the simulated patients were assigned to receive weekly prophylaxis and 31.4% to individualized interval prophylaxis. | | | | | | | | | | | | |

| **Patients ≥18 \| Target 5%; Control 15%** |  |  |  |  |  |  |  |  |  |  |  |  |
| --- | --- | --- | --- | --- | --- | --- | --- | --- | --- | --- | --- | --- |
|  | **rIX-FP** | | **rFIX** | | **rFIXFc** weekly | | **rFIXFc** ind. Interval | | **rFIXFc^Ⱡ^** weekly and ind.interval | | **N9-GP** | |
| **Steady-state FIX trough levels** Median (IQR) | 10.5 | | 2.8 | | 4.6 | | 4.0 | | 4.4 | | 5.1 | |
|  | (8.3–13.0) | | (1.6–4.5) | | (3.6–5.0) | | (3.0–5.1) | | (3.4–5.0) | | (5.1–5.2) | |
| Mean (SD) | 10.5 | | 2.9 | | 4.2 | | 4.0 | | 4.1 | | 5.3 | |
|  | (3.0) | | (1.5) | | (0.9) | | (1.2) | | (1.0) | | (0.6) | |
| **Patients below target, %** | 0.4% | | 79.9% | | 59.3% | | 71.9% | | 63.4% | | 0.0% | |
| **Dose (IU/kg per week)** Median (IQR) | 35.0 | | 182.0 | | 88.0 | | 87.5 | | 87.5 | | 12.5 | |
|  | (35.0–35.0) | | (182.0–182.0) | | (75.5–88.0) | | (87.5–87.5) | | (80.5–88.0) | | (10.0–15.0) | |
| Mean (SD) | 33.6 | | 174.7 | | 80.5 | | 85.7 | | 82.1 | | 13.5 | |
|  | (4.1) | | (17.6) | | (12.0) | | (5.1) | | (10.7) | | (4.0) | |
| **Patients among dose-intervals, %** | 7d | 71.5% | 3d | 91.7% | 7d | 100.0% | 8d | 87.4% | 7d | 68.6% | 7d | 100.0% |
|  | 10d | 11.3% | 3.5d | 5.1% |  |  | 9d | 7.7% | 8d | 27.7% |  |  |
|  | 14d | 16.2% | 4d | 3.1% |  |  | 10d | 3.1% | 9d | 2.2% |  |  |
|  | 21d | 1.0% |  |  |  |  | 11d | 1.2% | 10d | 0.9% |  |  |
|  |  |  |  |  |  |  | 12d | 0.4% | 11d | 0.4% |  |  |
|  |  |  |  |  |  |  | 13d | 0.2% | 12d | 0.1% |  |  |
|  |  |  |  |  |  |  | 14d | 0.0% | 13d | 0.1% |  |  |
|  |  |  |  |  |  |  | 15d | 0.0% | 14d | 0.0% |  |  |
|  |  |  |  |  |  |  | 16d | 0.0% | 15d | 0.0% |  |  |
|  |  |  |  |  |  |  | 17d | 0.0% | 16d | 0.0% |  |  |
|  |  |  |  |  |  |  | 18d | 0.0% | 17d | 0.0% |  |  |
|  |  |  |  |  |  |  | 19d | 0.0% | 18d | 0.0% |  |  |
|  |  |  |  |  |  |  | 20d | 0.0% | 19d | 0.0% |  |  |
|  |  |  |  |  |  |  | 21d | 0.0% | 20d | 0.0% |  |  |
|  |  |  |  |  |  |  |  |  | 21d | 0.0% |  |  |
| **Ratio between weekly dose (IU/kg) FIX product and rIX-FP** Median (IQR) |  | | 5.2 | | 2.5 | | 2.5 | | 2.5 | | 0.4 | |
|  |  | | (5.2–5.2) | | (2.2–2.5) | | (2.5–2.5) | | (2.3–2.5) | | (0.3–0.5) | |
| Mean (SD) |  | | 5.3 | | 2.4 | | 2.6 | | 2.5 | | 0.4 | |
|  |  | | (0.9) | | (0.5) | | (0.4) | | (0.5) | | (0.1) | |
| ^Ⱡ^Patients were randomly assigned to individualized interval prophylaxis from a Bernoulli distribution with a probability 0.315, based on data from clinical trial 998HB102 (NCT01027364). In the simulations underlying the results of this table, 68.6% of the simulated patients were assigned to receive weekly prophylaxis and 31.4% to individualized interval prophylaxis. | | | | | | | | | | | | |

| **Patients ≥18 \| Target 5%; Control 20%** |  |  |  |  |  |  |  |  |  |  |  |  |
| --- | --- | --- | --- | --- | --- | --- | --- | --- | --- | --- | --- | --- |
|  | **rIX-FP** | | **rFIX** | | **rFIXFc** weekly | | **rFIXFc** ind. Interval | | **rFIXFc^Ⱡ^** weekly and ind.interval | | **N9-GP** | |
| **Steady-state FIX trough levels** Median (IQR) | 11.7 | | 2.8 | | 4.6 | | 4.0 | | 4.4 | | 5.1 | |
|  | (8.6–14.9) | | (1.6–4.5) | | (3.6–5.0) | | (3.0–5.1) | | (3.4–5.0) | | (5.1–5.2) | |
| Mean (SD) | 11.9 | | 2.9 | | 4.2 | | 4.0 | | 4.1 | | 5.3 | |
|  | (4.1) | | (1.5) | | (0.9) | | (1.2) | | (1.0) | | (0.6) | |
| **Patients below target, %** | 0.4% | | 79.9% | | 59.3% | | 71.9% | | 63.4% | | 0.0% | |
| **Dose (IU/kg per week)** Median (IQR) | 35.0 | | 182.0 | | 88.0 | | 87.5 | | 87.5 | | 12.5 | |
|  | (35.0–35.0) | | (182.0–182.0) | | (75.5–88.0) | | (87.5–87.5) | | (80.5–88.0) | | (10.0–15.0) | |
| Mean (SD) | 34.7 | | 174.7 | | 80.5 | | 85.7 | | 82.1 | | 13.5 | |
|  | (2.7) | | (17.6) | | (12.0) | | (5.1) | | (10.7) | | (4.0) | |
| **Patients among dose-intervals, %** | 7d | 89.9% | 3d | 91.7% | 7d | 100.0% | 8d | 87.4% | 7d | 68.6% | 7d | 100.0% |
|  | 10d | 4.8% | 3.5d | 5.1% |  |  | 9d | 7.7% | 8d | 27.7% |  |  |
|  | 14d | 5.2% | 4d | 3.1% |  |  | 10d | 3.1% | 9d | 2.2% |  |  |
|  | 21d | 0.1% |  |  |  |  | 11d | 1.2% | 10d | 0.9% |  |  |
|  |  |  |  |  |  |  | 12d | 0.4% | 11d | 0.4% |  |  |
|  |  |  |  |  |  |  | 13d | 0.2% | 12d | 0.1% |  |  |
|  |  |  |  |  |  |  | 14d | 0.0% | 13d | 0.1% |  |  |
|  |  |  |  |  |  |  | 15d | 0.0% | 14d | 0.0% |  |  |
|  |  |  |  |  |  |  | 16d | 0.0% | 15d | 0.0% |  |  |
|  |  |  |  |  |  |  | 17d | 0.0% | 16d | 0.0% |  |  |
|  |  |  |  |  |  |  | 18d | 0.0% | 17d | 0.0% |  |  |
|  |  |  |  |  |  |  | 19d | 0.0% | 18d | 0.0% |  |  |
|  |  |  |  |  |  |  | 20d | 0.0% | 19d | 0.0% |  |  |
|  |  |  |  |  |  |  | 21d | 0.0% | 20d | 0.0% |  |  |
|  |  |  |  |  |  |  |  |  | 21d | 0.0% |  |  |
| **Ratio between weekly dose (IU/kg) FIX product and rIX-FP** Median (IQR) |  | | 5.2 | | 2.5 | | 2.5 | | 2.5 | | 0.4 | |
|  |  | | (5.2–5.2) | | (2.1–2.5) | | (2.5–2.5) | | (2.3–2.5) | | (0.3–0.4) | |
| Mean (SD) |  | | 5.1 | | 2.3 | | 2.5 | | 2.4 | | 0.4 | |
|  |  | | (0.7) | | (0.4) | | (0.3) | | (0.4) | | (0.1) | |
| ^Ⱡ^Patients were randomly assigned to individualized interval prophylaxis from a Bernoulli distribution with a probability 0.315, based on data from clinical trial 998HB102 (NCT01027364). In the simulations underlying the results of this table, 68.6% of the simulated patients were assigned to receive weekly prophylaxis and 31.4% to individualized interval prophylaxis). | | | | | | | | | | | | |

| **Patients ≥18 \| Target 7.5%; Control 10%** | |  |  |  |  |  |  |  |  |  |  |  |
| --- | --- | --- | --- | --- | --- | --- | --- | --- | --- | --- | --- | --- |
|  | **rIX-FP** | | **rFIX** | | **rFIXFc** weekly | | **rFIXFc** ind. Interval | | **rFIXFc^Ⱡ^** weekly and ind.interval | | **N9-GP** | |
| **Steady-state FIX trough levels** Median (IQR) | 7.9 | | 2.8 | | 4.6 | | 4.0 | | 4.4 | | 7.6 | |
|  | (7.6–9.2) | | (1.6–4.5) | | (3.6–5.9) | | (3.0–5.2) | | (3.4–5.7) | | (7.5–7.7) | |
| Mean (SD) | 8.7 | | 3.2 | | 4.7 | | 4.2 | | 4.6 | | 7.6 | |
|  | (2.1) | | (2.0) | | (1.6) | | (1.6) | | (1.6) | | (0.1) | |
| **Patients below target, %** | 3.1% | | 93.5% | | 91.5% | | 95.2% | | 92.6% | | 0.0% | |
| **Dose (IU/kg per week)** Median (IQR) | 35.0 | | 182.0 | | 88.0 | | 87.5 | | 88.0 | | 18.5 | |
|  | (29.5–35.0) | | (182.0–182.0) | | (88.0–88.0) | | (87.5–87.5) | | (87.5–88.0) | | (15.0–22.5) | |
| Mean (SD) | 33.5 | | 180.2 | | 87 | | 87.4 | | 87.1 | | 19.6 | |
|  | (5.8) | | (8.2) | | (4.2) | | (1.4) | | (3.6) | | (6.5) | |
| **Patients among dose-intervals, %** | 7d | 35.9% | 3d | 98.3% | 7d | 100.0% | 8d | 98.8% | 7d | 68.6% | 7d | 100.0% |
|  | 10d | 17.1% | 3.5d | 1.2% |  |  | 9d | 0.9% | 8d | 31.0% |  |  |
|  | 14d | 38.3% | 4d | 0.5% |  |  | 10d | 0.3% | 9d | 0.3% |  |  |
|  | 21d | 8.7% |  |  |  |  | 11d | 0.0% | 10d | 0.1% |  |  |
|  |  |  |  |  |  |  | 12d | 0.0% | 11d | 0.0% |  |  |
|  |  |  |  |  |  |  | 13d | 0.0% | 12d | 0.0% |  |  |
|  |  |  |  |  |  |  | 14d | 0.0% | 13d | 0.0% |  |  |
|  |  |  |  |  |  |  | 15d | 0.0% | 14d | 0.0% |  |  |
|  |  |  |  |  |  |  | 16d | 0.0% | 15d | 0.0% |  |  |
|  |  |  |  |  |  |  | 17d | 0.0% | 16d | 0.0% |  |  |
|  |  |  |  |  |  |  | 18d | 0.0% | 17d | 0.0% |  |  |
|  |  |  |  |  |  |  | 19d | 0.0% | 18d | 0.0% |  |  |
|  |  |  |  |  |  |  | 20d | 0.0% | 19d | 0.0% |  |  |
|  |  |  |  |  |  |  | 21d | 0.0% | 20d | 0.0% |  |  |
|  |  |  |  |  |  |  |  |  | 21d | 0.0% |  |  |
| **Ratio between weekly dose (IU/kg) FIX product and rIX-FP** Median (IQR) |  | | 5.2 | | 2.5 | | 2.5 | | 2.5 | | 0.6 | |
|  |  | | (5.2–6.1) | | (2.5–2.9) | | (2.5–2.9) | | (2.5–2.9) | | (0.4–0.7) | |
| Mean (SD) |  | | 5.5 | | 2.7 | | 2.7 | | 2.7 | | 0.6 | |
|  |  | | (1.0) | | (0.5) | | (0.5) | | (0.5) | | (0.2) | |
| ^Ⱡ^Patients were randomly assigned to individualized interval prophylaxis from a Bernoulli distribution with a probability 0.315, based on data from clinical trial 998HB102 (NCT01027364). In the simulations underlying the results of this table, 68.6% of the simulated patients were assigned to receive weekly prophylaxis and 31.4% to individualized interval prophylaxis. | | | | | | | | | | | | |

| **Patients ≥18 \| Target 7.5%; Control 15%** | |  |  |  |  |  |  |  |  |  |  |  |
| --- | --- | --- | --- | --- | --- | --- | --- | --- | --- | --- | --- | --- |
|  | **rIX-FP** | | **rFIX** | | **rFIXFc** weekly | | **rFIXFc** ind. Interval | | **rFIXFc^Ⱡ^** weekly and ind.interval | | **N9-GP** | |
| **Steady-state FIX trough levels** Median (IQR) | 10.5 | | 2.8 | | 4.6 | | 4.0 | | 4.4 | | 7.6 | |
|  | (8.3–13.0) | | (1.6–4.5) | | (3.6–5.9) | | (3.0–5.2) | | (3.4–5.7) | | (7.5–7.7) | |
| Mean (SD) | 10.7 | | 3.2 | | 4.7 | | 4.2 | | 4.6 | | 7.6 | |
|  | (2.7) | | (2.0) | | (1.6) | | (1.6) | | (1.6) | | (0.1) | |
| **Patients below target, %** | 3.1% | | 93.5% | | 91.5% | | 95.2% | | 92.6% | | 0.0% | |
| **Dose (IU/kg per week)** Median (IQR) | 35.0 | | 182.0 | | 88.0 | | 87.5 | | 88.0 | | 18.5 | |
|  | (35.0–35.0) | | (182.0–182.0) | | (88.0–88.0) | | (87.5–87.5) | | (87.5–88.0) | | (15.0–22.5) | |
| Mean (SD) | 34.6 | | 180.2 | | 87 | | 87.3 | | 87.1 | | 19.6 | |
|  | (5.5) | | (8.2) | | (4.2) | | (1.5) | | (3.6) | | (6.5) | |
| **Patients among dose-intervals, %** | 7d | 71.5% | 3d | 98.3% | 7d | 100.0% | 8d | 98.8% | 7d | 68.6% | 7d | 100.0% |
|  | 10d | 11.3% | 3.5d | 1.2% |  |  | 9d | 0.9% | 8d | 31.0% |  |  |
|  | 14d | 16.2% | 4d | 0.5% |  |  | 10d | 0.2% | 9d | 0.3% |  |  |
|  | 21d | 1.0% |  |  |  |  | 11d | 0.1% | 10d | 0.1% |  |  |
|  |  |  |  |  |  |  | 12d | 0.0% | 11d | 0.0% |  |  |
|  |  |  |  |  |  |  | 13d | 0.0% | 12d | 0.0% |  |  |
|  |  |  |  |  |  |  | 14d | 0.0% | 13d | 0.0% |  |  |
|  |  |  |  |  |  |  | 15d | 0.0% | 14d | 0.0% |  |  |
|  |  |  |  |  |  |  | 16d | 0.0% | 15d | 0.0% |  |  |
|  |  |  |  |  |  |  | 17d | 0.0% | 16d | 0.0% |  |  |
|  |  |  |  |  |  |  | 18d | 0.0% | 17d | 0.0% |  |  |
|  |  |  |  |  |  |  | 19d | 0.0% | 18d | 0.0% |  |  |
|  |  |  |  |  |  |  | 20d | 0.0% | 19d | 0.0% |  |  |
|  |  |  |  |  |  |  | 21d | 0.0% | 20d | 0.0% |  |  |
|  |  |  |  |  |  |  |  |  | 21d | 0.0% |  |  |
| **Ratio between weekly dose (IU/kg) FIX product and rIX-FP** Median (IQR) |  | | 5.2 | | 2.5 | | 2.5 | | 2.5 | | 0.5 | |
|  |  | | (5.2–5.2) | | (2.5–2.5) | | (2.5–2.5) | | (2.5–2.5) | | (0.4–0.7) | |
| Mean (SD) |  | | 5.3 | | 2.6 | | 2.6 | | 2.6 | | 0.6 | |
|  |  | | (0.9) | | (0.5) | | (0.4) | | (0.4) | | (0.2) | |
| ^Ⱡ^Patients were randomly assigned to individualized interval prophylaxis from a Bernoulli distribution with a probability 0.315, based on data from clinical trial 998HB102 (NCT01027364). In the simulations underlying the results of this table, 68.6% of the simulated patients were assigned to receive weekly prophylaxis and 31.4% to individualized interval prophylaxis. | | | | | | | | | | | | |

| **Patients ≥18 \| Target 7.5%; Control 20%** | | | | |  |  |  |  |  |  |  |  |
| --- | --- | --- | --- | --- | --- | --- | --- | --- | --- | --- | --- | --- |
|  | **rIX-FP** | | **rFIX** | | **rFIXFc** weekly | | **rFIXFc** ind. Interval | | **rFIXFc^Ⱡ^** weekly and ind.interval | | **N9-GP** | |
| **Steady-state FIX trough levels** Median (IQR) | 11.7 | | 2.8 | | 4.6 | | 4.0 | | 4.4 | | 7.6 | |
|  | (8.6–14.9) | | (1.6–4.5) | | (3.6–5.9) | | (3.0–5.2) | | (3.4–5.7) | | (7.5–7.7) | |
| Mean (SD) | 12.1 | | 3.2 | | 4.7 | | 4.2 | | 4.6 | | 7.6 | |
|  | (3.8) | | (2.0) | | (1.6) | | (1.6) | | (1.6) | | (0.1) | |
| **Patients below target, %** | 3.1% | | 93.5% | | 91.5% | | 95.2% | | 92.6% | | 0.0% | |
| **Dose (IU/kg per week)** Median (IQR) | 35.0 | | 182.0 | | 88.0 | | 87.5 | | 88.0 | | 18.5 | |
|  | (35.0–35.0) | | (182.0–182.0) | | (88.0–88.0) | | (87.5–87.5) | | (87.5–88.0) | | (15.0–22.5) | |
| Mean (SD) | 35.7 | | 180.2 | | 87 | | 87.3 | | 87.1 | | 19.6 | |
|  | (4.3) | | (8.2) | | (4.2) | | (1.5) | | (3.6) | | (6.5) | |
| **Patients among dose-intervals, %** | 7d | 89.9% | 3d | 98.3% | 7d | 100.0% | 8d | 98.8% | 7d | 68.6% | 7d | 100.0% |
|  | 10d | 4.8% | 3.5d | 1.2% |  |  | 9d | 0.9% | 8d | 31.0% |  |  |
|  | 14d | 5.2% | 4d | 0.5% |  |  | 10d | 0.2% | 9d | 0.3% |  |  |
|  | 21d | 0.1% |  |  |  |  | 11d | 0.1% | 10d | 0.1% |  |  |
|  |  |  |  |  |  |  | 12d | 0.0% | 11d | 0.0% |  |  |
|  |  |  |  |  |  |  | 13d | 0.0% | 12d | 0.0% |  |  |
|  |  |  |  |  |  |  | 14d | 0.0% | 13d | 0.0% |  |  |
|  |  |  |  |  |  |  | 15d | 0.0% | 14d | 0.0% |  |  |
|  |  |  |  |  |  |  | 16d | 0.0% | 15d | 0.0% |  |  |
|  |  |  |  |  |  |  | 17d | 0.0% | 16d | 0.0% |  |  |
|  |  |  |  |  |  |  | 18d | 0.0% | 17d | 0.0% |  |  |
|  |  |  |  |  |  |  | 19d | 0.0% | 18d | 0.0% |  |  |
|  |  |  |  |  |  |  | 20d | 0.0% | 19d | 0.0% |  |  |
|  |  |  |  |  |  |  | 21d | 0.0% | 20d | 0.0% |  |  |
|  |  |  |  |  |  |  |  |  | 21d | 0.0% |  |  |
| **Ratio between weekly dose (IU/kg) FIX product and rIX-FP** Median (IQR) |  | | 5.2 | | 2.5 | | 2.5 | | 2.5 | | 0.5 | |
|  |  | | (5.2–5.2) | | (2.5–2.5) | | (2.5–2.5) | | (2.5–2.5) | | (0.4–0.6) | |
| Mean (SD) |  | | 5.1 | | 2.5 | | 2.5 | | 2.5 | | 0.6 | |
|  |  | | (0.7) | | (0.3) | | (0.3) | | (0.3) | | (0.2) | |
| ^Ⱡ^Patients were randomly assigned to individualized interval prophylaxis from a Bernoulli distribution with a probability 0.315, based on data from clinical trial 998HB102 (NCT01027364). In the simulations underlying the results of this table, 68.6% of the simulated patients were assigned to receive weekly prophylaxis and 31.4% to individualized interval prophylaxis. | | | | | | | | | | | | |

| **Patients ≥18 \| Target 10.0%; Control 15%** | | | | | | |  | |  | |  | |  | |  | |  | |  | |
| --- | --- | --- | --- | --- | --- | --- | --- | --- | --- | --- | --- | --- | --- | --- | --- | --- | --- | --- | --- | --- |
|  | **rIX-FP** | | **rFIX** | | **rFIXFc** weekly | | | | **rFIXFc** ind. Interval | | | | **rFIXFc^Ⱡ^** weekly and ind.interval | | | | **N9-GP** | | | |
| **Steady-state FIX trough levels** Median (IQR) | 10.5 | | 2.8 | | 4.6 | | | | 4.0 | | | | 4.4 | | | | 10.1 | | | |
|  | (10.1–13.0) | | (1.6–4.5) | | (3.6–5.9) | | | | (3.0–5.2) | | | | (3.4–5.7) | | | | (10.0–10.2) | | | |
| Mean (SD) | 11.3 | | 3.3 | | 4.8 | | | | 4.2 | | | | 4.7 | | | | 10.1 | | | |
|  | (2.2) | | (2.2) | | (1.8) | | | | (1.7) | | | | (1.8) | | | | (0.1) | | | |
| **Patients below target, %** | 9.3% | | 98.2% | | 98.7% | | | | 99.4% | | | | 98.9% | | | | 0.0% | | | |
| **Dose (IU/kg per week)** Median (IQR) | 35.0 | | 182.0 | | 88.0 | | | | 87.5 | | | | 88.0 | | | | 24.5 | | | |
|  | (35.0–39.5) | | (182.0–182.0) | | (88.0–88.0) | | | | (87.5–87.5) | | | | (87.5–88.0) | | | | (20.0–30.0) | | | |
| Mean (SD) | 36.7 | | 181.6 | | 87.9 | | | | 87.5 | | | | 87.8 | | | | 26 | | | |
|  | (6.8) | | (3.7) | | (1.4) | | | | (0.5) | | | | (1.1) | | | | (8.8) | | | |
| **Patients among dose-intervals, %** | 7d | 71.5% | 3d | 99.6% | 7d | | 100.0% | | 8d | | 99.9% | | 7d | | 68.6% | | 7d | | 100.0% | |
|  | 10d | 11.3% | 3.5d | 0.3% |  | |  | | 9d | | 0.1% | | 8d | | 31.3% | |  | |  | |
|  | 14d | 16.2% | 4d | 0.1% |  | |  | | 10d | | 0.1% | | 9d | | 0.0% | |  | |  | |
|  | 21d | 1.0% |  |  |  | |  | | 11d | | 0.0% | | 10d | | 0.0% | |  | |  | |
|  |  |  |  |  |  | |  | | 12d | | 0.0% | | 11d | | 0.0% | |  | |  | |
|  |  |  |  |  |  | |  | | 13d | | 0.0% | | 12d | | 0.0% | |  | |  | |
|  |  |  |  |  |  | |  | | 14d | | 0.0% | | 13d | | 0.0% | |  | |  | |
|  |  |  |  |  |  | |  | | 15d | | 0.0% | | 14d | | 0.0% | |  | |  | |
|  |  |  |  |  |  | |  | | 16d | | 0.0% | | 15d | | 0.0% | |  | |  | |
|  |  |  |  |  |  | |  | | 17d | | 0.0% | | 16d | | 0.0% | |  | |  | |
|  |  |  |  |  |  | |  | | 18d | | 0.0% | | 17d | | 0.0% | |  | |  | |
|  |  |  |  |  |  | |  | | 19d | | 0.0% | | 18d | | 0.0% | |  | |  | |
|  |  |  |  |  |  | |  | | 20d | | 0.0% | | 19d | | 0.0% | |  | |  | |
|  |  |  |  |  |  | |  | | 21d | | 0.0% | | 20d | | 0.0% | |  | |  | |
|  |  |  |  |  |  | |  | |  | |  | | 21d | | 0.0% | |  | |  | |
| **Ratio between weekly dose (IU/kg) FIX product and rIX-FP** Median (IQR) |  | | 5.2 | | 2.5 | | | | 2.5 | | | | 2.5 | | | | 0.7 | | | |
|  |  | | (4.6–5.2) | | (2.2–2.5) | | | | (2.2–2.5) | | | | (2.2–2.5) | | | | (0.5–0.9) | | | |
| Mean (SD) |  | | 5.1 | | 2.5 | | | | 2.5 | | | | 2.5 | | | | 0.7 | | | |
|  |  | | (1.0) | | (0.5) | | | | (0.5) | | | | (0.5) | | | | (0.3) | | | |
| ^Ⱡ^Patients were randomly assigned to individualized interval prophylaxis from a Bernoulli distribution with a probability 0.315, based on data from clinical trial 998HB102 (NCT01027364). In the simulations underlying the results of this table, 68.6% of the simulated patients were assigned to receive weekly prophylaxis and 31.4% to individualized interval prophylaxis. | | | | | | | | | | | | | | | | | | | | |
| **Patients ≥18 \| Target 10.0%; Control 20%** | | | | |  |  | |  | |  | |  | |  | |  | |  | |  |
|  | **rIX-FP** | | **rFIX** | | **rFIXFc** weekly | | | **rFIXFc** ind. Interval | | | | **rFIXFc^Ⱡ^** weekly and ind.interval | | | | **N9-GP** | | | |  |
| **Steady-state FIX trough levels** Median (IQR) | 11.7 | | 2.8 | | 4.6 | | | 4.0 | | | | 4.4 | | | | 10.1 | | | |  |
|  | (10.1–14.9) | | (1.6–4.5) | | (3.6–5.9) | | | (3.0–5.2) | | | | (3.4–5.7) | | | | (10.0–10.2) | | | |  |
| Mean (SD) | 12.6 | | 3.3 | | 4.8 | | | 4.2 | | | | 4.7 | | | | 10.1 | | | |  |
|  | (3.3) | | (2.2) | | (1.8) | | | (1.7) | | | | (1.8) | | | | (0.1) | | | |  |
| **Patients below target, %** | 9.3% | | 98.2% | | 98.7% | | | 99.4% | | | | 98.9% | | | | 0.0% | | | |  |
| **Dose (IU/kg per week)** Median (IQR) | 35.0 | | 182.0 | | 88.0 | | | 87.5 | | | | 88.0 | | | | 24.5 | | | |  |
|  | (35.0–39.5) | | (182.0–182.0) | | (88.0–88.0) | | | (87.5–87.5) | | | | (87.5–88.0) | | | | (20.0–30.0) | | | |  |
| Mean (SD) | 37.6 | | 181.6 | | 87.9 | | | 87.5 | | | | 87.8 | | | | 26 | | | |  |
|  | (5.9) | | (3.7) | | (1.4) | | | (0.5) | | | | (1.1) | | | | (8.8) | | | |  |
| **Patients among dose-intervals, %** | 7d | 89.9% | 3d | 99.6% | 7d | 100.0% | | 8d | | 99.9% | | 7d | | 68.6% | | 7d | | 100.0% | |  |
|  | 10d | 4.8% | 3.5d | 0.3% |  |  | | 9d | | 0.1% | | 8d | | 31.3% | |  | |  | |  |
|  | 14d | 5.2% | 4d | 0.1% |  |  | | 10d | | 0.1% | | 9d | | 0.0% | |  | |  | |  |
|  | 21d | 0.1% |  |  |  |  | | 11d | | 0.0% | | 10d | | 0.0% | |  | |  | |  |
|  |  |  |  |  |  |  | | 12d | | 0.0% | | 11d | | 0.0% | |  | |  | |  |
|  |  |  |  |  |  |  | | 13d | | 0.0% | | 12d | | 0.0% | |  | |  | |  |
|  |  |  |  |  |  |  | | 14d | | 0.0% | | 13d | | 0.0% | |  | |  | |  |
|  |  |  |  |  |  |  | | 15d | | 0.0% | | 14d | | 0.0% | |  | |  | |  |
|  |  |  |  |  |  |  | | 16d | | 0.0% | | 15d | | 0.0% | |  | |  | |  |
|  |  |  |  |  |  |  | | 17d | | 0.0% | | 16d | | 0.0% | |  | |  | |  |
|  |  |  |  |  |  |  | | 18d | | 0.0% | | 17d | | 0.0% | |  | |  | |  |
|  |  |  |  |  |  |  | | 19d | | 0.0% | | 18d | | 0.0% | |  | |  | |  |
|  |  |  |  |  |  |  | | 20d | | 0.0% | | 19d | | 0.0% | |  | |  | |  |
|  |  |  |  |  |  |  | | 21d | | 0.0% | | 20d | | 0.0% | |  | |  | |  |
|  |  |  |  |  |  |  | |  | |  | | 21d | | 0.0% | |  | |  | |  |
| **Ratio between weekly dose (IU/kg) FIX product and rIX-FP** Median (IQR) |  | | 5.2 | | 2.5 | | | 2.5 | | | | 2.5 | | | | 0.7 | | | |  |
|  |  | | (4.6–5.2) | | (2.2–2.5) | | | (2.2–2.5) | | | | (2.2–2.5) | | | | (0.5–0.8) | | | |  |
| Mean (SD) |  | | 4.9 | | 2.4 | | | 2.4 | | | | 2.4 | | | | 0.7 | | | |  |
|  |  | | (0.8) | | (0.4) | | | (0.4) | | | | (0.4) | | | | (0.3) | | | |  |
| ^Ⱡ^Patients were randomly assigned to individualized interval prophylaxis from a Bernoulli distribution with a probability 0.315, based on data from clinical trial 998HB102 (NCT01027364). In the simulations underlying the results of this table, 68.6% of the simulated patients were assigned to receive weekly prophylaxis and 31.4% to individualized interval prophylaxis. | | | | | | | | | | | | | | | | | | | |  |
| **Patients ≥18 \| Target 15%; Control 20%** |  |  |  |  |  |  | |  | |  | |  | |  | |  | |  | |  |
|  | **rIX-FP** | | **rFIX** | | **rFIXFc** weekly | | | **rFIXFc** ind. Interval | | | | **rFIXFc^Ⱡ^** weekly and ind.interval | | | | **N9-GP** | | | |  |
| **Steady-state FIX trough levels** Median (IQR) | 15.1 | | 2.8 | | 4.6 | | | 4.0 | | | | 4.4 | | | | 15.1 | | | |  |
|  | (13.6–15.2) | | (1.6–4.5) | | (3.6–5.9) | | | (3.0–5.2) | | | | (3.4–5.7) | | | | (15.0–15.2) | | | |  |
| Mean (SD) | 14.5 | | 3.4 | | 4.9 | | | 4.2 | | | | 4.7 | | | | 15.1 | | | |  |
|  | (2.9) | | (2.4) | | (1.8) | | | (1.7) | | | | (1.8) | | | | (0.1) | | | |  |
| **Patients below target, %** | 32.4% | | 99.8% | | 100.0% | | | 100.0% | | | | 100.0% | | | | 0.0% | | | |  |
| **Dose (IU/kg per week)** Median (IQR) | 42.5 | | 182.0 | | 88.0 | | | 87.5 | | | | 88.0 | | | | 36.5 | | | |  |
|  | (35.0–50.0) | | (182.0–182.0) | | (88.0–88.0) | | | (87.5–87.5) | | | | (87.5–88.0) | | | | (30.0–45.0) | | | |  |
| Mean (SD) | 42.3 | | 182 | | 88 | | | 87.5 | | | | 87.8 | | | | 38.9 | | | |  |
|  | (7.0) | | (0.9) | | (0.2) | | | (0.0) | | | | (0.2) | | | | (13.1) | | | |  |
| **Patients among dose-intervals, %** | 7d | 89.9% | 3d | 100.0% | 7d | 100.0% | | 8d | | 100.0% | | 7d | | 68.6% | | 7d | | 100.0% | |  |
|  | 10d | 4.8% | 3.5d | 0.0% |  |  | | 9d | | 0.0% | | 8d | | 31.4% | |  | |  | |  |
|  | 14d | 5.2% | 4d | 0.0% |  |  | | 10d | | 0.0% | | 9d | | 0.0% | |  | |  | |  |
|  | 21d | 0.1% |  |  |  |  | | 11d | | 0.0% | | 10d | | 0.0% | |  | |  | |  |
|  |  |  |  |  |  |  | | 12d | | 0.0% | | 11d | | 0.0% | |  | |  | |  |
|  |  |  |  |  |  |  | | 13d | | 0.0% | | 12d | | 0.0% | |  | |  | |  |
|  |  |  |  |  |  |  | | 14d | | 0.0% | | 13d | | 0.0% | |  | |  | |  |
|  |  |  |  |  |  |  | | 15d | | 0.0% | | 14d | | 0.0% | |  | |  | |  |
|  |  |  |  |  |  |  | | 16d | | 0.0% | | 15d | | 0.0% | |  | |  | |  |
|  |  |  |  |  |  |  | | 17d | | 0.0% | | 16d | | 0.0% | |  | |  | |  |
|  |  |  |  |  |  |  | | 18d | | 0.0% | | 17d | | 0.0% | |  | |  | |  |
|  |  |  |  |  |  |  | | 19d | | 0.0% | | 18d | | 0.0% | |  | |  | |  |
|  |  |  |  |  |  |  | | 20d | | 0.0% | | 19d | | 0.0% | |  | |  | |  |
|  |  |  |  |  |  |  | | 21d | | 0.0% | | 20d | | 0.0% | |  | |  | |  |
|  |  |  |  |  |  |  | |  | |  | | 21d | | 0.0% | |  | |  | |  |
| **Ratio between weekly dose (IU/kg) FIX product and rIX-FP** Median (IQR) |  | | 4.3 | | 2.1 | | | 2.1 | | | | 2.1 | | | | 0.9 | | | |  |
|  |  | | (3.6–5.2) | | (1.8–2.5) | | | (1.8–2.5) | | | | (1.8–2.5) | | | | (0.7–1.1) | | | |  |
| Mean (SD) |  | | 4.4 | | 2.1 | | | 2.1 | | | | 2.1 | | | | 0.9 | | | |  |
|  |  | | (0.8) | | (0.4) | | | (0.4) | | | | (0.4) | | | | (0.4) | | | |  |
| ^Ⱡ^Patients were randomly assigned to individualized interval prophylaxis from a Bernoulli distribution with a probability 0.315, based on data from clinical trial 998HB102 (NCT01027364). In the simulations underlying the results of this table, 68.6% of the simulated patients were assigned to receive weekly prophylaxis and 31.4% to individualized interval prophylaxis. | | | | | | | | | | | | | | | | | | | |  |
